# Reactivity of a Gold-Aluminyl Complex with Carbon Dioxide: A Nucleophilic Gold?

**DOI:** 10.1021/jacs.1c06728

**Published:** 2021-09-02

**Authors:** Diego Sorbelli, Leonardo Belpassi, Paola Belanzoni

**Affiliations:** † Department of Chemistry, Biology and Biotechnology, University of Perugia, Via Elce di Sotto, 8, 06123 Perugia, Italy; ‡ Istituto CNR di Scienze e Tecnologie Chimiche “Giulio Natta” (CNR-SCITEC), Via Elce di Sotto, 8, 06123 Perugia, Italy; § Computational Laboratory for Hybrid/Organic Photovoltaics (CLHYO) c/o Istituto CNR di Scienze e Tecnologie Chimiche “Giulio Natta” (CNR-SCITEC), Via Elce di Sotto, 8, 06123 Perugia, Italy

## Abstract

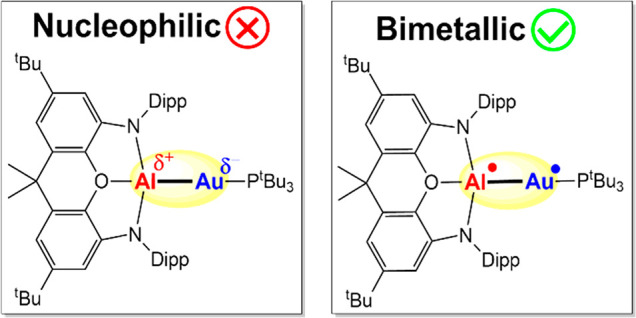

A gold-aluminyl complex has been recently reported to feature an unconventional gold nucleophilic center, which was revealed through reactivity with carbon dioxide leading to the Au-CO_2_ coordination mode. In this work, we computationally investigate the reaction mechanism, which is found to be cooperative, with the gold–aluminum bond being the actual nucleophile and Al also behaving as electrophile. The Au–Al bond is shown to be mainly of an electron-sharing nature, with the two metal fragments displaying a diradical-like reactivity with CO_2_.

Recently, a striking reactivity of CO_2_ with a complex bearing a Au–Al bond has been reported.^1^ A combination of the new generation aluminyl anion^2^ [K{Al(NON)}]_2_ with a phosphine gold ^t^Bu_3_PAuI affords the [^t^Bu_3_PAuAl(NON)] complex (**I**) which, in reaction with CO_2_ (1 atm at room temperature in toluene), leads to the stable [^t^Bu_3_PAuCO_2_Al(NON)] complex (**II**), where Au binds to the CO_2_ carbon atom (Scheme 1). This CO_2_ coordination mode has been considered as the revealing of an unconventional nucleophilic behavior of gold, which, in contrast, is well-known to be an extremely powerful electrophile in organic reactions involving unsaturated CC bonds.^3^ The authors suggested that the aluminyl anion [Al(NON)]^−^ is able to induce an extremely polarized Au(δ-)-Al(δ+) bond, with a significant negative charge at the gold site, which is able to reverse its reactivity. DFT calculations combined with quantum theory of atoms in molecules (QTAIM) charge analysis have shown a substantial electron transfer from [Al(NON)]^−^ to [^t^Bu_3_PAu]^+^ (1.56 electrons) and a negative charge at Au (−0.82).^1^ This picture seems to be consistent with the difference in electronegativity values of the two metals (Au = 2.54, Al = 1.61 on the Pauling scale) and with the relativistic effects on gold which stabilize and contract the 6s orbital,^4^ resulting in the gold highest electron affinity (2.30 eV) among transition metals (other coinage metals have considerably smaller values, Cu 1.23 eV; Ag 1.30 eV).^5^ Complex **I** is not the only complex in which Au would act as a nucleophile toward polar multiple bond.^6^ Two copper-aluminyl complexes (**III** and **V** in Scheme 1) have been reported to insert CO_2_ into the Cu–Al bond, resulting in complexes **IV** and **VI** (Scheme 1), which are very much similar to complex **II** in terms of structure and kinetic stability.^7^ A significant covalency of the Al–Cu bond and only slightly negative charges on Cu (e.g., −0.09 in **III**) have been calculated, revealing here only a small polarization of the M(δ-)-Al(δ+) bond.

**Scheme 1:**
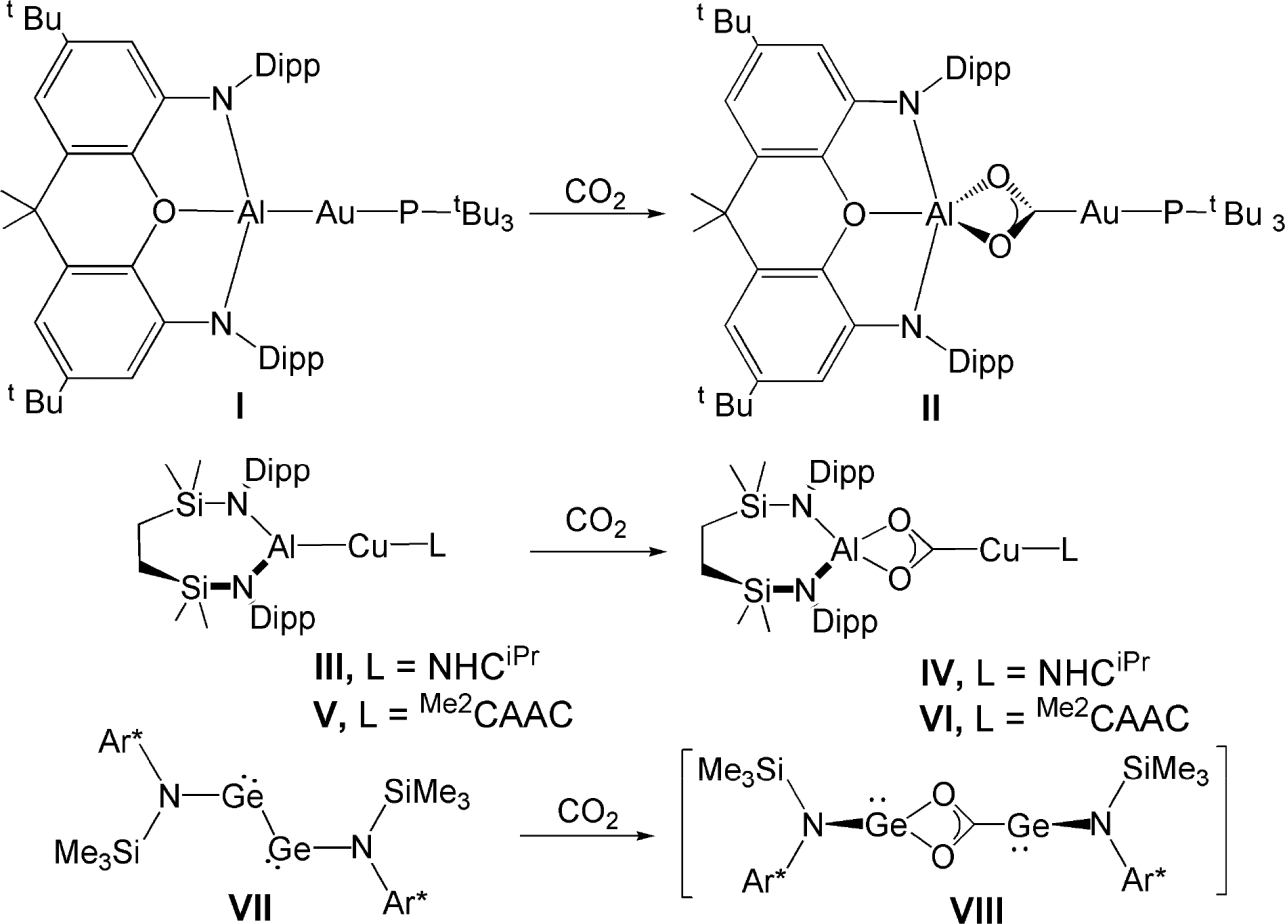
Examples of Nucleophilic Gold (**I**),^1^ Copper (**III**, **V**),^7^ and Amido-Digermyne (**VII**)^12^ Compounds and Their CO_2_ Insertion Reaction Products (**II**, **IV**, **VI**, and **VIII**)

In addition to strongly polarized M(δ-)-Al(δ+) bonds in heterodinuclear complexes,^8^ CO_2_ activation by homodinuclear main-group element species,^9,10^ including diradicals,^11^ is not uncommon. Frenking and Jones^12^ demonstrated that the facile reduction of CO_2_ to CO by a symmetric amido-digermyne compound (**VII**) proceeds through an asymmetrical intermediate (**VIII**) that bears a structural analogy with complex **II**.

The similar coordination modes of CO_2_ in the Au–Al, Cu–Al, and Ge–Ge bonds is eye-catching, in view of the different degrees of polarization of the insertion metal–metal site, which is expected to determine the effectiveness of the metal nucleophilic behavior. This prompted us to computationally investigate the mechanism of the CO_2_ insertion into the pioneering nucleophilic [^t^Bu_3_PAuAl(NON)] complex which has not been explored yet and the actual nucleophilic ability of Au in this “unorthodox reaction “.^13^ We demonstrate that the nucleophilic attack is actually performed by the Au–Al σ bond, revealing a bimetallic (Au/Al) cooperation in the CO_2_ binding. The attack is also assisted by a secondary interaction, involving the partially empty 3p_*x*_ atomic orbital of Al, which exploits its Lewis acidity. Transition state and intermediate structures found here suggest a radical-like insertion of CO_2_ in the Au–Al bond, which has been consistently shown to have an electron-sharing character.

The free energy profile for the CO_2_ insertion into the Au–Al bond of **I** was calculated using density functional theory (DFT) with the inclusion of relativistic effects, solvation, and dispersion interactions (see SI for computational details), and it is shown in Figure 1. Complex **I** was slightly simplified at the NON site (denoted as NON’). The modeling effect is evaluated in Table S1. The optimized geometries of [^t^Bu_3_PAuAl(NON’)], RC, TSI, INT, TSII, and [^t^Bu_3_PAuCO_2_Al(NON’)] (PC) complexes are reported in the SI (Figure S1) and show good agreement with available experimental data (Figure S2 and Table S2).

**Figure 1:**
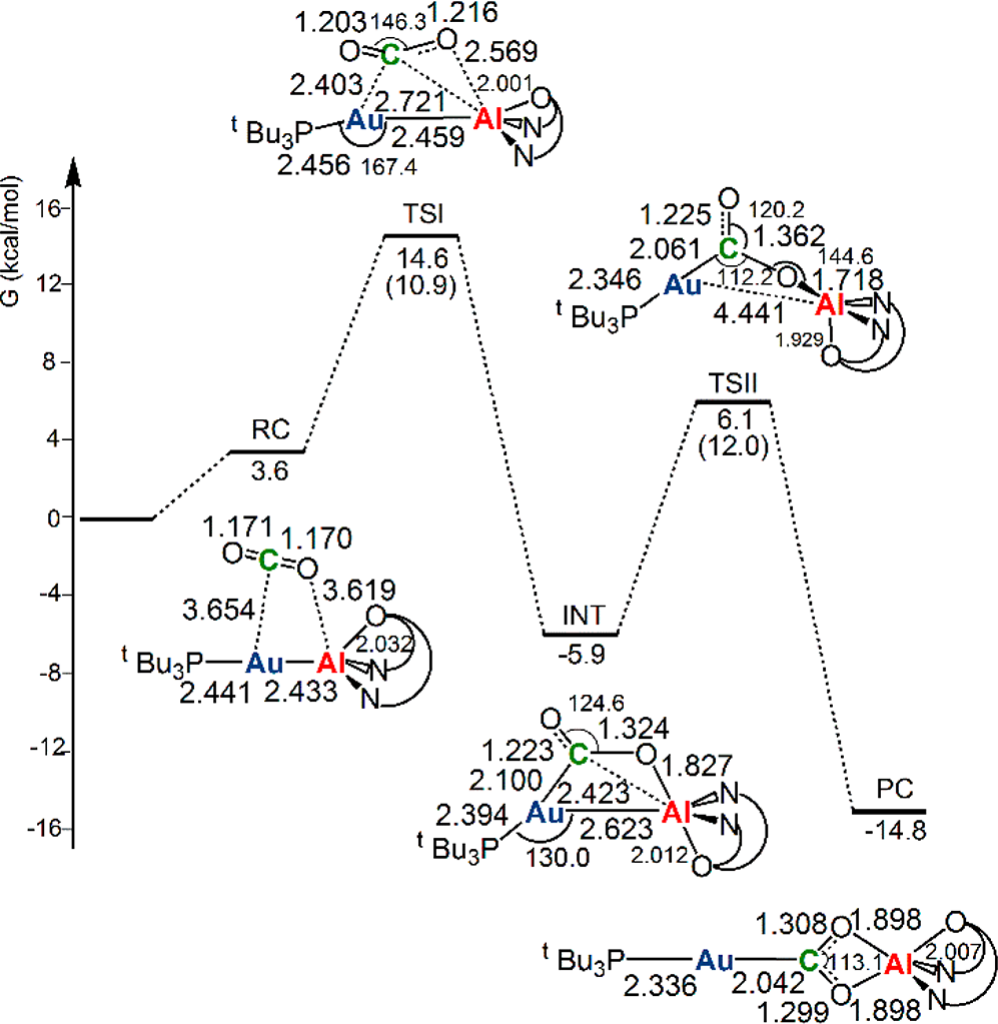
Free energy reaction profile for the CO_2_ insertion into the Au–Al bond in the [^t^Bu_3_PAuAl(NON’)] complex. Δ*G* values refer to the energy of the separated reactants taken as zero. Activation free energy barriers are reported in parentheses. Selected interatomic distances (Å) and bond angles (degrees) are given with the molecular structures.

The nucleophilic attack to the CO_2_ carbon atom has a relatively low activation free energy barrier (Δ*G*^#^ = 10.9 kcal/mol). At the TSI, the carbon atom of CO_2_ is both very close to Au (2.403 Å) and at a relatively short distance from Al (2.721 Å), and a substantial bending of CO_2_ and asymmetry between the two C–O bonds are also observed (see Table S3 for the evolution of the most relevant Mayer’s bond orders along the reaction path). Subsequent complete bonding of CO_2_ carbon atom to Au and oxygen atom to Al leads to the formation of intermediate INT, which is stabilized by 20.5 kcal/mol. As a result, the Au–Al bond distance slightly increases and the ^t^Bu_3_PAu moiety coordinates almost linearly with the carbon atom of CO_2_. A second transition state (TSII) is located with an activation free energy barrier Δ*G*^#^ of 12.0 kcal/mol, showing a substantial breaking of the Au–Al bond. Finally, the oxygen atom of CO_2_ attack to the electrophilic Al center leads to the thermodynamically stable product complex PC. The overall CO_2_ insertion into the Au–Al bond is exergonic by −14.8 kcal/mol.

To get insights into the nature of the CO_2_ insertion process, we analyze the first activation barrier using the Activation Strain Model (ASM)^14−16^ (main results in Figure 2, left panel). The ASM formalism is briefly summarized in the SI, and all the results are reported in Table S4.

**Figure 2:**
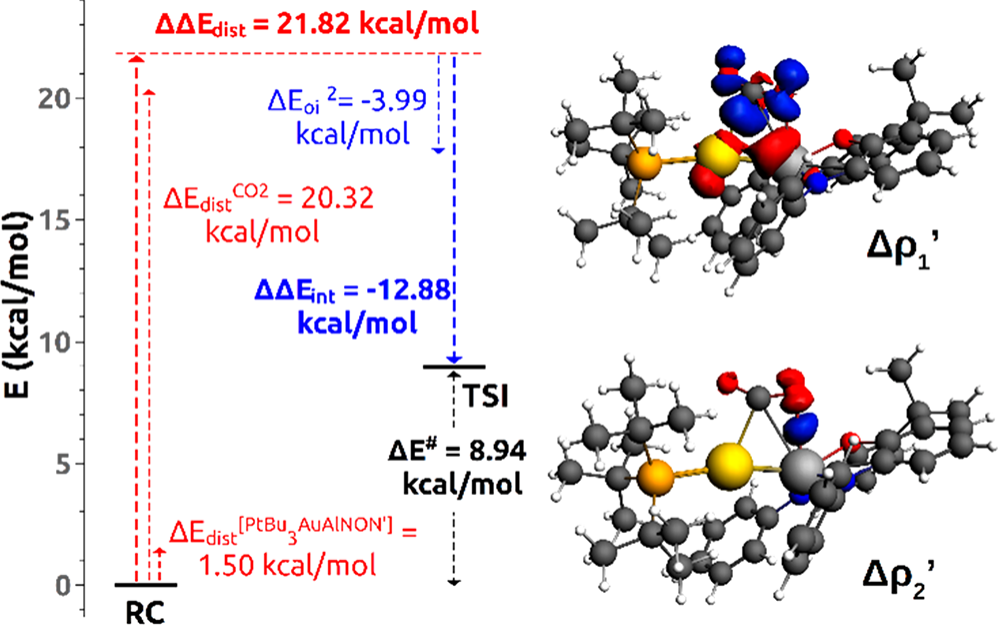
Activation Strain Model (ASM) decomposition of the electronic energy activation barrier Δ*E*^#^ (left) (see text). Isodensity surfaces (2 me/a_0_^3^) for the NOCV deformation density maps (charge flux is red → blue) corresponding to the Δρ_1_’ (top right) and Δρ_2_’ (bottom right) contributions to the CO_2_-[^t^Bu_3_PAuAl(NON’)] fragments interaction in the transition state TSI.

The distortion energy contribution (ΔΔ*E*_dist_ = 21.82 kcal/mol) to the electronic energy activation barrier (Δ*E*^#^=8.94 kcal/mol) is almost completely associated with the CO_2_ bending (20.32 kcal/mol, see Table S5), whereas the stabilizing interaction contribution (ΔΔ*E*_int_= −12.88 kcal/mol) mainly arises from the orbital interaction energy at TSI (−53.30 kcal/mol) (see Table S6). The results of the ETS-NOCV^17^ method coupled with the Charge Displacement (CD) Analysis^18^ (see SI for methodological details) are summarized below. In Figure 2 (right panel), the two most important components (Δρ_1_’ and Δρ_2_’) of the total deformation density are shown.

The main interaction component (Δρ_1_’) is clearly characterized by an electron density depletion localized on both Au and Al atoms and by an electron density accumulation at the CO_2_ site. This component is associated with a significant energy stabilization (−41.20 kcal/mol) and a charge transfer from the Au–Al bond region to carbon dioxide of 0.326 e. The decomposition into the donor and acceptor NOCV orbitals^19^ (Figure S3) shows that the electron density accumulation has the main contribution from the LUMO of CO_2_, while the electron density depletion shows contributions from the HOMO–2 and HOMO of the [^t^Bu_3_PAuAl(NON’)] fragment, both representing the Au–Al σ bond, where Al 3s3p_*z*_–Au 6s6p_*z*_ type orbitals are involved. Component Δρ_2_’ reveals an electron density accumulation at the Al center (note that its shape recalls that of an atomic p_*x*_ orbital), coming from one of the oxygen atoms of CO_2_. Decomposition into donor and acceptor NOCV orbitals (Figure S4) shows that the main contribution to the donor orbital is the HOMO of CO_2_, whereas a clear characterization of the acceptor orbital is less straightforward, since several delocalized unoccupied MOs, all with small Al 3p_*x*_ orbital mixing, contribute to it. The Δρ_2_’ contribution is not negligible: 0.047 e are transferred toward Al from CO_2_, with an associated orbital interaction energy of −3.99 kcal/mol (which notably accounts for one-third of the interaction stabilization to the activation barrier ΔΔ*E*_int_).

The reaction mechanism in Figure 1 bears surprising analogies with the first steps of the reaction profile for the reduction of CO_2_ to CO by [LGe-GeL] (see Figure 3 of ref (12)), although complex **II** does not evolve to CO elimination (the resulting oxide complex [^t^Bu_3_PAuOAl(NON’)][CO] has been calculated to be highly unstable with Δ*G* = 29.8 kcal/mol). The high reactivity of digermynes has been often attributed to the nonnegligible biradical character in these systems.^11,19,20^ A possible diradicaloid character of the Au–Al bond in the [^t^Bu_3_PAuAl(NON)] complex is certainly very intriguing. Remarkably, the coordination modes of CO_2_ in the separated neutral open shell doublet [^t^Bu_3_PAu(CO_2_)]· and [CO_2_Al(NON’)]· fragments closely match those found at the TSI and INT (Figures S5, S6 and discussion therein). This prompted us to review the Au–Al bond nature in complex **I**. We carry out the CD-NOCV analysis on the [^t^Bu_3_PAuAl(NON’)] complex by choosing the open-shell radical fragments [^t^Bu_3_PAu]· and [(NON’)Al]· on the basis of EDA analysis^21^ using different fragmentations (Table S7 and ref (22)). The main results of the CD-NOCV analysis are reported in Figure 3.

**Figure 3:**
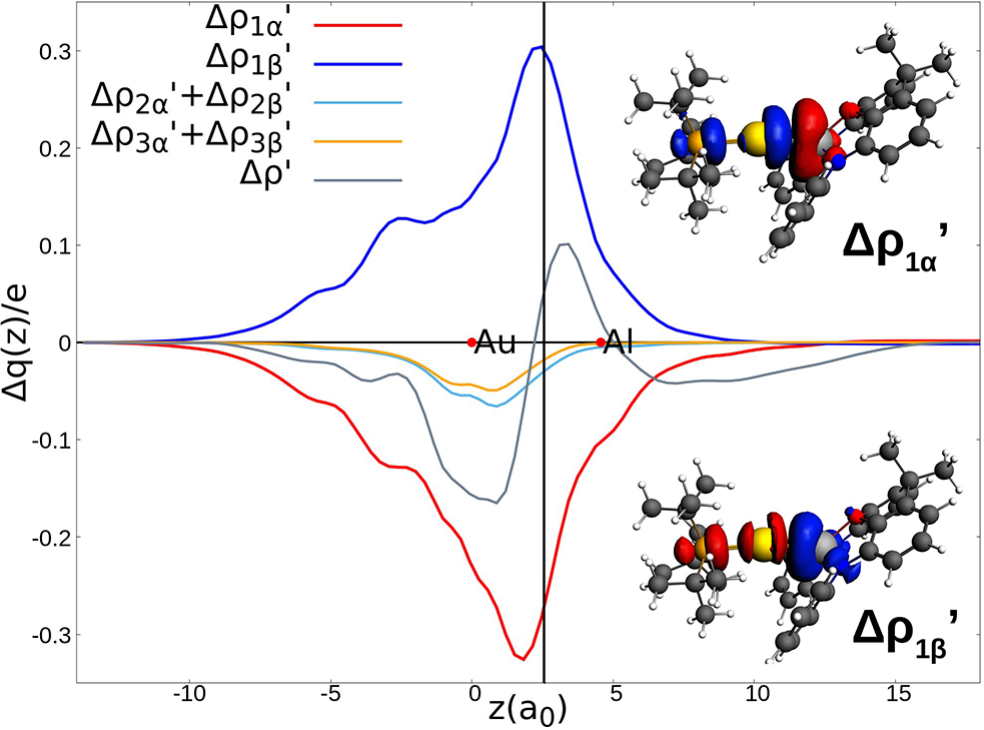
Charge Displacement (CD-NOCV) curves for the interaction between doublet [^t^Bu_3_PAu]· and [(NON’)Al]· fragments in the [^t^Bu_3_PAuAl(NON’)] complex. Red dots indicate the position of the nuclei along the *z* axis. The vertical solid line marks the isodensity boundary between the fragments. Positive (negative) values of the curve indicate right-to-left (left-to-right) charge transfer (see Supporting Information for details). Insets: isodensity surfaces (1 me/a_0_^3^) of the two NOCV deformation densities Δρ_1α_’ (top, right) and Δρ_1β_’ (bottom, right) (charge flux is red → blue).

The CD-NOCV curves clearly exhibit two similar main charge fluxes in opposite directions, which consist of an electron transfer from Au toward Al (Δρ_1α_’, red curve and inset in Figure 3) and from Al toward Au (Δρ_1β_’, blue curve and inset in Figure 3). These two charge fluxes have also similar CT absolute values (0.272 and 0.299 e for Δρ_1α_’ and Δρ_1β_’, respectively), associated orbital interaction energies (−32.66 and −24.49 kcal/mol for Δρ_1α_’ and Δρ_1β_’, respectively), and NOCV eigenvalues (0.45 and 0.42 for Δρ_1α_’ and Δρ_1β_’, respectively) as shown in Table S8. Other contributions to the Au–Al bond (CD-NOCV curves labeled as Δρ_2α_’ + Δρ_2β_’ and Δρ_3α_’ + Δρ_3β_’) describe the π back-donations (see Figure S7) and are definitely smaller in magnitude. The CD curve associated with the total deformation density (Δρ’) shows an almost symmetric charge accumulation at the bonding region with a slightly positive CT (0.05 e) going from the radical [(NON’)Al]· to [^t^Bu_3_PAu]·, which can be associated with the net polarization of the Au–Al bond. To definitely assess the electron-sharing bond nature, the CD-NOCV analysis for a nonpolar covalent bond system, such as the homonuclear Au_2_ molecule, is presented in Figures S8, S9 for comparison. The CD-NOCV curves in Figure 3 and Figure S8 are indeed very similar.

We also find that this bonding scheme is not peculiar of the Au–Al bond in [^t^Bu_3_PAuAl(NON’)]. A qualitatively analogous picture has been also obtained for two complexes with a Cu–Al bond, i.e., a model [^t^Bu_3_PCuAl(NON’)] (where we substituted gold with copper and reoptimized the structure) and the experimental complex **III**.^7^ (see Tables S9, S10 and Figures S10, S11; for a comparative EDA, see Table S11). Before concluding, we comment on the two main theoretical points which suggested in ref (1) the formation of a strongly polarized Au–Al bond with a large negative charge on Au, i.e., (i) the Au/Al difference in the *atomic* electronegativity values and (ii) the *atomic* charge on Au. Concerning point (i), although the large Au/Al atomic electronegativity difference (0.93 on the Pauling scale, 2.12 (calculated) and 2.19 (experimental) on the Mulliken scale (see Table S12) seems to be inconsistent with an electron-sharing bond, the calculated Mulliken “molecular electronegativity” is practically identical (2.56 vs 2.53 eV for [^t^Bu_3_PAu]· and [Al(NON’)]·, respectively) (Table S13). For point (ii), the atomic charges show a huge variability range with the chosen method (consistently with the highly directional and diffuse HOMO of the aluminyl anion, Figure S12): q^Au^, from −0.83 to +0.22; q^Al^, from 2.18 to 0.18 (Table S14) which makes an assessment of the bond polarization based on these numbers impossible.

In summary, the reactivity shown here points out that both Au and Al centers act as nucleophiles (radical-like mechanism), with the electrophilic behavior of Al also assisting the interaction with CO_2_. The Au–Al bonding picture in [^t^Bu_3_PAuAl(NON)] is consistent with an Au(0) involved in an electron-sharing bond-type. An important general conclusion is that the reactivity of metal-aluminyl complexes with CO_2_ resulting in M-CO_2_ coordination mode cannot be considered in itself as a probe for a strongly polarized M(δ-)-Al(δ+) bond and for the metal behaving as a standard nucleophilic center. We believe that the interpretative framework given here may be useful for future experimental investigations on CO_2_ capture and reduction by these unconventional bimetallic complexes.

## Supporting Information Available

Methodology and computational details; optimized geometries; Mayer’s bond order along the path; Activation Strain Model and EDA results; donor and acceptor NOCV orbital decomposition; analysis of the [(NON’)Al] and [^t^Bu_3_PAu] fragments; selection of fragmentations for bonding analysis; CD-NOCV analysis complete results; CD-NOCV and EDA analysis of [^t^Bu_3_PCuAl(NON’)] and [NHC^iPr^CuAlSiN^Dipp^] complexes; Au, Al, and “molecular” ionization energy and electron affinity; AIM, Mulliken, Hirshfeld, VDD, Multipole Derived, CM5 calculated atomic charges on Au and Al; [(NON’)Al] HOMO electron density map (PDF)

## Abbreviations

NON: 4,5-bis(2,6-diisopropylanilido)-2,7-di*tert*-butyl-9,9-dimethylxanthene
NHC^IPr^: *N*,*N*′-di-isopropyl-4,5-dimethyl-2-ylidene
^Me2^CAAC: 1-((2,6-di-isopropylphenyl)-3,3,5,5-tetramethyl-pyrrolidin-2-ylidene)
SiN^Dipp^: (CH_2_SiMe_2_NDipp)_2_
Ar*: C_6_H_2_{C(H)Ph_2_}_2_Me-2,6,4.
