# Supplementary material for: Reactivity of a Gold-Aluminyl Complex with Carbon Dioxide: A Nucleophilic Gold?
Source: J Am Chem Soc. 2021 Sep 2;143(36):14433–7. doi: 10.1021/jacs.1c06728 (PMC8447181; doi:10.1021/jacs.1c06728)
Supplement: Supplementary file 1 — ja1c06728_si_001.pdf [file ja1c06728_si_001.pdf]

Supporting Information

Reactivity of a Gold-Aluminyl Complex with Carbon Dioxide: A Nucleophilic Gold?

Diego Sorbelli[a]*, Leonardo Belpassi[bc]* and Paola Belanzoni[ab]*

[a] *Department of Chemistry, Biology and Biotechnology, University of Perugia, Via Elce di Sotto, 8 – 06123, Perugia, Italy*

[b] *Istituto CNR di Scienze e Tecnologie Chimiche "Giulio Natta" (CNR-SCITEC), Via Elce di Sotto, 8 – 06123, Perugia, Italy*

[c] *Computational Laboratory for Hybrid/Organic Photovoltaics (CLHYO) c/o Istituto CNR di Scienze e Tecnologie Chimiche "Giulio Natta" (CNR-SCITEC), Via Elce di Sotto, 8 – 06123, Perugia, Italy*

*Email: D.S. diegosorbellioo@gmail.com, L.B. leonardo.belpassi@cnr.it, P.B. paola.belanzoni@unipg.it

Table of Contents

Methodology…………………………………………………………………S2
Computational details………………………………………………………S6
Figure S1……………………………………………………………………S7
Tables S1-S2…………………………………………………………………S8
Figure S2……………………………………………………………………S9
Figure S3, ASM results ……………………………………………………S10
Figure S3……………………………………………………………………S13
Figure S4……………………………………………………………………S14
[^{t}Bu$_3$PAu] and [Al(NON')] fragment analysis……………………………S15
Fragmentation selection……………………………………………………S19
Figures S8-S9………………………………………………………………S22
Figure S10-Table S9 ………………………………………………………S25
Figure S11-Table S10………………………………………………………S26
Table S11……………………………………………………………………S27
Tables S12-S13………………………………………………………………S28
Table S14-Figure S12………………………………………………………S29
References……………………………………………………………………S30
Geometries xyz………………………………………………………………S34

Methodology

- **Natural Orbitals for Chemical Valence and Charge Displacement analysis**

The Natural Orbitals for Chemical Valence (NOCV)[1,2] is a suitable approach for the description of chemical bonding and is based on the rearrangement of the electron density occurring when a chemical bond is formed. In general, arrangement can be expressed as electron density difference between the formed adduct (AB) and sum of the densities of the two non-interacting fragments (A and B) frozen in their adduct geometry.

This deformation density can be brought into diagonal contributions in terms of NOCVs. In the NOCV scheme, the charge rearrangement taking place upon bond formation is obtained from the occupied orbitals of the two fragments suitably orthogonalized to each other and renormalized (*promolecule*). The resulting electron density rearrangement ($\Delta\rho'$) can be expressed in terms of NOCV pairs which are defined as the eigenfunctions of the so-called "valence operator"[3–5] as follows:

$$\Delta\rho' = \sum_k \nu_k(|\phi_{+\mathrm{k}}|^2 - |\phi_{-\mathrm{k}}|^2) = \sum_k \Delta\rho'_k \qquad [1]$$

where ϕ_{+k} and ϕ_{-k} are the NOCV pairs orbitals and $\nu_{\pm k}$ are the corresponding eigenvalues. When the adduct is formed from the promolecule, a fraction ν_k of electrons is transferred from the ϕ_{-k} to the ϕ_{-k} orbital, which are envisaged as donor and acceptor orbitals, respectively. For the sake of interpretation, a population analysis can also be performed in order to single out, for ϕ_{-k} and ϕ_{+k} orbitals, which molecular orbitals (MOs) of the two constituting fragments contribute to the interaction (with a resulting associated coefficient accounting for the magnitude of the contribution).

The NOCV scheme can be coupled with the framework of the Charge Displacement (CD) analysis. The CD analysis allows to quantify the amount of electronic charge that is transferred between the two fragments upon the formation of the A-B bond. The Charge Displacement function (Δq) is defined as the partial progressive integration on a suitable z-axis of the deformation density $\Delta\rho'$:[6]

$$\Delta q(z) = \int_{-\infty}^{z} dz' \int_{-\infty}^{+\infty} \int_{-\infty}^{+\infty} \Delta \rho'(x,y,z') dxdy \quad [2]$$

The CD function, Δq(z), quantifies at each point of the bond axis the exact amount of electron charge that, upon formation of the bond, is transferred from the right to the left across a plane perpendicular to the bond axis through z.

The CD and NOCV frameworks can be coupled in the CD-NOCV scheme.[7] In the latter, the density rearrangement due to the bond formation between two fragments, ($\Delta\rho'$), can be partitioned in different NOCV deformation densities ($\Delta\rho'_k$) and therefore one is able to quantify the charge transfer (CT) associated to each different component. It must be noted that only few of the NOCV pairs contributes to the chemical bond. Therefore, when the CD-NOCV analysis is carried out, usually only the first $\Delta\rho_k'$ components are investigated in order to understand which significant chemical contribution to the bond they represent.

In equation [2], the integration axis is usually conveniently chosen as the bond axis between the two fragments constituting the adduct and usually we choose to evaluate the charge transfer between A and B by taking the CD value at the “isodensity boundary”, i.e. the z-point where equally valued isodensity surfaces of the isolated fragments become tangent.[6,8]

In this case, since we also apply this scheme to the transition state TSI, with [^{t}Bu$_3$PAuAl(NON’)] and [CO_2] as fragments, such approach is complicated, since the two fragments display multiple interactions with multiple atomic centers and thus it is clearly impossible to define a unique bond axis and it is very hard to rely on the isodensity boundary for the estimation of the charge transfer. In order to avoid any ambiguity in the definition of the z-axis, we recall an approach that may be useful for evaluating the charge transferred between the [^{t}Bu$_3$PAuAl(NON’)] and [CO_2] fragments at TSI.[9]

Within this approach, the electron density rearrangement ($\Delta\rho'$), which typically shows charge accumulation regions (positive values) and charge depletion regions (negative values), defines two different positive functions, $\Delta\rho^+$ and $\Delta\rho^-$, each equal to the magnitude of the appropriate portion, *i.e.*:

$$\Delta\rho^{+/-}(r) = max[\pm\Delta\rho(r)', 0] \quad [3]$$

so that

$$\Delta\rho(r)' = \Delta\rho^{+}(r) - \Delta\rho^{-}(r) \quad [4]$$

By defining two arbitrary regions that are associated with the interacting fragments, we can evaluate the charge transfer as follows:

$$CT = \int_A \Delta\,\rho(r)'dr = -\int_B \Delta\,\rho(r)'dr \quad [5]$$

By combining Eqs. [4] and [5], CT can also be expressed as:

$$CT = \int_A \Delta\,\rho^{+}(r)dr - \int_A \Delta\,\rho^{-}(r)dr = -\int_B \Delta\,\rho^{+}(r)dr + \int_B \Delta\,\rho^{-}(r) \quad [6]$$

Ultimately, this approach can also be expressed in the CD-NOCV framework. By combining Equations [1] and [5], we can use to this approach for calculating the charge transfer associated to each NOCV deformation density as follows:

$$CT_k = \int_A \Delta\,\rho_k(r)'dr = -\int_B \Delta\,\rho_k(r)'dr \quad [7]$$

Despite the spatial regions associated to the two interacting fragments being defined arbitrarily, this approach is particularly suitable for the analysis of the interaction between the [^{t}Bu$_3$PAuAl(NON')] and [CO_2] fragments in TSI, being the two fragments well-separated in space.

- **Energy Decomposition Analysis and ETS-NOCV approach**

In this work the Energy Decomposition Analysis (EDA)[10] has been applied to get additional and complementary insights into the interaction between carbon dioxide and the [^{t}Bu$_3$PAuAl(NON')] complex in the transition state TSI. With this approach, the interaction energy between the [^{t}Bu$_3$PAuAl(NON')] and [CO_2] fragments can be decomposed in different contributions as follows:

$$\Delta E_{\text{int}} = \Delta E^{Pauli} + \Delta V_{elst} + \Delta E_{oi} + \Delta E_{disp} \quad [8]$$

where ΔE^{Pauli} represents the Pauli repulsion interaction between occupied orbitals on the two fragments, ΔV_{elst} is the quasiclassical electrostatic interaction between the unperturbed charge

distribution of the fragments at their final positions, ΔE_{disp} takes into account the dispersion contribution and ΔE_{oi} is the orbital interaction, which arises from the orbital relaxation and the orbital mixing between the fragments, and accounts for electron pair bonding, charge transfer, and polarization.

The orbital interaction term ΔE_{oi} can be further decomposed within the ETS-NOCV[11] scheme into NOCV pairwise orbital contributions ($\Delta E_{oi} = \sum_k \Delta\, E_{oi}^k$) which associates an energy contribution (E_{oi}^k) to each NOCV deformation density ($\Delta\rho_k$).

- **Activation Strain Model**

The Activation Strain Model (ASM, which is also referred to as "distortion/interaction analysis")[12–14] is a popular approach often used in order to get insights into the factors controlling the activation barrier of a process. Within this framework, the activation barrier ($\Delta E^{\#}$) can be decomposed as follows:

$$\Delta E^{\#} = \left[\Delta E_{\text{dist}}^{TSI} - \Delta E_{dist}^{RC}\right] + \left[\Delta E_{\text{int}}^{TSI} - \Delta E_{\text{int}}^{RC}\right] = \Delta\Delta E_{dist} + \Delta\Delta E_{\text{int}} \qquad [9]$$

where the "ΔE_{dist}^{TSI}" and "ΔE_{dist}^{RC}" terms represent the energy penalty due to the distortion of the fragments (i.e. [^{t}Bu$_3$PAuAl(NON')] and CO_2 in the case of the barrier involving the transition state TSI) constrained in the structures of the transition state (TSI) and the reactant complex (RC) respectively, whereas "ΔE_{int}^{TSI}" and "ΔE_{int}^{RC}" represent the interaction energies between the fragments (with the geometries constrained at the ones assumed in the TSI and RC, respectively) in the two structures. These terms can be grouped in the "$\Delta\Delta E_{dist}$" and "$\Delta\Delta E_{int}$" terms, that represent the overall distortion and interaction contributions to the activation barrier, respectively.

Additionally, we can also rearrange Equation [9] in order to express the distortion contributions relatively to the two fragments as follows:

$$\begin{aligned} \Delta\Delta E_{\text{dist}} &= E_{CO2}^{TS} - E_{CO2}^{RC} + E_{AuAl}^{TS} - E_{AuAl}^{RC} \\ \Delta\Delta E_{\text{dist}} &= \Delta E_{\text{dist}}^{CO2} + \Delta E_{\text{dist}}^{AuAl} \end{aligned} \qquad [10]$$

where "ΔE_{dist}^{CO2}" represent the distortion penalty (or stabilization) due to CO_2 rearranging from its

structure in the RC going into TSI and the "ΔE_{dist}^{AuAl}" term represents the same distortion contribution concerning the rearrangement of the [$^{t}Bu_3PAuAl$(NON')] complex.

Computational details

Complex **I** has been slightly simplified at the NON site by replacing the two tert-butyl groups at the peripheral positions of the dimethylxanthene moiety with hydrogen atoms and the two Dipp substituents on the nitrogen atoms with phenyl groups (denoted as NON'). Complex **I**, **INT** and **PC** structures have been also optimized with the real NON ligand for assessing the effect of the model on the reaction energetics.

All geometry optimizations and frequency calculations on optimized structures (minima with zero imaginary frequencies and transition states with one imaginary frequency) for the CO_2 insertion into the [$^{t}Bu_3PAuAl$(NON')] complex reaction have been carried out using the Amsterdam Density Functional (ADF) code[15,16] in combination with the related Quantum-regions Interconnected by Local Description (QUILD) program.[17] The PBE[18] GGA exchange-correlation (XC) functional, the TZ2P basis set with a small frozen core approximation for all atoms, the ZORA Hamiltonian[19–21] for treating scalar relativistic effects and the Grimme's D3-BJ dispersion correction were used.[22,23] Solvent effects were modeled employing the Conductor-like Screening Model (COSMO) with the default parameters for toluene as implemented in the QUILD code.[24] The same computational setup has also been used for the calculation of the Coulomb potential maps, for the calculation of atomic and molecular ionization energies and electron affinities and for carrying out the EDA and CD-NOCV calculations. This computational protocol has been used in ref. [25] to study the [$^{t}Bu_3PAuAl$(NON)] and [$^{t}Bu_3PAuCO_2Al$(NON)] complexes.

The atomic charges were calculated using different schemes as implemented in the ADF code, that is: Voronoi Deformation Density (VDD)[26], Mulliken charges[27], Hirshfeld charges[28], Multipole Derived Charge Analysis[29], the Charge Model Five (CM5) analysis[30] and Bader's Atoms in Molecules (AIM)[31,32] analysis. All charges have been calculated at the PBE-D3 level using a large QZ4P basis set. Mayer's bond orders[33] have been calculated by carrying out single-point calculations using the software ORCA,[34] with the PBE functional, Ahlrich's def2-QZVPP basis set,[35] Grimme's D3-BJ dispersion correction and the def2 19-VE pseudopotential for the inclusion of scalar relativistic effects.

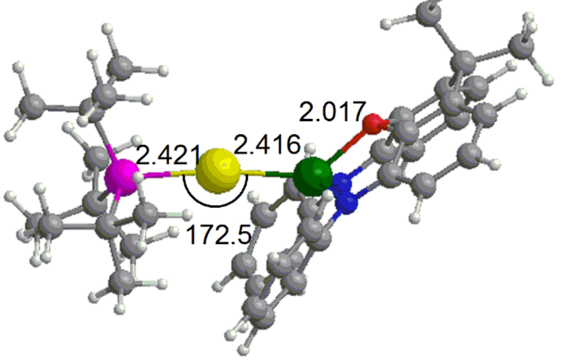

$^{t}But_3PAuAl(NON')$

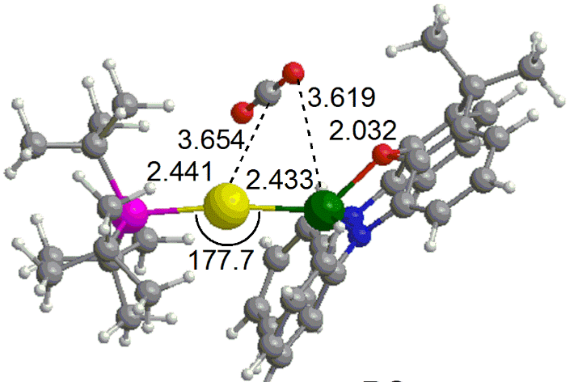

RC

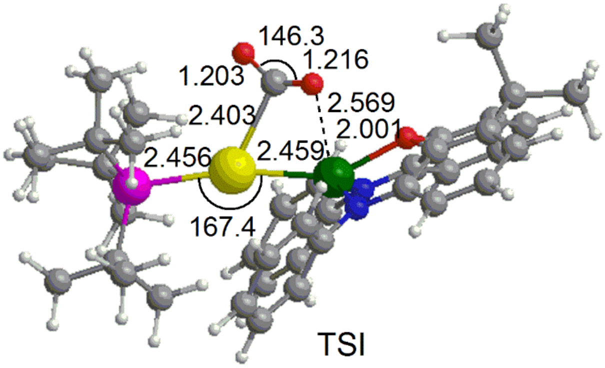

TSI

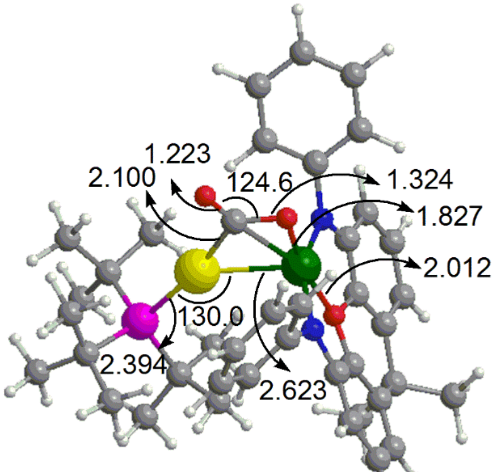

INT

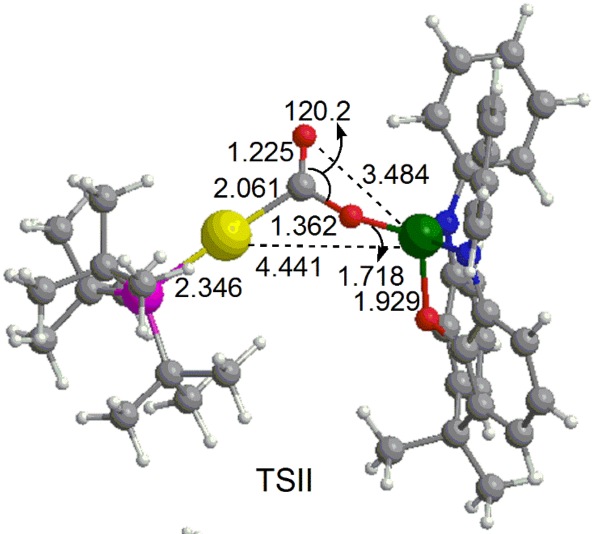

TSII

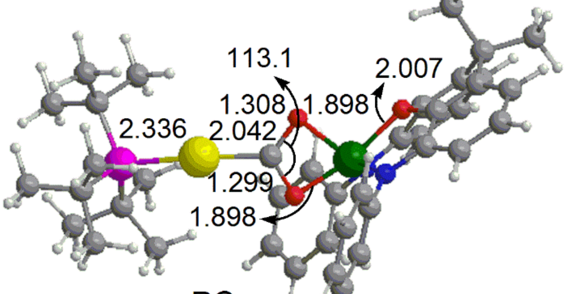

PC

Figure S1. Optimized structures of [$^{t}Bu_3PAuAl(NON')$], RC, TSI, INT, TSII and PC complexes. Main geometrical parameters are reported (bond in Å, angles in degree).

The TSI possesses one imaginary frequency (-147.1 cm^{-1}) which is associated to a complex vibrational motion involving the two metal atoms and the oxygen and carbon atoms of CO_2. A concerted transition state involving the Au-CO_2 and Al-CO_2 interactions is clearly suggested. The imaginary frequency of TSII is instead very small (-14.2 cm^{-1}), indicating a very flat potential energy surface. The mode associated to this frequency involves bending of the two [$^{t}But_3PAu$] and [Al(NON')] fragments between which the CO_2 insertion occurs.

	INT	PC
NON'	-17.4	-27.4
NON	-15.8	-28.6

Table S1. Comparison between ΔE values (in kcal/mol) calculated for INT e PC using a simplified NON ligand (NON') and the full NON ligand (NON). The ΔE values refer to I + CO_2 taken as the zero energy reference.

	[$^{t}Bu_3PAuAl(NON')$]	Exp	[$^{t}Bu_3PAuCO_2Al(NON')$]	Exp
Al-Au	2.416 (2.427)	2.402		
Au-P	2.421 (2.430)	2.395	2.336 (2.334)	2.300
Al-N1	1.927 (1.923)	1.900	1.892 (1.889)	1.860
Al-N2	1.931 (1.925)	1.904	1.902 (1.902)	1.887
Al-O	2.017 (2.071)	2.046	2.007 (2.015)	1.990
Al-Au-P	172.5 (169.2)	167.5		
Au-C			2.042 (2.040)	2.042
Al-O1			1.898 (1.902)	1.880
Al-O2			1.898 (1.891)	1.861
C-Au-P			171.8 (173.5)	176.5
O-C-O			113.1 (113.5)	113.2

Table S2. Main geometrical parameters of the optimized [^{t}Bu$_3$PAuAl(NON')] and [^{t}Bu$_3$PAuCO$_2$Al(NON')] structures. Values in parenthesis refer to the optimized [^{t}Bu$_3$PAuAl(NON)] and [^{t}Bu$_3$PAuCO$_2$Al(NON)] structures (with the full NON ligand). Available experimental crystal data taken from Ref. 25 are reported for comparison. Distances are in Å, angles in degrees.

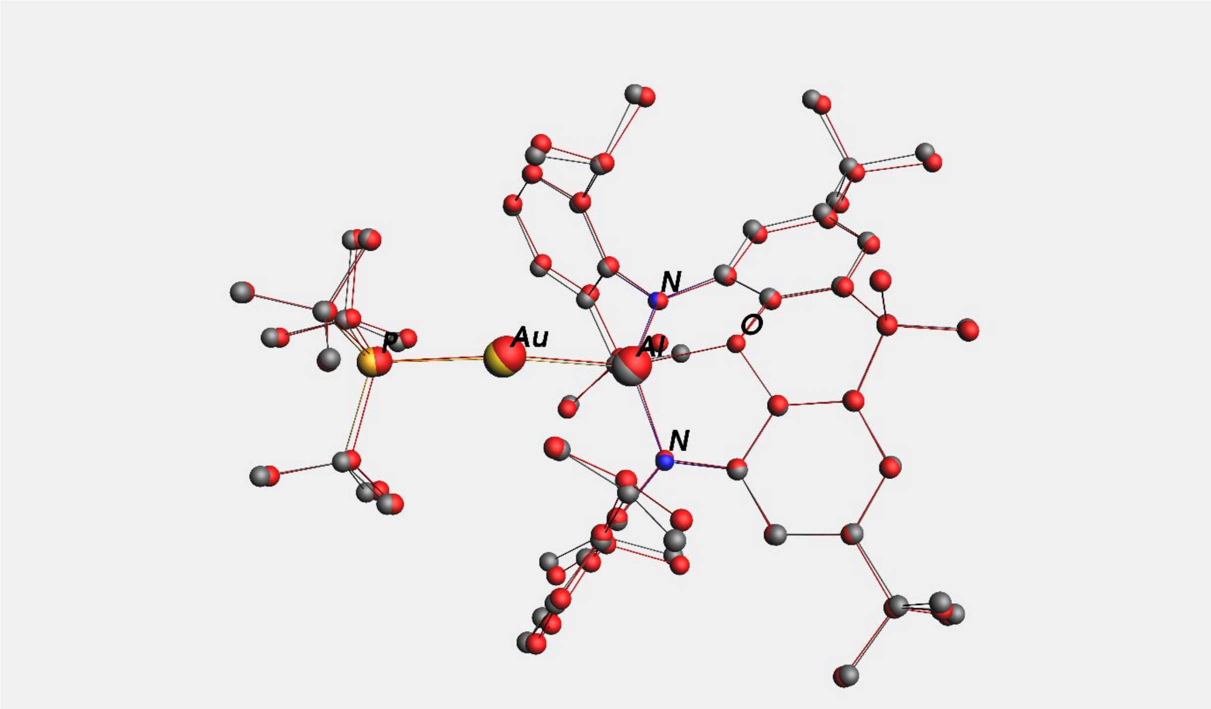

Figure S2. Superposition of the [^{t}Bu$_3$PAuAl(NON)] complex I structures: red) experimental crystal structure; grey) optimized structure (PBE-D3 + solvent level). Hydrogen atoms are omitted for clarity.

Figure S2 shows that the [^{t}Bu$_3$PAuAl(NON)] complex I geometry optimized at the PBE-D3 + solvent level agrees well with the experimental crystal structure.

	RC	TSI	INT	TSII	PC
**Al-Au**	0.81	0.81	0.42	/	/
**Al-O^1_{CO2}**	/	0.14	0.79	1.08	0.67
**Al-O^2_{CO2}**	/	/	/	/	0.58
**Al-C_{CO2}**	/	0.16	0.19	/	/
**Au-O^2_{CO2}**	/	0.13	0.11	/	/
**Au-C_{CO2}**	/	0.26	0.52	0.53	0.45
**C_{CO2}-O^1_{CO2}**	2.13	1.80	1.25	1.05	1.33
**C_{CO2}-O^2_{CO2}**	2.12	1.95	1.96	1.97	1.36

Table S3. Mayer's bond orders along the reaction path for the most relevant interactions. Only bond orders greater than 0.1 are reported.

ASM results

**$\Delta E^{\#}$**	8.94
**ΔE_{INT}^{RC}**	-5.12
**ΔE_{INT}^{TS}**	-18.00
**$\Delta\Delta E_{INT}$**	-12.88
**ΔE_{DIST}^{RC}**	0.47
**ΔE_{DIST}^{TS}**	22.29
**ΔE_{DIST}^{CO2}**	20.32
**ΔE_{DIST}^{AuAl}**	1.50
**$\Delta\Delta E_{DIST}$**	21.82

Table S4. Results of the Activation Strain Model (ASM) analysis of the [CO_2]-[$^{t}Bu_3PAuAl$(NON')] interaction for the first electronic energy activation barrier involving transition state TSI. Energies are given in kcal/mol.

The electronic energy activation barrier ($\Delta E^{\#}$=8.94 kcal/mol) is decomposed into two contributions: the distortion component ($\Delta\Delta E_{dist}$), which arises from deforming the two fragments ([CO_2] and [$^tBu_3$PAuAl(NON')]) from their geometry in RC to the geometry they acquire in the TSI, and the stabilizing interaction component ($\Delta\Delta E_{int}$), which is associated to the different energy interaction between the two fragments in the RC and TSI structures. The ASM formalism is briefly summarized in the Methodology section and all the results are re-ported in Table S4. The distortion energy ($\Delta\Delta E_{dist}$=21.82 kcal/mol) is also decomposed into contributions arising from the two separated fragments. This decomposition reveals that most of the distortion penalty (20.32 kcal/mol) is associated with the bending of CO_2, while the distortion energy associated with the [$^tBu_3$PAuAl(NON')] fragment is very small (1.50 kcal/mol). This points out that the remarkable geometrical rearrangement of the N,O-donor chelating NON ligand (particularly at O-site) observed along the reaction energy path (see Table S5 and discussion) is nearly cost-less. The interaction stabilization ($\Delta\Delta E_{int}$= -12.88 kcal/mol) is able to efficiently counterbalance the distortion penalty. The orbital interaction energy at TSI (-53.30 kcal/mol) is the key contribution to it (see Table S6).

Parameter	RC	TSI
r_{O-Al}	2.006 Å	2.001 Å
r_{N1-Al}	1.926 Å	1.901 Å
r_{N2-Al}	1.930 Å	1.916 Å
r_{Au-Al}	2.416 Å	2.459 Å
r_{Au-P}	2.422 Å	2.456 Å
$\alpha_{N1-Al-N2}$	125.8°	129.5°
$\alpha_{O-Al-Au}$	133.7°	148.0°
α_{H-Au-P}	58.3°	57.1°
$\alpha_{Al-Au-P}$	172.3°	167.4°
$\delta_{Au-Al-N-O}$	133.9°	148.3°

Table S5. Relevant geometrical parameters of the [$^tBu_3$PAuAl(NON')] fragment constrained at the geometry it acquires in RC and TSI.

The results reported in Table S5 show that, upon going from RC to TSI, the [^{t}Bu$_3$PAu] moiety in the [^{t}Bu$_3$PAuAl(NON')] fragment does not undergo significant geometrical rearrangements, with the H-Au-P angle ($\alpha_{H\text{-}Au\text{-}P}$, which can be roughly assumed as an estimate of a third of the Tolman's cone angle for the phosphine ligand) remaining practically unvaried (58.3° vs 57.1°) and the Au-P distance ($r_{Au\text{-}P}$) and the Al-Au-P angle ($\alpha_{Al\text{-}Au\text{-}P}$) not changing dramatically (2.422 Å vs. 2.456 Å and 172.3° and 167.4°).

On the other hand, substantial rearrangements are observed concerning the NON' ligand orientation with respect to the Au-Al bond, with the O-Al-Au angle ($\alpha_{O\text{-}Al\text{-}Au}$) changing by almost 20° (133.7° vs. 148.0°), as well as the Au-Al-N-O dihedral angle ($\delta_{Au\text{-}Al\text{-}N\text{-}O}$, 133.9° vs 148.3°). It is noteworthy that also the Al-N distances are sensibly shortened on going from RC to TSI ($r_{Al\text{-}N1}$ changes from 1.926 to 1.901 Å and $r_{Al\text{-}N2}$ varies from 1.930 to 1.916 Å).

**$\Delta\Delta E_{INT}$**	-12.88
**$\Delta\Delta E_{INT}^{Pauli}$**	99.48
**$\Delta\Delta E_{INT}^{elst}$**	-58.04
**$\Delta\Delta E_{INT}^{steric}$**	41.44
**$\Delta\Delta E_{INT}^{oi}$**	-53.30
**$\Delta\Delta E_{INT}^{disp}$**	-0.75
**$\Delta\Delta E_{INT}^{solvation}$**	-0.26

Table S6. Energy Decomposition Analysis (EDA) of the interaction stabilization ($\Delta\Delta E_{int}$) derived from the Activation Strain Model (ASM) analysis of the [CO_2]-[^{t}Bu$_3$PAuAl(NON')] interaction for the first electronic energy activation barrier involving transition state TSI. Energies are given in kcal/mol.

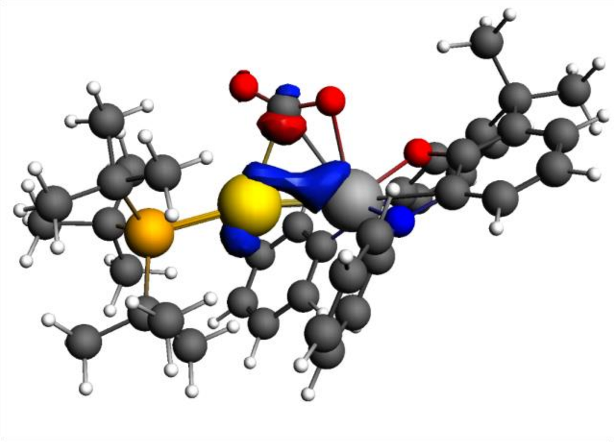

Donor orbital Ψ_1

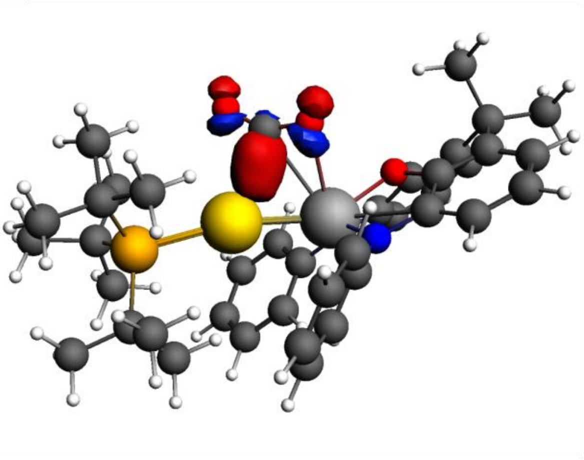

Acceptor orbital Ψ_{-1}

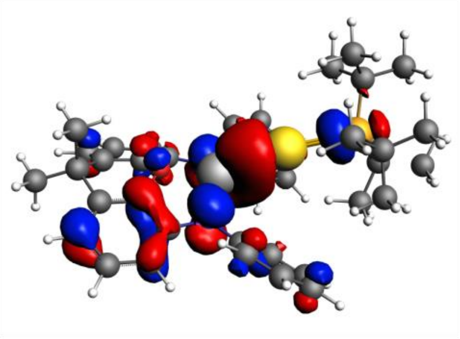

HOMO
[^{t}But$_3$PAuAl(NON')]
(-0.104)

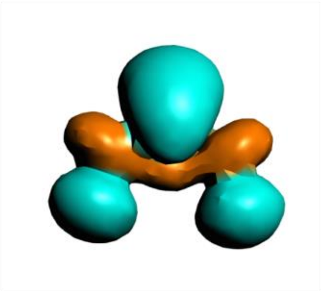

LUMO CO_2
(0.452)

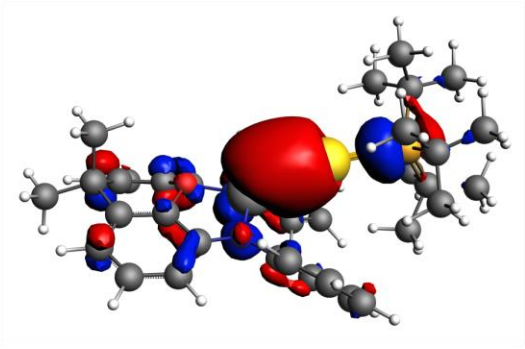

HOMO-2
[^{t}But$_3$PAuAl(NON')]
(-0.181)

Figure S3. Breakdown of the donor (Ψ_1) and acceptor (Ψ_{-1}) orbitals that are associated with the deformation density $\Delta\rho'_1$ in the transition state TSI into the most important MOs of the fragments frozen at their TSI geometry. The orbital mixing coefficients are given in parentheses.

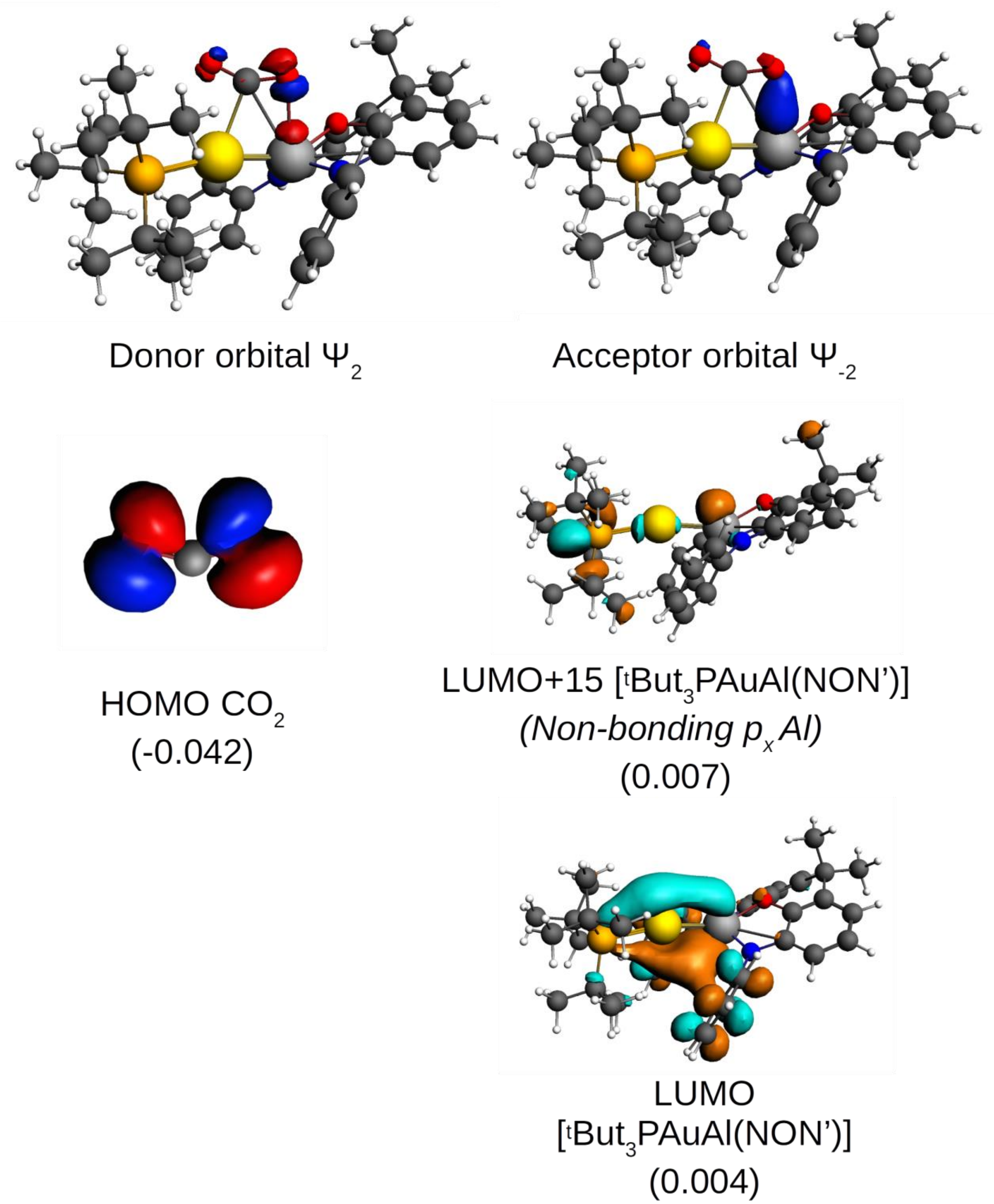

Figure S4. Breakdown of the donor (Ψ_2) and acceptor (Ψ_{-2}) orbitals that are associated with the deformation density $\Delta\rho'_2$ in the transition state TSI into the most important MOs of the fragments frozen at their TSI geometry. The orbital mixing coefficients are given in parentheses.

In Figure S4, among the many MOs contributing to the [^{t}Bu$_3$PAuAl(NON')] acceptor orbital, two MOs are shown: a high-lying MO representing the non-bonding 3p_x orbital of Al (LUMO + 15) and the LUMO of the [^{t}Bu$_3$PAuAl(NON')] fragment, which can be described as a π bonding combination between the Au and Al p_x orbitals, with contribution also from the P p_x orbital.

Structural and energy analysis of the [^{t}Bu$_3$PAu] and [Al(NON')] fragments and their reactivity with CO_2

Since the Au-Al bond in the [^{t}Bu$_3$PAuAl(NON)] complex has been described as homolitic in Ref. 25, an analysis of the separated functionalities [^{t}Bu$_3$PAu] and [Al(NON')] may be useful to shed light on its reactivity with carbon dioxide.

Firstly, the electronic energies of the optimized structures of cationic, anionic and neutral radical [^{t}Bu$_3$PAu] and [Al(NON')] fragments are used to calculate the bonding energy for the [^{t}Bu$_3$PAuAl(NON')] complex to get insights into the Au-Al bond nature. It is interesting to note that the most favorable charge distribution in the dissociation products (i.e. the least negative bonding energy) for the [^{t}Bu$_3$PAuAl(NON')] complex is a situation where the Au-Al bond dissociates homolitically:

[^{t}Bu$_3$PAu]• + [(NON')Al]• → [^{t}Bu$_3$PAuAl(NON')] ΔE = -82.6 kcal/mol (1)

[^{t}Bu$_3$PAu]$^+$ + [(NON')Al]$^-$ → [^{t}Bu$_3$PAuAl(NON')] ΔE = -119.9 kcal/mol (2)

[^{t}Bu$_3$PAu]$^-$ + [(NON')Al]$^+$ → [^{t}Bu$_3$PAuAl(NON')] ΔE = -144.4 kcal/mol (3)

This result suggests that the bonding interaction should be considered as a conventional covalent bond instead of a coordination or dative bond. Bonding energy for reaction (1) has been also calculated from neutral radical fragments at the same geometry they have in the [^{t}Bu$_3$PAuAl(NON')] complex. Remarkably, a very close value has been found (ΔE = -83.8 kcal/mol vs. -82.6 kcal/mol), with the same spin density distribution within the corresponding [^{t}Bu$_3$PAu]• and [(NON')Al]• fragments. The Au-Al homolitic bond in the [^{t}Bu$_3$PAuAl(NON')] complex is thus fully consistent with the neutral radical fragments picture.

For the [$^{t}Bu_3PAuCO_2Al(NON')$] PC complex, different fragmentations can be considered for describing the CO_2 bond to the [$^{t}Bu_3PAu$] and/or the [(NON')Al] fragments. Remarkably, the most favorable charge distribution in the dissociation products (i.e. the least negative bonding energy) is analogously a situation where the Au-CO_2 and Al-CO_2 bond dissociate homolitically, with the lowest bonding energy calculated for reaction (4):

[$^{t}Bu_3PAu$]• + [$CO_2Al(NON')$]• → [$^{t}Bu_3PAuCO_2Al(NON')$] ΔE = -92.1 kcal/mol (4)

[$^{t}Bu_3PAuCO_2$]• + [(NON')Al]• → [$^{t}Bu_3PAuCO_2Al(NON')$] ΔE = -99.5 kcal/mol (5)

[$^{t}Bu_3PAu]^{+}$ + [$CO_2Al(NON')]^{-}$ → [$^{t}Bu_3PAuCO_2Al(NON')$] ΔE = -106.9 kcal/mol (6)

[$^{t}Bu_3PAuCO_2]^{+}$ + [$(NON')Al]^{-}$ → [$^{t}Bu_3PAuCO_2Al(NON')$] ΔE = -138.1 kcal/mol (7)

[$^{t}Bu_3PAu]^{-}$ + [$CO_2Al(NON')]^{+}$ → [$^{t}Bu_3PAuCO_2Al(NON')$] ΔE = -152.0kcal/mol (8)

[$^{t}Bu_3PAuCO_2]^{-}$ + [$(NON')Al]^{+}$ → [$^{t}Bu_3PAuCO_2Al(NON')$] ΔE = -143.5kcal/mol (9)

This finding indicates that also the bonding interaction between the [$^{t}Bu_3PAu$] and the [$CO_2Al(NON')$] fragments should be considered as a conventional covalent bond between Au and carbon dioxide C atoms. Further indication is derived from calculation of the bonding energy for reaction (4) from neutral radical fragments at the same geometry they have in the [$^{t}Bu_3PAuCO_2Al(NON')$] complex. Strikingly, a close value has been found (ΔE = -97.3 kcal/mol vs. -92.1 kcal/mol).

Such evidence is confirmed by the electron density rearrangement occurring upon formation of the Au-Al bond starting from open-shell radical fragments (Figure 3 in the text), where electron density is transferred in the region between the two metals, thus matching well with the previously depicted picture of the Au-Al bond behaving as a nucleophile.

In Figure S5 and S6 a direct comparison between TSI, INT, TSII and PC structures (singlet ground state) and separated corresponding neutral radical fragments optimized structures (doublet ground state) is presented. The similarity between each stationary state and its separated neutral radical fragments is indeed remarkable.

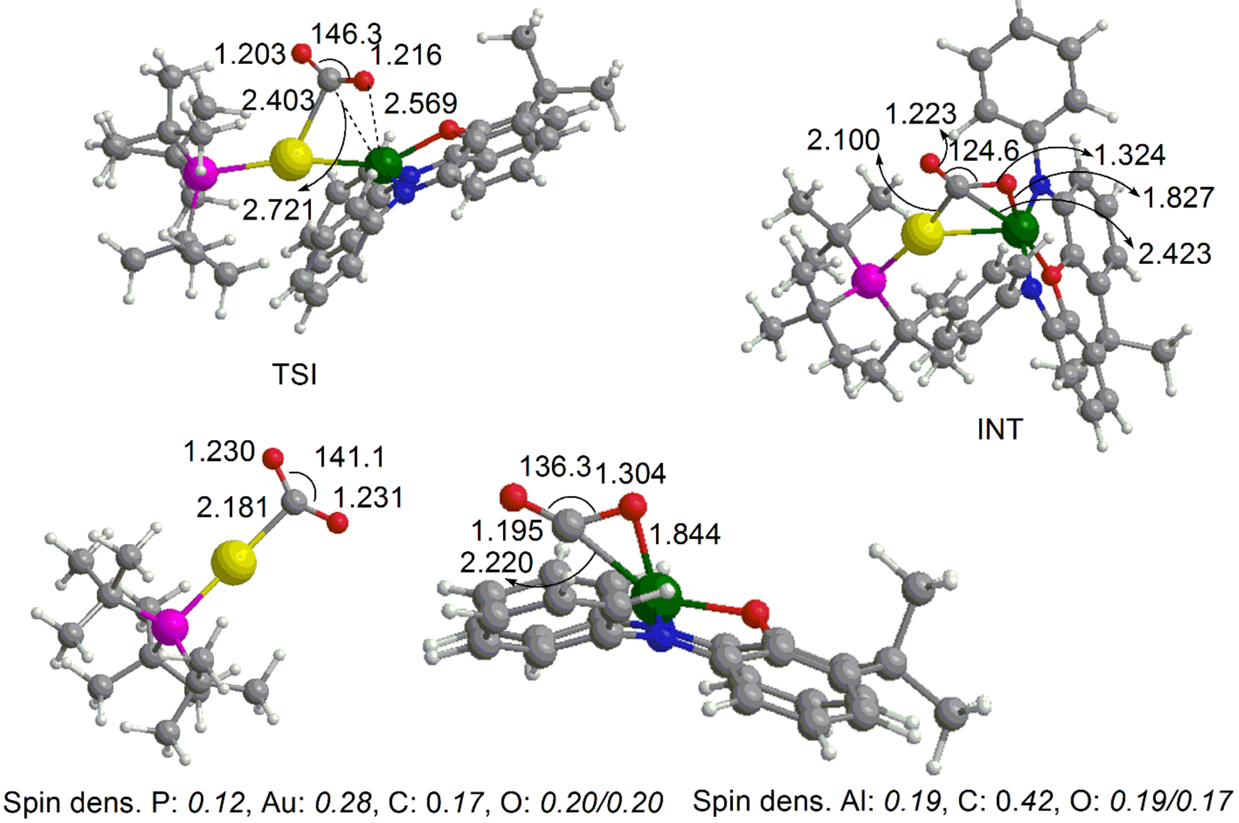

Figure S5. Optimized structures of TSI, INT (singlet ground state), neutral radical fragments, [$^{t}Bu_3PAuCO_2$]• and [$CO_2Al(NON)$]•(doublet ground state). Main geometrical parameters (distances in Å, angle in degrees) and spin density at atomic centers are reported.

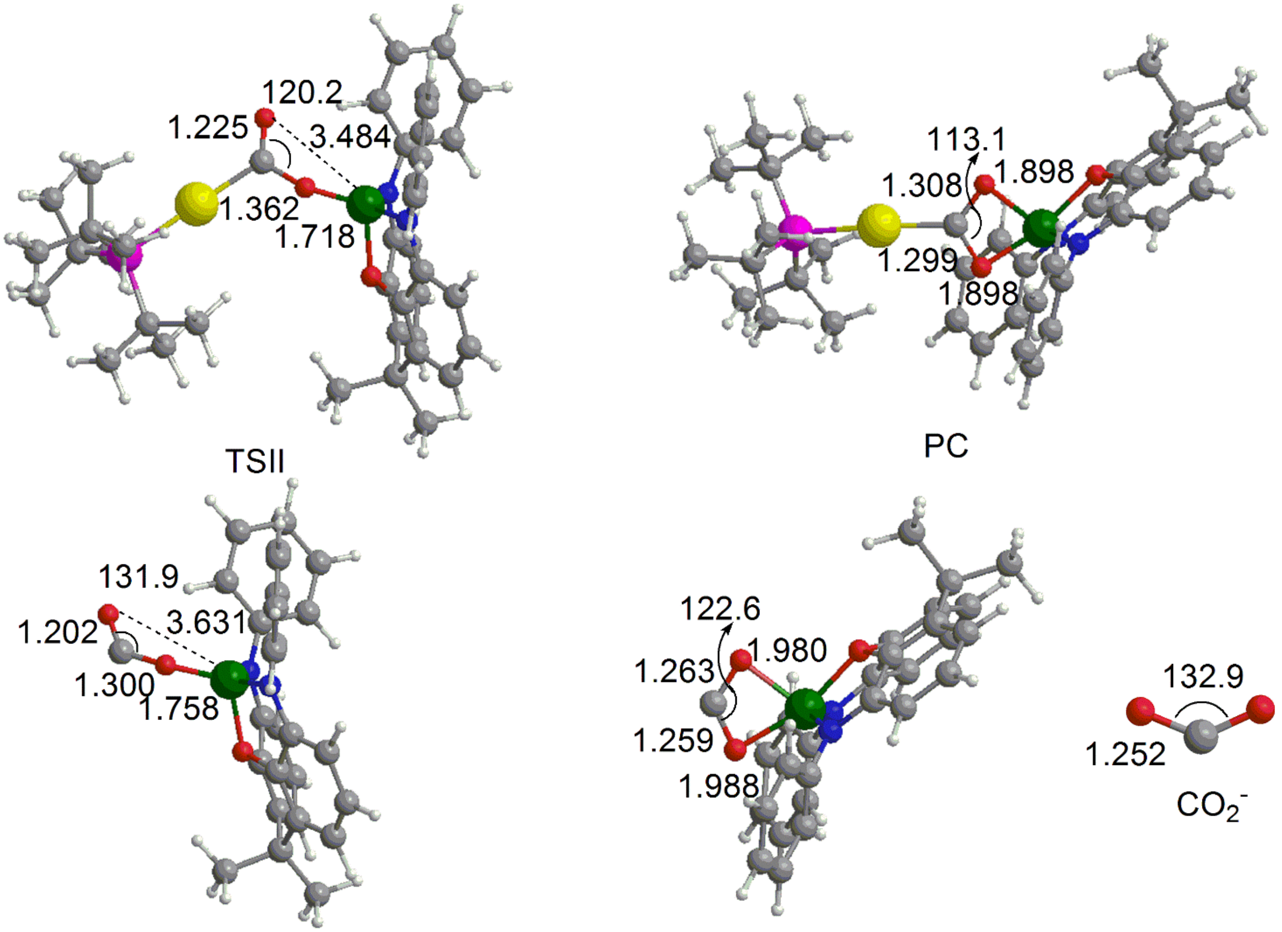

Figure S6. Optimized structures of TSII, PC (singlet ground state), two local minima of the neutral radical fragment [CO_2Al(NON)]• with the CO_2 coordination mode resembling that in TSII and PC, respectively, and CO_2^- (doublet ground state). Main geometrical parameters are reported (distances in Å, angle in degrees).

Notably, this geometrical analogy holds true also for the inserted CO_2 in PC and in $[CO_2Al(NON)]^{\bullet}$ fragment, which closely resembles the geometry of the free radical anion CO_2^{-}. We should mention that the electron attachment to CO_2 is energetically unfavorable,[36] although the radical anion CO_2^{-} has been experimentally characterized[37] and its controlled production for radical chain reactions has been very recently reported.[38]

Surprisingly, this simple model accounts for a possible radical-like mechanism for the CO_2 insertion into the Au-Al bond in the $[{}^{t}Bu_3PAuAl(NON')]$ complex. The stationary points in Figure 1 in the text have been also optimized at open-shell (unrestricted) singlet level, attaining the same geometries and energies as those calculated at closed-shell (restricted) level. This finding is consistent with the experimental evidence that the aluminyl anion $K_2[Al(NON)]_2$ reacts rapidly with 1 atm of CO_2 at room temperature to generate the carbonate and CO species. [39]□

Selection of the fragments for the Au-Al bonding analysis in $[{}^{t}Bu_3PAuAl(NON')]$

The choice of how to fragment a bond may be critical and in some cases subject to a certain arbitrariness. Our choice here is based on the results of the Energy Decomposition Analysis (EDA)[10]□ using all the possible fragmentations (i.e. singlet ground state charged fragments, $[{}^{t}Bu_3PAu]^{+/-}$ and $[(NON')Al]^{-/+}$, and doublet ground state neutral fragments) (Table S7). The radical fragments represent the lowest pathway for the breakage of the Au-Al bond which is indeed homolytic (dissociation into radicals: -87.56 kcal/mol, other fragmentations: -181.14 and -225.56 kcal/mol), and their associated orbital interaction (ΔE_{oi}) value is the smallest (radical fragments: -71.51 kcal/mol; closed shell fragments: -105.34 and -171.09 kcal/mol). These findings strongly indicate the open-shell radical fragments $[{}^{t}Bu_3PAu]\cdot$ and $[(NON')Al]\cdot$ as the best choice for describing the bond.[40,41]□

	[^{t}Bu$_3$PAu]· - [Al(NON')]·	[^{t}Bu$_3$PAu]$^+$- [Al(NON')]$^-$	[^{t}Bu$_3$PAu]$^-$-[Al(NON')]$^+$
**ΔE_{Pauli}**	167.80	218.09	200.21
**ΔE_{Elst}**	-173.43	-283.48	-244.25
**ΔE_{Steric}**	-5.63	-65.39	-44.05
**ΔE_{oi}**	-71.51	-105.34	-171.09
**ΔE_{disp}**	-10.42	-10.42	-10.42
**ΔE**	-87.56	-181.14	-225.56

Table S7. Energy Decomposition Analysis (EDA) of the interaction energy between [^{t}Bu$_3$PAu] and [Al(NON')] fragments in the [^{t}Bu$_3$PAuAl(NON')] complex using different fragmentations, i.e. doublet open shell ground state neutral fragments (first column), [^{t}Bu$_3$PAu]$^+$ singlet and [Al(NON')]$^-$ singlet ground state fragments (second column) and [^{t}Bu$_3$PAu]$^-$ singlet and [Al(NON')]$^+$ singlet ground state fragments (third column). Energies are reported in kcal/mol.

	**A**			**B**		
**K**	**$\lvert \nu_k \rvert$**	**ΔE_{oi}^k (kcal/mol)**	**CT^k (e)**	**$\lvert \nu_k \rvert$**	**ΔE_{oi}^k (kcal/mol)**	**CT^k (e)**
1	0.45	-32.66	-0.272	0.42	-24.49	0.299
2	0.09	-2.03	-0.015	0.09	-2.32	-0.015
3	0.07	-1.59	-0.009	0.07	-1.67	-0.009

Table S8. Eigenvalues ($|\nu_k|$), orbital interaction energies (ΔE_{oi}^k) and charge transfer (CT^k) associated to the first three NOCV deformation densities (k=1-3) and to the corresponding α and β components.

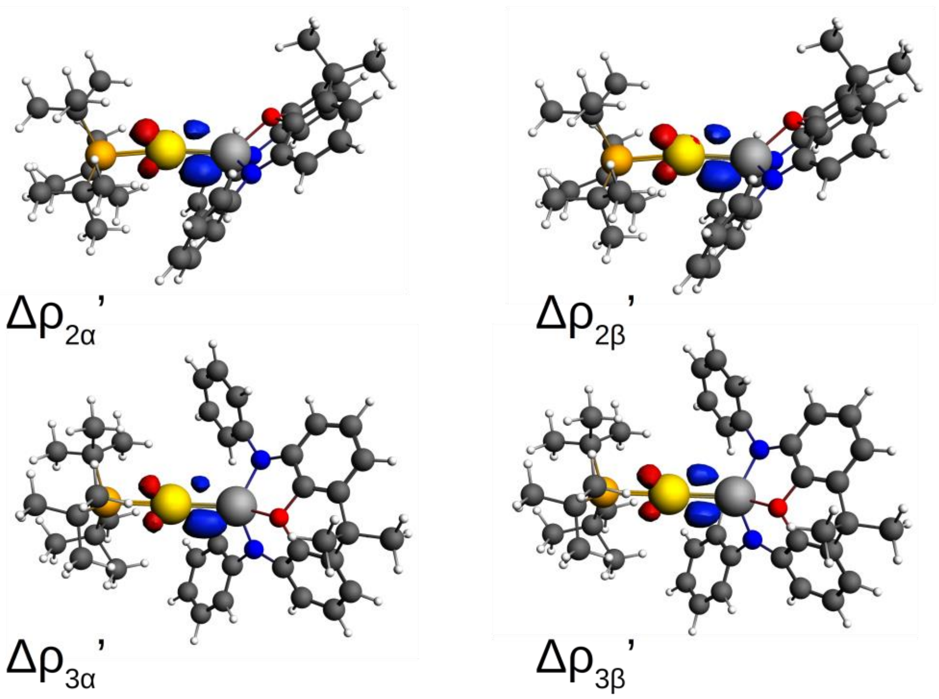

Figure S7. Isodensity surfaces of the $\Delta\rho_{2\alpha}$’ and $\Delta\rho_{2\beta}$ ’ (top) and $\Delta\rho_{3\alpha}$’ and $\Delta\rho_{3\beta}$ ’ (bottom) NOCV deformation densities. The isovalue for all the surfaces is 0.5 me/a_0^3. Blue regions indicate electron density accumulation areas, whereas red regions indicate depletion areas.

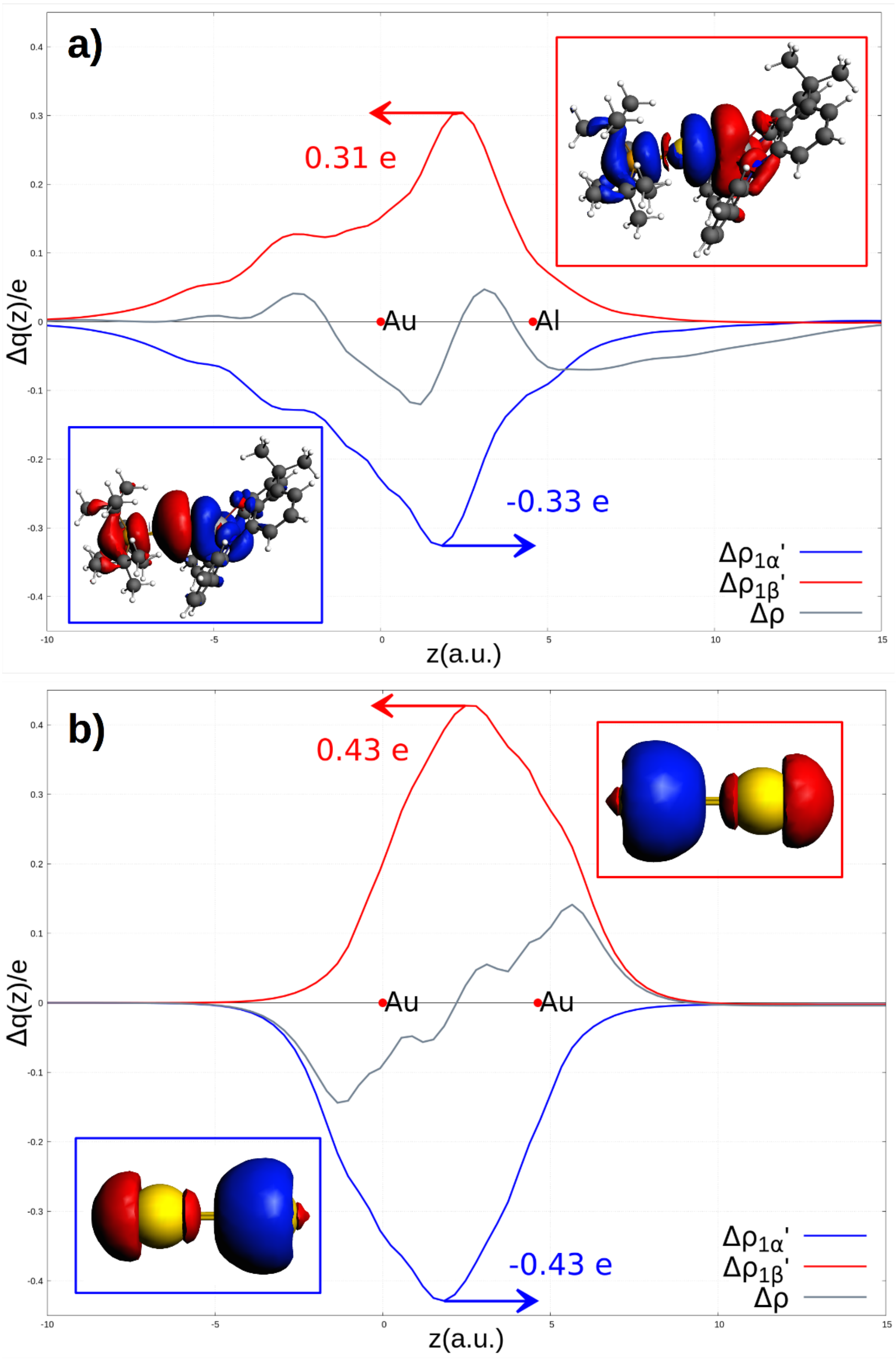

a)
0.31 e
-0.33 e
Au
Al
Δq(z)/e
z(a.u.)
Δρ1α'
Δρ1β'
Δρ
b)
0.43 e
-0.43 e
Au
Au
Δq(z)/e
z(a.u.)
Δρ1α'
Δρ1β'
Δρ

Figure S8. Charge Displacement (CD-NOCV) curves representing the electron-sharing interaction between neutral doublet open shell [^{t}Bu$_3$Au]• and [Al(NON')]• fragments in the [^{t}Bu$_3$AuAl(NON')] complex **(a)** and between Au atoms in the Au_2 dimer **(b)**. Positive values of the curve indicate right-to-left charge flux and viceversa (see Methodology section for details). *Insets:* isodensity surfaces of the corresponding NOCV deformation densities $\Delta\rho_1$'. The isovalue for all the surfaces is 0.3 me/a_0^3.

Blue regions indicate electron density accumulation areas, whereas red regions indicate depletion areas.

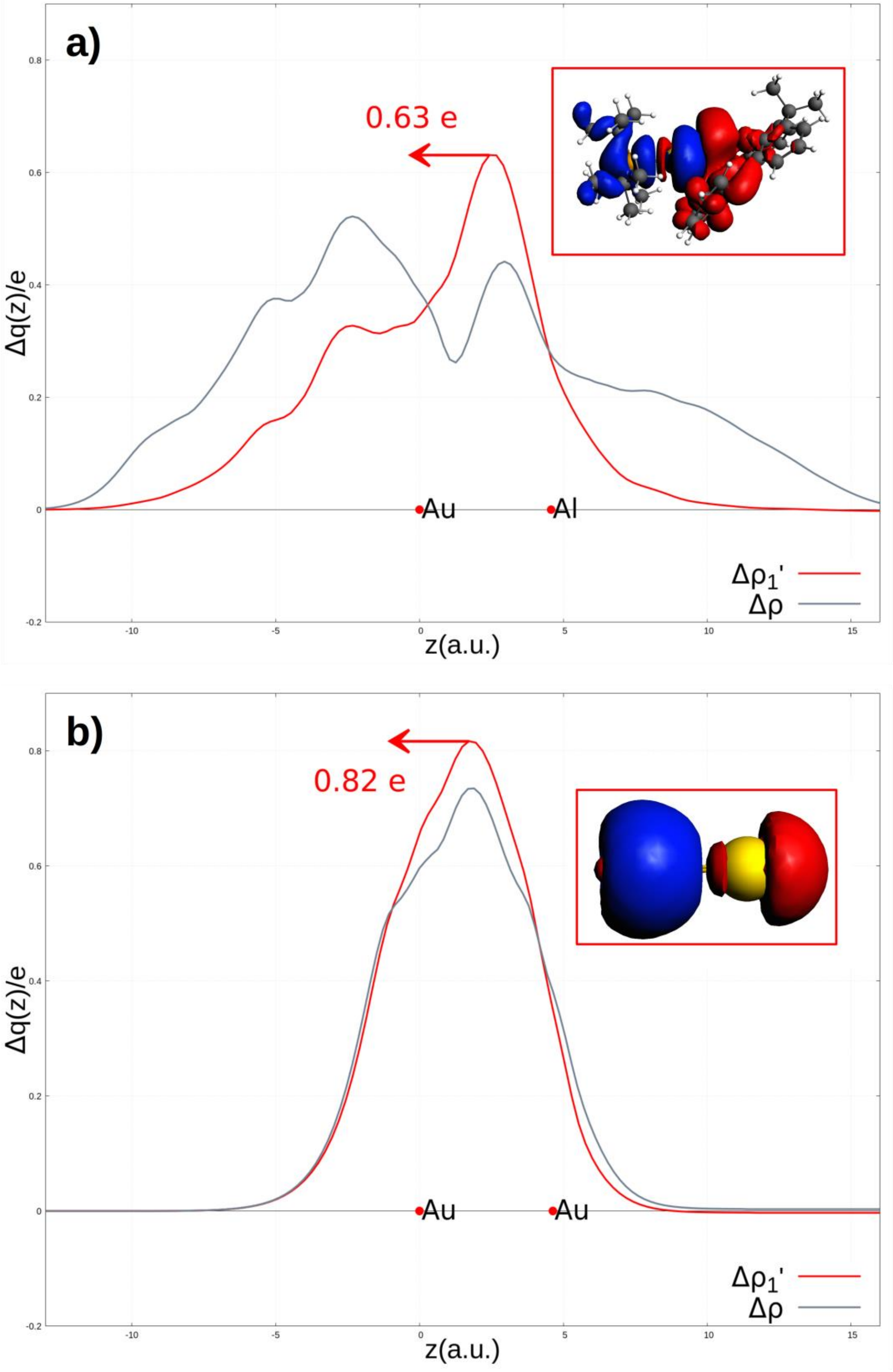

a)
0.63 e
Δq(z)/e
Au
Al
Δρ1'
Δρ
z(a.u.)
b)
0.82 e
Δq(z)/e
Au
Au
Δρ1'
Δρ
z(a.u.)

Figure S9. Charge Displacement (CD-NOCV) curves representing the dative interaction between charged closed shell [^{t}Bu$_3$Au]$^+$ and [AlNON']$^-$ fragments in the [^{t}Bu$_3$AuAl(NON')] complex **(a)** and between Au$^+$ and Au$^-$ in the Au$_2$ dimer **(b)**. Positive values of the curve indicate right-to-left charge flux and viceversa (see Methodology section for details). *Insets:* isodensity surfaces of the corresponding NOCV deformation densities $\Delta\rho_1$'. The isovalue for all the surfaces is 0.3 me/a$_0^3$. Blue regions indicate electron density accumulation areas, whereas red regions indicate depletion areas.

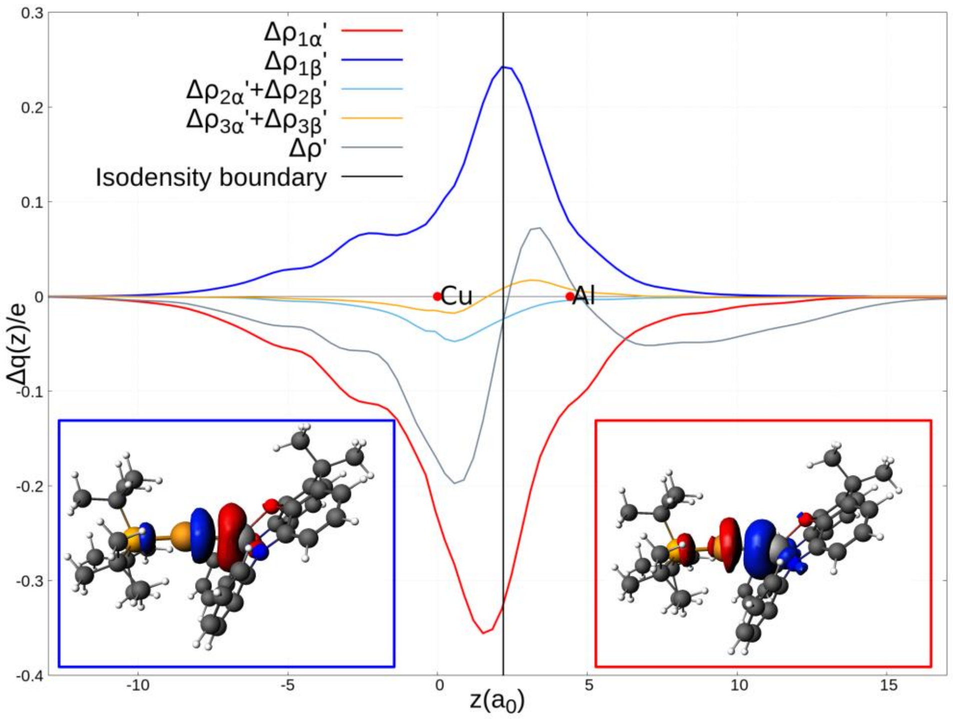

Figure S10. Charge Displacement (CD-NOCV) curves for the interaction between open shell doublet [^{t}Bu$_3$PCu]• and [Al(NON')]• fragments in the model [^{t}Bu$_3$PCuAl(NON')] complex. Red dots indicate the position of the main nuclei along the z axis. The vertical solid line marks the isodensity boundary between the fragments. Positive values of the curve indicate right-to-left charge flux and viceversa (see Methodology section for details). *Insets:* isodensity surfaces of the two NOCV deformation densities $\Delta\rho_{1\alpha}$' (red) and $\Delta\rho_{1\beta}$ ' (blue). The isovalue for all the surfaces is 1.5 me/a$_0^3$. Blue regions indicate electron density accumulation areas, whereas red regions indicate depletion areas.

	A			B		
**K**	**$\|\nu_k\|$**	**ΔE_{oi}^k (kcal/mol)**	**CT^k (e)**	**$\|\nu_k\|$**	**ΔE_{oi}^k (kcal/mol)**	**CT^k (e)**
1	0.50	-36.91	-0.324	0.32	-14.80	0.242
2	0.08	-1.22	-0.012	0.08	-1.35	-0.017
3	0.06	-1.18	0.015	0.06	-0.92	-0.006

Table S9. Eigenvalues ($|\nu_k|$), orbital interaction energies (ΔE_{oi}^k) and charge transfer (CT^k) associated to the first three NOCV deformation densities (k=1-3) and to the corresponding α and β components for the model [^{t}Bu$_3$PCuAl(NON')] complex.

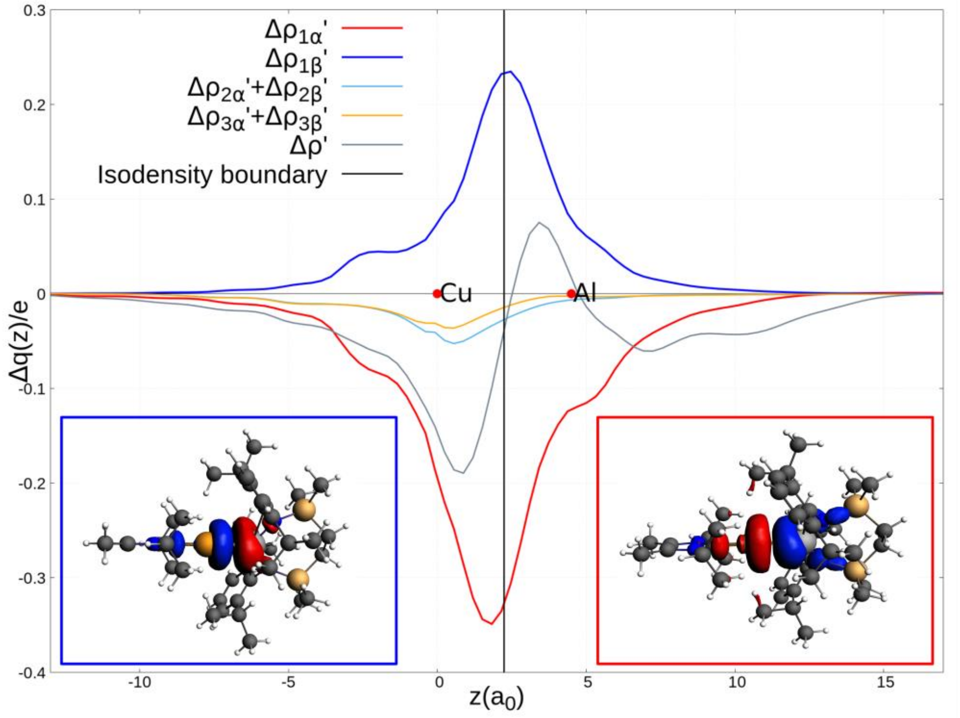

Figure S11. Charge Displacement (CD-NOCV) curves for the interaction between open shell doublet [NHC^{iPr}Cu]• and [AlSiN^{Dipp}]• fragments in the [NHC^{iPr}CuAlSiN^{Dipp}] complex. The structure of the complex has been optimized in Ref. [42] at the BP86/6-31G** level. Red dots indicate the position of the main nuclei along the z axis. The vertical solid line marks the isodensity boundary between the fragments. Positive values of the curve indicate right-to-left charge flux and viceversa (see Methodology section for details). *Insets:* isodensity surfaces of the two NOCV deformation densities $\Delta\rho_{1\alpha}$' (red) and $\Delta\rho_{1\beta}$' (blue). The isovalue for all the surfaces is 1 me/a_0^3. Blue regions indicate electron density accumulation areas, whereas red regions indicate depletion areas.

	**A**			**B**		
**K**	**$\|\nu_k\|$**	**ΔE_{oi}^k (kcal/mol)**	**CT^k (e)**	**$\|\nu_k\|$**	**ΔE_{oi}^k (kcal/mol)**	**CT^k (e)**
1	0.47	-37.67	-0.326	0.30	-13.52	-0.233
2	0.08	-1.32	-0.014	0.08	-1.36	-0.014
3	0.06	-0.96	-0.007	0.06	-0.94	-0.007

Table S10. Eigenvalues ($|\nu_k|$), orbital interaction energies (ΔE_{oi}^k) and charge transfer (CT^k) associated to the first three NOCV deformation densities (k=1-3) and to the corresponding α and β components for the [NHC^{iPr}CuAlSiN^{Dipp}] complex. The structure of the complex has been optimized in Ref. [42] at the BP86/6-31G** level.

	**[^{t}Bu$_3$PAu]· - [Al(NON')]·**	**[^{t}Bu$_3$PCu]· - [Al(NON')]·**	**[NHC^{iPr}Cu]· - [AlSiN^{Dipp}]·**
**ΔE_{Pauli}**	167.80	115.21	111.06
**ΔE_{Elst}**	-173.43	-126.30	-126.18
**ΔE_{Steric}**	-5.63	-11.28	-15.13
**ΔE_{oi}**	-71.51	-61.54	-63.57
**ΔE_{disp}**	-10.42	-9.97	-13.58
**ΔE**	-87.56	-82.80	-92.28

Table S11. Comparative Energy Decomposition Analysis (EDA) of the interaction energy between [$^{t}Bu_3PAu$]• and [Al(NON')]• open shell doublet fragments in the [$^{t}Bu_3PAuAl$(NON')] complex (first column, see also first column of **Table S7**), [$^{t}Bu_3PCu$]• and [Al(NON')]• open shell doublet fragments in the [$^{t}Bu_3PCuAl$(NON')] complex (second column) and [$NHC^{iPr}Cu$]• and [$AlSiN^{Dipp}$]• open shell doublet fragments in the [$NHC^{iPr}CuAlSiN^{Dipp}$] complex **III** (third column). Energies are given in kcal/mol.

	**Au (eV)**	**Al (eV)**
**IE_M**	9.51 (9.22[44])	5.90 (5.98[43])
**EA_M**	-2.19 (-2.31[45])	-0.07 (-0.43[46])
**χ_M**	3.66 (3.45)	1.54 (1.26)

Table S12. Atomic ionization energy (IE_M), atomic electron affinity (EA_M) and electronegativity (χ_M) calculated by applying Mulliken's definition (i.e. $\chi_M = (EI_M + EA_M)/2$)[47]. The experimental values (Refs. 43-46) are reported in parenthesis.

	**$AuP^{t}Bu_3$ (eV)**	**AlNON' (eV)**
**IE_{MX}**	5.39	6.33
**EA_{MX}**	-0.26	-1.26
**χ_{MX}**	2.56	2.53

Table S13. Molecular ionization energy (IE_{MX} associated to the process $MX\cdot \rightarrow MX^{+} + e^{-}$), molecular electron affinity (EA_{MX} associated to the process $MX\cdot + e^{-} \rightarrow MX^{-}$) and "molecular electronegativity" (χ_{MX}, calculated by extending the Mulliken's atomic definition, i.e. $\chi_{MX} = (EI_{MX} + AE_{MX})/2$)[47] evaluated for the [$^{t}Bu_3PAu$] and [Al(NON')] fragments.

Method	q_{Au}	q_{Al}
**AIM**	-0.83	+2.18
**Mulliken**	+0.22	+0.26
**Hirshfeld**	-0.01	+0.28
**VDD**	-0.08	+0.18
**Multipole Derived**	+0.16	+0.24
**CM5**	-0.18	+1.07

Table S14. Atomic charges on Au (q_{Au}) and Al (q_{Al}) in the [^{t}Bu$_3$PAuAl(NON')] complex calculated with different approaches (see Computational Details).

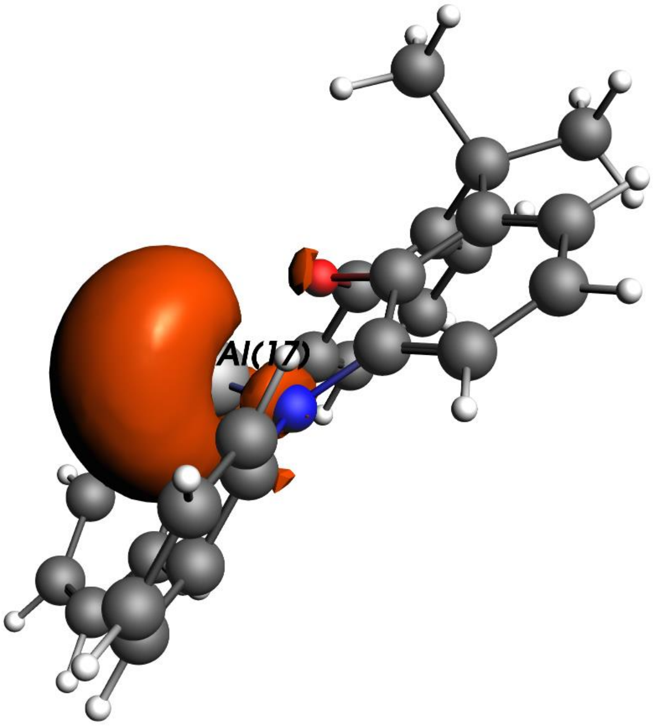

Figure S12. Electron density associated with the HOMO of the aluminyl anion [Al(NON')]$^{-}$. The isodensity value is of 0.003 e/au^3.

References

(1) Mitoraj, M.; Michalak, A. Natural Orbitals for Chemical Valence as Descriptors of Chemical Bonding in Transition Metal Complexes. *J. Mol. Model.* **2007**, *13* (2), 347–355.

(2) Michalak, A.; Mitoraj, M.; Ziegler, T. Bond Orbitals from Chemical Valence Theory. *J. Phys. Chem. A* **2008**, *112* (9), 1933–1939.

(3) Nalewajski, R. F.; Mrozek, J. Modified Valence Indices from the Two□particle Density Matrix. *Int. J. Quantum Chem.* **1994**, *51* (4), 187–200.

(4) Nalewajski, R. F.; Mrozek, J.; Michalak, A. Two-Electron Valence Indices from the Kohn-Sham Orbitals. *Int. J. Quantum Chem.* **1997**, *61* (3), 589–601.

(5) Lu, T.; Chen, F. Bond Order Analysis Based on the Laplacian of Electron Density in Fuzzy Overlap Space. *J. Phys. Chem. A* **2013**, *117* (14), 3100–3108.

(6) Belpassi, L.; Infante, I.; Tarantelli, F.; Visscher, L. The Chemical Bond between Au(I) and the Noble Gases. Comparative Study of NgAuF and NgAu+ (Ng = Ar, Kr, Xe) by Density Functional and Coupled Cluster Methods. *J. Am. Chem. Soc.* **2008**, *130* (3), 1048–1060.

(7) Bistoni, G.; Rampino, S.; Tarantelli, F.; Belpassi, L. Charge-Displacement Analysis via Natural Orbitals for Chemical Valence: Charge Transfer Effects in Coordination Chemistry. *J. Chem. Phys.* **2015**, *142* (8), 084112.

(8) Salvi, N.; Belpassi, L.; Tarantelli, F. On the Dewar-Chatt-Duncanson Model for Catalytic Gold(I) Complexes. *Chem. - A Eur. J.* **2010**, *16* (24), 7231–7240.

(9) Bistoni, G.; Belpassi, L.; Tarantelli, F. Advances in Charge Displacement Analysis. *J. Chem. Theory Comput.* **2016**, *12* (3), 1236–1244.

(10) Bickelhaupt, F. M.; Baerends, E. J. Kohn-Sham Density Functional Theory: Predicting and Understanding Chemistry. In *Reviews in computational chemistry*; Wiley-VCH Verlag, 2007; Vol. 15, pp 1–86.

(11) Mitoraj, M. P.; Michalak, A.; Ziegler, T. A Combined Charge and Energy Decomposition Scheme for Bond Analysis. *J. Chem. Theory Comput.* **2009**, *5* (4), 962–975.

(12) Ferná, I.; Bickelhaupt, F. M. The Activation Strain Model and Molecular Orbital Theory: Understanding and Designing Chemical Reactions. *Chem. Soc. Rev* **2014**, *43*, 4953.

(13) Bickelhaupt, F. M.; Houk, K. N. Analyzing Reaction Rates with the Distortion/Interaction-Activation Strain Model. *Angewandte Chemie - International Edition*. **2017**, *56*, 10070–10086.

(14) Vermeeren, P.; van der Lubbe, S. C. C.; Fonseca Guerra, C.; Bickelhaupt, F. M.; Hamlin, T. A. Understanding Chemical Reactivity Using the Activation Strain Model. *Nat. Protoc.* **2020**, *15* (2), 649–667.

(15) te Velde, G.; Bickelhaupt, F. M.; Baerends, E. J.; Fonseca Guerra, C.; van Gisbergen, S. J. A.; Snijders, J. G.; Ziegler, T. Chemistry with ADF. *J. Comput. Chem.* **2001**, *22* (9), 931–967.

(16) *ADF Manual ADF Program System Release 2014*.

(17) Swart, M.; Bickelhaupt, F. M. QUILD: QUantum-Regions Interconnected by Local Descriptions. *J. Comput. Chem.* **2008**, *29* (5), 724–734.

(18) Perdew, J. P.; Burke, K.; Ernzerhof, M. Generalized Gradient Approximation Made Simple. *Phys. Rev. Lett.* **1996**, *77* (18), 3865–3868.

(19) Van Lenthe, E.; Baerends, E. J.; Snijders, J. G. Relativistic Regular Two-Component Hamiltonians. *J. Chem. Phys.* **1993**, *99* (6), 4597–4610.

(20) Van Lenthe, E.; Baerends, E. J.; Snijders, J. G. Relativistic Total Energy Using Regular Approximations. *J. Chem. Phys.* **1994**, *101* (11), 9783–9792.

(21) Van Lenthe, E. Geometry Optimizations in the Zero Order Regular Approximation for Relativistic Effects. *J. Chem. Phys.* **1999**, *110* (18), 8943–8953.

(22) Grimme, S.; Antony, J.; Ehrlich, S.; Krieg, H. A Consistent and Accurate Ab Initio Parametrization of Density Functional Dispersion Correction (DFT-D) for the 94 Elements H-Pu. *J. Chem. Phys.* **2010**, *132* (15), 154104.

(23) Grimme, S.; Ehrlich, S.; Goerigk, L. Effect of the Damping Function in Dispersion Corrected Density Functional Theory. *J. Comput. Chem.* **2011**, *32* (7), 1456–1465.

(24) Pye, C. C.; Ziegler, T. An Implementation of the Conductor-like Screening Model of Solvation within the Amsterdam Density Functional Package. *Theor. Chem. Acc.* **1999**, *101* (6), 396–408.

(25) Hicks, J.; Mansikkamäki, A.; Vasko, P.; Goicoechea, J. M.; Aldridge, S. A Nucleophilic Gold Complex. *Nat. Chem.* **2019**, *11* (3), 237–241.

(26) Fonseca Guerra, C.; Handgraaf, J.-W.; Baerends, E. J.; Bickelhaupt, F. M. Voronoi Deformation Density (VDD) Charges: Assessment of the Mulliken, Bader, Hirshfeld, Weinhold, and VDD Methods for Charge Analysis. *J. Comput. Chem.* **2004**, *25* (2), 189–210.

(27) Mulliken, R. S. Electronic Population Analysis on LCAO-MO Molecular Wave Functions. I. *J. Chem. Phys.* **1955**, *23* (10), 1833–1840.

(28) Hirshfeld, F. L. Bonded-Atom Fragments for Describing Molecular Charge Densities. *Theor. Chim. Acta* **1977**, *44* (2), 129–138.

(29) Swart, M.; Van Duijnen, P. T.; Snijders, J. G. A Charge Analysis Derived from an Atomic Multipole Expansion *J. Comput. Chem.* **2001**, *22*, 79-88. https://doi.org/10.1002/1096-987X.

(30) Marenich, A. V.; Jerome, S. V.; Cramer, C. J.; Truhlar, D. G. Charge Model 5: An Extension of Hirshfeld Population Analysis for the Accurate Description of Molecular Interactions in Gaseous and Condensed Phases. *J. Chem. Theory Comput.* **2012**, *8* (2), 527–541.

(31) Rodríguez, J. I.; Bader, R. F. W.; Ayers, P. W.; Michel, C.; Götz, A. W.; Bo, C. A High Performance Grid-Based Algorithm for Computing QTAIM Properties. *Chem. Phys. Lett.* **2009**, *472* (1–3), 149–152.

(32) Rodríguez, J. I.; Köster, A. M.; Ayers, P. W.; Santos-Valle, A.; Vela, A.; Merino, G. An Efficient Grid-Based Scheme to Compute QTAIM Atomic Properties without Explicit Calculation of Zero-Flux Surfaces. *J. Comput. Chem.* **2009**, *30* (7), 1082–1092.

(33) Mayer, I. Charge, Bond Order and Valence in the AB Initio SCF Theory. *Chem. Phys. Lett.* **1983**, *97* (3), 270–274.

(34) Neese, F. The ORCA Program System. *Wiley Interdiscip. Rev. Comput. Mol. Sci.* **2012**, *2* (1), 73–78.

(35) Weigend, F.; Ahlrichs, R. Balanced Basis Sets of Split Valence, Triple Zeta Valence and Quadruple Zeta Valence Quality for H to Rn: Design and Assessment of Accuracy. *Phys. Chem. Chem. Phys.* **2005**, *7* (18), 3297–3305.

(36) Compton, R.N.; Reinhardt, P.W.; Cooper, C.D. Collisional ionization of Na, K, and Cs by CO_2, COS, and CS_2: molecular electron affinities. *J. Chem. Phys.* **1975**, *63*, 3821-3827.

(37) (a) Jacox, M.E.; Thompson, W.E. The vibrational spectra of molecular ions isolated in solid neon. I. CO_2^+ and CO_2^-. *J. Chem. Phys.* **1989**, *91*, 1410-1416. (b) Ovenall, D.W.; Whiffen, D.H. Electron spin resonance and structure of the CO_2^- radical ion. *Mol. Phys.* **1961**, *4*, 135-144. (c) Schröder, D.; Schalley, C.A.; Harvey, J.N.; Schwarz, H. On the formation of the carbon dioxide anion radical $CO_2^{-\cdot}$ in the gas phase. *Int. J. Mass Spec.* **1999**, *185/186/187*, 25-35.

(38) Hendy, C.M.; Smith, G.C.; Xu, Z.; Lian, T.; Jui, N.T. Radical chain reduction via carbon dioxide radical anion ($CO_2^{\cdot-}$). *J. Am. Chem. Soc.* **2021**, *143*, 8987-8992.

(39) Hicks, J.; Vasko, P.; Goicoechea, J. M.; Aldridge, S. The Aluminyl Anion: A New Generation of Aluminium Nucleophile. *Angewandte Chemie - International Edition*. **2021**, *60*, 1702–1713.

(40) Zhao, L.; von Hopffgarten, M.; Andrada, D. M.; Frenking, G. Energy Decomposition Analysis. *WIREs Comput. Mol. Sci.* **2018**, *8*, 1345.

(41) Jerabek, P.; Schwerdtfeger, P.; Frenking, G. Dative and Electron-Sharing Bonding in Transition Metal Compounds. *J. Comput. Chem.* **2019**, *40* (1), 247–264.

(42) Liu, H.; Schwamm, R. J.; Hill, M. S.; Mahon, M. F.; McMullin, C. L.; Rajabi, N. A. Ambiphilic Al−Cu Bonding. *Angew. Chemie Int. Ed.* **2021**.

(43) Kaufman, V.; Martin, W. C. Wavelengths and Energy Level Classifications for the Spectra of Aluminum (AlI through AlXIII). *J. Phys. Chem. Ref. Data* **1991**, *20* (5), 775–858.

(44) Andersen, T.; Haugen, H. K.; Hotop, H. Binding Energies in Atomic Negative Ions: III. *J. Phys. Chem. Ref. Data* **1999**, *28* (6), 1511–1533.

(45) Dyubko, S. F.; Efremov, V. A.; Gerasimov, V. G.; MacAdam, K. B. Millimetre-Wave Spectroscopy of Au I Rydberg States: S, P and D Terms. *J. Phys. B At. Mol. Opt. Phys.* **2005**, *38* (8), 1107–1118.

(46) Scheer, M.; Bilodeau, R. C.; Thøgersen, J.; Haugen, H. K. Threshold Photodetachment of Al^- Electron Affinity and Fine Structure. *Phys. Rev. A - At. Mol. Opt. Phys.* **1998**, *57* (3), R1493–R1496.

(47) Mulliken, R. S. A New Electroaffinity Scale; Together with Data on Valence States and on Valence Ionization Potentials and Electron Affinities. *J. Chem. Phys.* **1934**, *2* (11), 782–793. https://doi.org/10.1063/1.1749394.

Geometries xyz

[$^{t}Bu_3$PAuAl(NON')]

94

C	3.157353	-2.086529	0.366848
C	3.768196	-1.124431	-0.452422
C	5.064406	-1.136429	-0.931871
C	5.811438	-2.290822	-0.654068
C	5.231750	-3.320347	0.096267
C	3.938024	-3.234568	0.615521
C	5.554006	0.118227	-1.668056
C	4.997830	1.319391	-0.890032
C	3.703325	1.223330	-0.413971
O	2.977124	0.032476	-0.654717
C	3.039088	2.128007	0.429550
C	3.760079	3.304851	0.720397
C	5.049454	3.473992	0.210551
C	5.682951	2.501310	-0.571548
N	1.818823	1.693731	0.926118
C	0.897322	2.563362	1.526363
AL	1.209556	-0.032950	0.314141
AU	-1.105485	-0.044560	-0.375205
P	-3.494501	0.013091	-0.764470
C	-3.896483	0.155175	-2.634387
C	-2.920358	-0.767982	-3.391675
C	7.079479	0.161345	-1.759459
C	4.960134	0.125679	-3.099364
N	1.922336	-1.729543	0.885072
C	1.071217	-2.659541	1.501356
C	-4.267864	-1.596830	-0.062568
C	-3.556481	-1.925604	1.265361
C	-4.207440	1.532865	0.164102
C	-3.249780	2.719820	-0.065459
C	-5.785327	-1.555521	0.155743
C	-3.922116	-2.757229	-1.011147
C	-5.636666	1.929673	-0.227110
C	-4.160203	1.246521	1.674524
C	-5.341729	-0.179574	-3.023835
C	-3.569076	1.583299	-3.101468
H	3.548873	-4.031595	1.246139
H	6.838932	-2.382296	-1.000806
H	6.704818	2.657456	-0.911588
H	3.329664	4.059820	1.375479
H	7.403896	1.063452	-2.293259
H	7.451590	-0.703221	-2.323383
H	7.544533	0.156758	-0.765280
H	5.269516	1.037799	-3.627492
H	3.864133	0.092209	-3.078580
H	5.322926	-0.747484	-3.658417

H	5.820580	-4.213289	0.310911
H	5.591198	4.387962	0.458024
H	-4.205246	-3.696780	-0.513132
H	-4.464859	-2.711874	-1.960968
H	-2.843686	-2.800524	-1.214268
H	-3.885251	-2.923811	1.592995
H	-2.466001	-1.956586	1.136270
H	-3.783885	-1.219688	2.067681
H	-6.116659	-2.545007	0.508503
H	-6.074310	-0.825002	0.919984
H	-6.336951	-1.328667	-0.763482
H	-3.033271	-0.579701	-4.470523
H	-1.880040	-0.550681	-3.110653
H	-3.104545	-1.830851	-3.217956
H	-3.633246	1.603710	-4.199942
H	-4.271568	2.329794	-2.717624
H	-2.548651	1.875404	-2.819880
H	-5.461554	-0.017524	-4.106789
H	-5.596281	-1.226005	-2.822077
H	-6.070565	0.457845	-2.510409
H	-3.567307	3.548135	0.585800
H	-2.218846	2.456939	0.209332
H	-3.250419	3.085277	-1.095137
H	-5.951261	2.774840	0.405345
H	-5.708353	2.260659	-1.269420
H	-6.354566	1.115730	-0.074616
H	-4.389631	2.183998	2.203114
H	-4.895830	0.499522	1.990463
H	-3.158422	0.926310	1.989806
C	0.689168	3.890363	1.101250
C	-0.303886	4.674813	1.682789
C	-1.124362	4.162193	2.692111
C	-0.938149	2.844028	3.110623
C	0.061307	2.057676	2.541216
H	1.293159	4.294568	0.289882
H	-0.447857	5.697720	1.331154
H	-1.900777	4.781643	3.141105
H	-1.569660	2.423468	3.894301
H	0.218577	1.036632	2.892867
C	0.277517	-2.230569	2.581714
C	-0.654538	-3.081507	3.172642
C	-0.811605	-4.387871	2.707600
C	-0.029275	-4.825161	1.634194
C	0.896050	-3.975781	1.032369
H	0.416029	-1.218841	2.966809
H	-1.257079	-2.720570	4.007189
H	-1.535362	-5.057390	3.172525
H	-0.150510	-5.839107	1.249471
H	1.471726	-4.319712	0.173640

RC

97

C	2.981248	-1.914968	0.860024
C	3.653494	-1.137979	-0.097869
C	4.945351	-1.309848	-0.557856
C	5.618163	-2.447633	-0.087291
C	4.976283	-3.300328	0.817836
C	3.689891	-3.051426	1.303140
C	5.513249	-0.227745	-1.487046
C	5.033579	1.118999	-0.927492
C	3.737242	1.184128	-0.453066
O	2.939043	0.017191	-0.492343
C	3.126827	2.259152	0.211594
C	3.915557	3.424629	0.300234
C	5.212806	3.425737	-0.217857
C	5.787258	2.296533	-0.813017
N	1.880787	1.989548	0.759592
C	1.013200	3.003373	1.192816
AL	1.174500	0.226407	0.439428
AU	-1.124192	0.107501	-0.293325
P	-3.505173	-0.033135	-0.712915
C	-4.254076	-1.189980	0.620606
C	-4.195652	-0.466834	1.976977
C	7.038628	-0.295146	-1.567755
C	4.920999	-0.421813	-2.905982
N	1.767692	-1.400209	1.292450
C	0.858182	-2.138588	2.062811
C	-4.251859	1.726125	-0.540222
C	-3.882949	2.538147	-1.793010
C	-3.893929	-0.727773	-2.458485
C	-3.607199	-2.238386	-2.469006
C	-5.770370	1.775841	-0.330707
C	-3.539922	2.431243	0.632002
C	-5.325920	-0.490905	-2.955224
C	-2.884956	-0.100488	-3.441535
C	-5.692988	-1.650851	0.357058
C	-3.327456	-2.414675	0.759735
C	0.560904	-2.617790	-2.009554
O	-0.185810	-3.346214	-1.479403
O	1.313934	-1.906421	-2.554487
H	3.255866	-3.707108	2.055155
H	6.637160	-2.659078	-0.405413
H	6.815899	2.331749	-1.166595
H	3.530203	4.306908	0.807778
H	7.419069	0.483794	-2.240435
H	7.356037	-1.262833	-1.976196
H	7.502495	-0.162452	-0.581839
H	5.285661	0.370685	-3.573358
H	3.825235	-0.388664	-2.891225
H	5.231731	-1.395128	-3.309563
H	5.508983	-4.179527	1.182924
H	5.808049	4.335620	-0.128775
H	-2.998348	-0.597251	-4.417227
H	-3.034911	0.971341	-3.593024
H	-1.853200	-0.253667	-3.094087
H	-3.649276	-2.582799	-3.513581

H	-2.606534	-2.465831	-2.080840
H	-4.345350	-2.816158	-1.903510
H	-5.442592	-0.976123	-3.937101
H	-6.076382	-0.920702	-2.281744
H	-5.552672	0.572525	-3.089405
H	-3.655956	-2.995702	1.634605
H	-3.349319	-3.077445	-0.108041
H	-2.286423	-2.110225	0.935121
H	-4.447492	-1.197349	2.760480
H	-3.185233	-0.090495	2.186238
H	-4.911240	0.358587	2.051757
H	-6.025689	-2.264523	1.209073
H	-6.392153	-0.812795	0.258720
H	-5.772868	-2.273337	-0.541244
H	-4.154094	3.588295	-1.607247
H	-2.802846	2.503103	-1.988642
H	-4.420096	2.214091	-2.690216
H	-6.086667	2.830520	-0.299020
H	-6.323994	1.286979	-1.140235
H	-6.071672	1.318462	0.618626
H	-3.860835	3.484170	0.646204
H	-3.773793	1.999237	1.607775
H	-2.448656	2.413678	0.506662
C	0.058566	-1.446911	2.993496
C	-0.925578	-2.102768	3.729463
C	-1.131495	-3.473169	3.566554
C	-0.349377	-4.170078	2.641166
C	0.626840	-3.517150	1.892817
H	0.236442	-0.381749	3.150017
H	-1.528652	-1.538006	4.441684
H	-1.894675	-3.991350	4.147254
H	-0.511842	-5.237936	2.486592
H	1.194530	-4.070026	1.146090
C	0.873356	4.239718	0.533269
C	-0.075362	5.166319	0.958529
C	-0.915816	4.888341	2.040744
C	-0.792984	3.662141	2.694938
C	0.162333	2.736123	2.281872
H	1.493277	4.457997	-0.335524
H	-0.168609	6.114535	0.426656
H	-1.657484	5.617731	2.366619
H	-1.439886	3.424262	3.540435
H	0.271831	1.790681	2.815222

TSI

97

C	-3.116890	2.130797	0.493993
C	-3.895336	1.162754	-0.155448
C	-5.251527	1.204967	-0.411651
C	-5.900679	2.394251	-0.043568
C	-5.166125	3.424034	0.555363
C	-3.800576	3.313235	0.836104

C	-5.889280	-0.036324	-1.052622
C	-5.225574	-1.263219	-0.410579
C	-3.870434	-1.193254	-0.151957
O	-3.174004	-0.006347	-0.455077
C	-3.084557	-2.131951	0.532906
C	-3.749224	-3.324624	0.879506
C	-5.107498	-3.468548	0.582080
C	-5.856091	-2.459552	-0.034381
N	-1.800034	-1.712114	0.853427
C	-0.852603	-2.603940	1.393264
AL	-1.280906	-0.007198	0.192208
O	-1.200938	-0.920755	-2.207990
C	-0.261170	-0.158171	-2.326172
O	0.389368	0.520229	-3.076803
C	-7.407678	-0.051338	-0.868615
C	-5.560391	-0.034062	-2.567438
N	-1.812337	1.739487	0.772335
C	-0.859120	2.653137	1.257361
AU	1.113205	0.045722	-0.365882
P	3.568714	0.005978	-0.396348
C	4.210834	1.768159	-0.791349
C	4.020180	2.655691	0.449071
C	4.234257	-0.560954	1.307497
C	4.010444	-2.074362	1.452336
C	4.145396	-1.217433	-1.756446
C	3.882395	-0.586545	-3.134322
C	5.714324	-0.246890	1.561690
C	3.361111	0.102324	2.390859
C	5.620372	-1.631618	-1.667670
C	3.244913	-2.467532	-1.684082
C	5.676082	1.836463	-1.242211
C	3.298398	2.371547	-1.877489
H	-3.278869	4.120653	1.346665
H	-6.968971	2.516576	-0.211778
H	-6.920872	-2.602290	-0.208057
H	-3.217861	-4.112410	1.410480
H	-7.840440	-0.939276	-1.346117
H	-7.857807	0.827185	-1.347579
H	-7.686415	-0.053304	0.193078
H	-5.983260	-0.929743	-3.042297
H	-4.477179	-0.030545	-2.740778
H	-5.992252	0.856890	-3.043168
H	-5.681001	4.343840	0.835922
H	-5.606662	-4.395650	0.867074
H	3.485670	-3.111092	-2.543818
H	3.386416	-3.056481	-0.775065
H	2.182521	-2.195269	-1.749819
H	4.050305	-1.363064	-3.895863
H	2.845990	-0.240043	-3.234605
H	4.560329	0.243172	-3.358936
H	5.848489	-2.291220	-2.519373
H	6.300015	-0.773950	-1.722022
H	5.845232	-2.192042	-0.753650
H	4.227035	-2.347918	2.495990

H	2.969530	-2.354702	1.251792
H	4.674023	-2.667008	0.814731
H	3.660495	-0.301913	3.370134
H	3.465321	1.188987	2.428314
H	2.299613	-0.135012	2.234461
H	5.994344	-0.661667	2.542619
H	6.372030	-0.699533	0.810943
H	5.915856	0.829235	1.593640
H	5.947079	2.894104	-1.385182
H	6.363709	1.415240	-0.500308
H	5.844033	1.326827	-2.197401
H	3.599023	3.419378	-2.029797
H	3.364523	1.860427	-2.840231
H	2.246862	2.361426	-1.559270
H	4.228076	3.695000	0.153103
H	2.987818	2.622471	0.819486
H	4.703434	2.405467	1.267152
C	0.011544	2.245417	2.283566
C	1.014699	3.093568	2.750994
C	1.168557	4.369352	2.207684
C	0.312839	4.780986	1.181278
C	-0.687117	3.935968	0.706593
H	-0.128227	1.260054	2.731215
H	1.672745	2.755854	3.552517
H	1.949354	5.035145	2.575445
H	0.433157	5.769309	0.735233
H	-1.329963	4.255791	-0.113176
C	-0.605740	-3.870322	0.835535
C	0.382177	-4.695763	1.368325
C	1.146971	-4.278441	2.461688
C	0.915848	-3.017603	3.014026
C	-0.074211	-2.189878	2.487657
H	-1.180699	-4.190989	-0.033032
H	0.564996	-5.671659	0.916164
H	1.919034	-4.928094	2.874287
H	1.507346	-2.674798	3.863890
H	-0.273995	-1.214474	2.934755

INT

97

C	1.736682	-2.187848	1.587433
C	2.468823	-1.919311	0.419431
C	3.348922	-2.754901	-0.236098
C	3.462815	-4.043656	0.318654
C	2.725686	-4.389723	1.454818
C	1.873885	-3.487737	2.102301
C	4.118717	-2.285612	-1.479162
C	3.990060	-0.765408	-1.655894
C	3.085096	-0.035013	-0.915538
O	2.211204	-0.627472	-0.014987
C	2.920044	1.360094	-0.893440
C	3.702370	2.075910	-1.814700

C	4.603050	1.378414	-2.628305
C	4.768379	-0.007279	-2.551790
N	2.003484	1.819792	0.040840
C	1.867615	3.230327	0.191842
AL	1.072044	0.565185	1.137291
C	-0.882674	1.693343	2.019953
O	-1.777009	2.307842	2.583473
C	5.608685	-2.652445	-1.324061
C	3.549983	-2.998486	-2.726249
N	1.019022	-1.107941	2.085126
C	0.045561	-1.362490	3.090308
AU	-1.421547	0.577266	0.324902
P	-2.432442	-0.501034	-1.557911
C	-1.260322	-1.818462	-2.294708
C	-0.628466	-2.612517	-1.135637
O	0.387566	1.700999	2.394476
C	-4.058612	-1.315153	-0.957392
C	-4.722881	-0.364883	0.059505
C	-2.813303	0.849771	-2.862519
C	-1.603734	1.802029	-2.946701
C	-5.048521	-1.662860	-2.076742
C	-3.715271	-2.593197	-0.174594
C	-3.146299	0.311957	-4.260232
C	-3.984505	1.709196	-2.356653
C	-1.927281	-2.788532	-3.278794
C	-0.105423	-1.089218	-3.000508
H	1.334170	-3.781849	3.000777
H	4.127527	-4.777608	-0.134818
H	5.504426	-0.496895	-3.187871
H	3.622437	3.159825	-1.877099
H	6.176206	-2.348611	-2.212219
H	5.730009	-3.736316	-1.207010
H	6.041739	-2.155953	-0.446549
H	4.090796	-2.681191	-3.627891
H	2.487893	-2.760059	-2.859853
H	3.649275	-4.087227	-2.621525
H	2.828749	-5.395611	1.863853
H	5.208900	1.943208	-3.338045
H	-5.609060	-0.871767	0.470258
H	-4.047095	-0.143922	0.898464
H	-5.052538	0.581961	-0.373463
H	-4.635816	-2.942929	0.315959
H	-3.357445	-3.404128	-0.816727
H	-2.974921	-2.399872	0.612430
H	-5.914174	-2.174442	-1.628715
H	-5.427842	-0.774347	-2.592926
H	-4.614801	-2.337707	-2.823318
H	0.641030	-1.842272	-3.289682
H	-0.418287	-0.577496	-3.915684
H	0.390477	-0.367497	-2.337387
H	0.207340	-3.205602	-1.534778
H	-0.227435	-1.943417	-0.364547
H	-1.325223	-3.305267	-0.658168
H	-1.154884	-3.465227	-3.675200

H	-2.687798	-3.411738	-2.795213
H	-2.388404	-2.276398	-4.130049
H	-3.414835	1.163452	-4.903986
H	-2.291746	-0.190476	-4.726772
H	-3.995363	-0.380304	-4.254056
H	-4.083455	2.571883	-3.031896
H	-4.941641	1.178731	-2.360281
H	-3.796080	2.100121	-1.347642
H	-1.872696	2.636632	-3.611141
H	-1.364599	2.219422	-1.959841
H	-0.699722	1.336541	-3.344551
C	0.065882	-0.656498	4.299125
C	-0.935185	-0.858307	5.250195
C	-1.961307	-1.774570	5.016299
C	-1.981837	-2.490223	3.815861
C	-0.989896	-2.283285	2.860365
H	0.860257	0.066776	4.476410
H	-0.911214	-0.290723	6.181080
H	-2.742632	-1.927524	5.761141
H	-2.782456	-3.204594	3.618001
H	-1.005441	-2.833284	1.919158
C	0.661222	3.863894	-0.124322
C	0.509236	5.237469	0.066377
C	1.569226	5.998757	0.559987
C	2.780569	5.373711	0.869084
C	2.928638	3.999214	0.690671
H	-0.162988	3.260941	-0.504310
H	-0.443094	5.713460	-0.170265
H	1.451087	7.072070	0.711049
H	3.611780	5.958535	1.265206
H	3.866001	3.504740	0.948173

TSII

97

C	3.308890	-0.825975	1.989856
C	2.928637	-1.853870	1.111070
C	3.249374	-3.194889	1.181185
C	3.952538	-3.588095	2.329589
C	4.305531	-2.623840	3.280831
C	4.012321	-1.265472	3.129659
C	2.851882	-4.080733	-0.012698
C	3.157457	-3.267047	-1.281169
C	2.879001	-1.915890	-1.243271
O	2.281436	-1.388576	-0.066622
C	3.256565	-0.934539	-2.172279
C	3.808229	-1.444023	-3.365024
C	4.030857	-2.817930	-3.491401
C	3.751904	-3.728843	-2.464604
N	3.032612	0.385149	-1.784852
C	3.648433	1.489294	-2.418324
AL	2.190543	0.537778	-0.122331
O	0.586311	1.135577	0.017758

C	-0.438028	1.734216	-0.651993
O	-0.230749	2.730737	-1.333199
C	3.610326	-5.408285	-0.004442
C	1.329444	-4.365350	0.055018
N	3.021187	0.465045	1.555454
C	3.148180	1.614651	2.351791
AU	-2.239220	0.789165	-0.322873
P	-4.241675	-0.356330	0.102743
C	-5.298939	0.648331	1.347137
C	-5.224294	2.136155	0.949288
C	-5.213013	-0.601125	-1.530236
C	-4.205590	-0.962325	-2.640283
C	-3.793537	-2.062321	0.857093
C	-2.645433	-1.863365	1.868121
C	-6.321868	-1.660004	-1.471895
C	-5.822998	0.749722	-1.941833
C	-4.960058	-2.792177	1.535743
C	-3.217014	-2.953745	-0.256220
C	-6.766121	0.211934	1.449551
C	-4.646857	0.552174	2.736473
H	4.370855	-0.548586	3.864681
H	4.242444	-4.626913	2.474496
H	4.006409	-4.779993	-2.584994
H	4.084150	-0.768495	-4.172014
H	3.305806	-6.025289	-0.858995
H	3.378204	-5.973416	0.906900
H	4.695777	-5.253959	-0.053794
H	1.025570	-4.972162	-0.808409
H	0.744745	-3.437197	0.049032
H	1.094307	-4.916498	0.975484
H	4.859824	-2.936984	4.166490
H	4.468793	-3.187876	-4.419180
H	-5.152922	1.271825	3.397261
H	-4.751641	-0.438635	3.189834
H	-3.582887	0.821270	2.703118
H	-5.717132	2.724567	1.738138
H	-4.180865	2.472152	0.872267
H	-5.725185	2.360293	0.004735
H	-7.254986	0.816615	2.229070
H	-7.317141	0.377774	0.517140
H	-6.872910	-0.841441	1.731839
H	-6.220652	0.643319	-2.962175
H	-6.652036	1.058944	-1.297298
H	-5.066499	1.545340	-1.960335
H	-6.847913	-1.667943	-2.439072
H	-5.926366	-2.668944	-1.309498
H	-7.063824	-1.450401	-0.693232
H	-4.743638	-0.964946	-3.600453
H	-3.401547	-0.215653	-2.700236
H	-3.749523	-1.946571	-2.511956
H	-2.798621	-3.855229	0.215994
H	-3.973300	-3.282281	-0.976352
H	-2.403182	-2.449763	-0.794829
H	-4.605941	-3.778117	1.874540

H	-5.326589	-2.258137	2.419600
H	-5.804564	-2.958066	0.857872
H	-2.297277	-2.858019	2.186305
H	-1.800221	-1.333705	1.406338
H	-2.943590	-1.313640	2.763786
C	3.450524	2.827793	1.705611
C	3.504504	4.024860	2.414347
C	3.278150	4.037023	3.792459
C	2.978816	2.837689	4.442830
C	2.906006	1.638154	3.736741
H	3.661769	2.826245	0.631033
H	3.736623	4.950714	1.887064
H	3.329799	4.970883	4.352026
H	2.781532	2.834743	5.515699
H	2.622629	0.723029	4.253628
C	2.958692	2.714604	-2.435486
C	3.565191	3.859099	-2.951835
C	4.857617	3.803807	-3.476248
C	5.545313	2.587345	-3.462606
C	4.955773	1.440478	-2.934682
H	1.934997	2.763116	-2.052611
H	3.011403	4.798923	-2.954230
H	5.326979	4.697497	-3.887828
H	6.562470	2.531250	-3.853258
H	5.521350	0.510711	-2.893933

PC

97

C	-3.223603	-2.310300	-0.483759
C	-4.082171	-1.691308	0.437039
C	-5.275923	-2.181708	0.930114
C	-5.606635	-3.488630	0.538946
C	-4.747382	-4.190363	-0.314564
C	-3.577767	-3.628499	-0.833093
C	-6.106924	-1.255017	1.829084
C	-5.989013	0.160459	1.246667
C	-4.761354	0.544206	0.743311
O	-3.691893	-0.373793	0.754172
C	-4.450653	1.735098	0.071397
C	-5.500220	2.672098	0.006806
C	-6.746294	2.352860	0.553767
C	-7.012435	1.116634	1.152901
N	-3.188720	1.782914	-0.510693
C	-2.738436	2.979991	-1.116049
AL	-2.098511	0.252701	-0.292405
O	-0.829659	0.255343	1.119298
C	0.087747	0.551405	0.235285
AU	2.103775	0.356685	0.493773
P	4.393116	-0.104335	0.554358
C	5.393024	1.516580	0.355956
C	4.737702	2.598445	1.237713
C	-7.564982	-1.709509	1.914564

C	-5.489246	-1.259638	3.250407
N	-2.202804	-1.514770	-0.987365
C	-1.144805	-2.103969	-1.717834
O	-0.401642	0.801789	-0.941395
C	4.843944	-0.956951	2.208087
C	3.712058	-1.940276	2.567842
C	4.750297	-1.285525	-0.913125
C	3.955059	-0.799260	-2.142195
C	6.190510	-1.692034	2.200898
C	4.852989	0.099988	3.325083
C	6.235112	-1.414390	-1.276000
C	4.186630	-2.676511	-0.575862
C	6.882703	1.394813	0.702363
C	5.248619	2.009987	-1.093483
H	-2.961980	-4.189054	-1.534607
H	-6.527763	-3.953787	0.884377
H	-8.009529	0.899650	1.530880
H	-5.350717	3.625274	-0.496875
H	-8.134169	-1.040837	2.572439
H	-7.625998	-2.717442	2.343581
H	-8.043211	-1.719458	0.926725
H	-6.048909	-0.576680	3.903915
H	-4.440660	-0.937943	3.230257
H	-5.533801	-2.272227	3.673848
H	-5.011145	-5.207352	-0.608320
H	-7.547987	3.089630	0.486054
H	4.938711	-0.427662	4.286646
H	5.698982	0.790956	3.254005
H	3.918769	0.676609	3.344462
H	3.916050	-2.340171	3.572694
H	2.739706	-1.429380	2.593825
H	3.634392	-2.786440	1.881609
H	6.372975	-2.092167	3.210065
H	6.199308	-2.540940	1.508595
H	7.028313	-1.032131	1.949594
H	5.229884	3.558471	1.020501
H	3.668882	2.702811	1.004693
H	4.837724	2.408309	2.308669
H	5.675602	3.022330	-1.148975
H	5.788093	1.386173	-1.813220
H	4.194688	2.075332	-1.395001
H	7.367900	2.361312	0.496879
H	7.047784	1.167564	1.761298
H	7.392761	0.633658	0.101652
H	4.087804	-1.542598	-2.942697
H	2.880786	-0.733381	-1.923624
H	4.290439	0.166991	-2.525361
H	6.330528	-2.156055	-2.083897
H	6.656684	-0.474073	-1.647970
H	6.847288	-1.759371	-0.435358
H	4.246882	-3.291615	-1.486055
H	4.752879	-3.191285	0.206761
H	3.128752	-2.624669	-0.286776
C	-2.771872	4.209084	-0.435762

C	-2.288867	5.367114	-1.041503
C	-1.752282	5.321794	-2.331210
C	-1.704837	4.101676	-3.008063
C	-2.198568	2.943436	-2.410213
H	-3.167851	4.245027	0.579419
H	-2.321117	6.311233	-0.495628
H	-1.373819	6.229065	-2.802821
H	-1.288019	4.050484	-4.014842
H	-2.169891	1.993127	-2.944688
C	-0.730095	-1.530281	-2.929146
C	0.364597	-2.043178	-3.622644
C	1.059344	-3.149000	-3.128894
C	0.649959	-3.730780	-1.926438
C	-0.436883	-3.213636	-1.223958
H	-1.272289	-0.666635	-3.315450
H	0.673417	-1.576055	-4.558699
H	1.914156	-3.551143	-3.673263
H	1.190930	-4.587993	-1.522366
H	-0.736701	-3.655008	-0.273089

[$^{t}Bu_3$PAuOAl(NON')] + CO

97

O	-0.128175626	1.488646721	-0.359133292
C	-2.343690025	-1.868385881	-1.199930513
C	-2.996501008	-1.566601787	0.006630659
C	-3.940234253	-2.330819697	0.667644392
C	-4.205815670	-3.590561034	0.111663956
C	-3.550251482	-3.973885768	-1.063436713
C	-2.644594173	-3.142011508	-1.726282427
C	-4.624670618	-1.706635110	1.893421814
C	-4.913332634	-0.244910960	1.524385514
C	-3.941003430	0.432290800	0.814777646
O	-2.744505496	-0.253464821	0.477498122
C	-4.045720171	1.700220092	0.221689372
C	-5.238571026	2.392225552	0.517499639
C	-6.222335554	1.776501745	1.294423538
C	-6.091278516	0.471141359	1.783608518
N	-3.013257278	2.049947908	-0.636272364
C	-2.854430322	3.369744667	-1.102740353
AL	-1.608940039	0.785349876	-0.754700414
AU	1.617904641	0.566295035	-0.035143920
P	3.612527560	-0.364255121	0.401757451
C	4.875851270	1.031609102	0.782893375
C	4.186719423	2.091606051	1.665380071
C	-5.904144260	-2.454770233	2.269942080
C	-3.644474277	-1.745091983	3.092874193
N	-1.582647887	-0.838159023	-1.743650388
C	-0.670726000	-1.036426884	-2.790589778
C	3.457966887	-1.521436407	1.925261837
C	2.149182276	-2.327235587	1.799862800
C	4.209374267	-1.373563332	-1.115440737
C	3.866617526	-0.579445169	-2.391234547

C	4.637581021	-2.482786576	2.123864823
C	3.299726728	-0.655674431	3.186493966
C	5.703517094	-1.721775242	-1.108138100
C	3.390684129	-2.672887780	-1.195378812
C	6.170862267	0.565099315	1.461595624
C	5.226188190	1.748417312	-0.532590779
H	-2.212258094	-3.459448935	-2.672191229
H	-4.931455508	-4.257338724	0.573330459
H	-6.905647074	0.014701322	2.342606624
H	-5.409057124	3.384905121	0.105216467
H	-6.370241756	-1.990958052	3.148321645
H	-5.674211438	-3.494958993	2.532369154
H	-6.630757672	-2.453280892	1.447338610
H	-4.098934573	-1.251027947	3.962304056
H	-2.701375969	-1.237040831	2.857929033
H	-3.417844637	-2.787148120	3.355740497
H	-3.772843452	-4.949058696	-1.498532656
H	-7.141775041	2.326305684	1.500209765
H	3.024054443	-1.318811410	4.019942986
H	4.222602220	-0.138763304	3.469080270
H	2.496952172	0.083543637	3.068276319
H	2.018795776	-2.904168108	2.728457173
H	1.284953805	-1.660232945	1.686267986
H	2.151601735	-3.033142118	0.966360509
H	4.473738022	-3.042856032	3.057393130
H	4.718174444	-3.216970902	1.314705025
H	5.596918342	-1.961860167	2.215471391
H	4.876471933	2.942375739	1.774824281
H	3.263694858	2.454525740	1.193763156
H	3.940240061	1.732214496	2.667099180
H	5.802101170	2.651838399	-0.281852184
H	5.843813958	1.139970753	-1.200824294
H	4.322548737	2.065873774	-1.069452517
H	6.840691116	1.432830705	1.563115244
H	5.995219743	0.172528818	2.469446370
H	6.702679460	-0.196437049	0.881109615
H	4.112596706	-1.209468764	-3.259270318
H	2.794733932	-0.348490448	-2.437484218
H	4.426178924	0.354258599	-2.485482264
H	5.915079023	-2.353362306	-1.984674105
H	6.340545552	-0.833970875	-1.187898649
H	6.002949771	-2.283000525	-0.215960109
H	3.611236408	-3.149984963	-2.161908611
H	3.646910455	-3.388608400	-0.407685587
H	2.313121056	-2.468748165	-1.168647750
C	-3.014652408	4.491620979	-0.270559250
C	-2.778418466	5.774046243	-0.760142636
C	-2.368633574	5.969623770	-2.082261982
C	-2.201754453	4.860480151	-2.913579249
C	-2.449868200	3.576476376	-2.431782448
H	-3.298540169	4.349138902	0.771730591
H	-2.900044300	6.630130885	-0.094812226
H	-2.180689794	6.975310100	-2.458526030
H	-1.886384744	4.994376905	-3.949208293

H	-2.347158266	2.713946187	-3.093198151
C	-0.392784962	0.044372431	-3.649128881
C	0.535962393	-0.069998639	-4.678536324
C	1.209263783	-1.275128933	-4.892512664
C	0.957498599	-2.348533933	-4.036742315
C	0.041810631	-2.234133592	-2.992764193
H	-0.921664488	0.989110832	-3.503732071
H	0.726846812	0.787815320	-5.324518007
H	1.928511532	-1.371075528	-5.705770233
H	1.495629575	-3.288407252	-4.169171735
H	-0.104195289	-3.070391145	-2.311621400
C	-0.442582871	0.412156281	2.643171495
O	-0.602527225	1.445604835	3.090888824

[$^{t}Bu_3PAu$] radical

41

C	0.575699	-0.398055	2.773312
C	1.456603	-0.861362	1.595706
C	2.934748	-0.612324	1.925007
P	0.826143	-0.000341	0.000716
C	1.456005	1.811473	-0.052312
C	2.935633	1.973280	-0.426742
AU	-1.551047	0.001349	0.004687
C	1.451495	-0.952014	-1.544339
C	2.928084	-1.367825	-1.495942
C	1.216122	-2.374231	1.460457
C	0.563487	-2.198275	-1.729923
C	1.214522	-0.077201	-2.786457
C	0.578398	2.597340	-1.046776
C	1.209883	2.451875	1.323658
H	0.838856	-1.003654	3.654084
H	0.719485	0.652518	3.036285
H	-0.489261	-0.554427	2.550925
H	1.413318	-2.836802	2.439249
H	0.174418	-2.592488	1.190515
H	1.881767	-2.849634	0.732910
H	3.196066	-1.198321	2.820616
H	3.603827	-0.926356	1.116085
H	3.139114	0.439245	2.154894
H	1.402324	3.531490	1.233025
H	0.168231	2.322811	1.646451
H	1.876291	2.063278	2.100397
H	0.834921	3.664059	-0.957915
H	0.731592	2.303232	-2.087585
H	-0.487746	2.476690	-0.808292
H	3.194538	3.042518	-0.369195
H	3.603054	1.432914	0.253723
H	3.144756	1.643682	-1.450564
H	3.185118	-1.852804	-2.451106

H	3.601064	-0.512914	-1.366151
H	3.131430	-2.092442	-0.699667
H	0.822898	-2.662576	-2.693745
H	0.702232	-2.950428	-0.949780
H	-0.499666	-1.920520	-1.753976
H	1.403069	-0.696412	-3.676394
H	0.176050	0.275803	-2.838183
H	1.888950	0.783628	-2.836978

$[^tBu_3PAu]^+$

41

C	-0.590111	2.787784	-0.302616
C	-1.476114	1.638717	-0.818081
C	-2.955635	1.946612	-0.531770
P	-0.940101	-0.000528	-0.000252
C	-1.479114	-0.110264	1.828274
C	-2.961364	-0.502818	1.952368
AU	1.318804	-0.001917	0.002410
C	-1.476633	-1.528422	-1.011869
C	-2.956135	-1.434751	-1.422027
C	-1.251339	1.555624	-2.335856
C	-0.588854	-1.656283	-2.263323
C	-1.253793	-2.801179	-0.180345
C	-0.600702	-1.137099	2.565781
C	-1.244212	1.244494	2.514433
H	-0.899674	3.701459	-0.829967
H	-0.686152	2.972822	0.768860
H	0.468766	2.608023	-0.534159
H	-1.457794	2.552574	-2.750096
H	-0.211695	1.304501	-2.582285
H	-1.922827	0.849874	-2.833619
H	-3.214392	2.861691	-1.083644
H	-3.628647	1.153509	-0.873989
H	-3.148322	2.140810	0.528101
H	-1.446738	1.106382	3.585843
H	-0.203049	1.577892	2.415714
H	-1.912519	2.032408	2.154748
H	-0.906967	-1.132426	3.621624
H	-0.707054	-2.157477	2.193025
H	0.460785	-0.856744	2.522886
H	-3.220525	-0.476918	3.020558
H	-3.630839	0.191192	1.433756
H	-3.158869	-1.518295	1.594304
H	-3.215759	-2.372007	-1.934806
H	-3.629181	-1.331297	-0.564508
H	-3.148705	-0.616466	-2.123165
H	-0.900811	-2.566362	-2.795530
H	-0.678681	-0.817929	-2.956531
H	0.468747	-1.773396	-1.989509
H	-1.455455	-3.658787	-0.837603
H	-0.215830	-2.888023	0.166044
H	-1.930201	-2.880335	0.675850

[$^{t}Bu_3PAu]^-$

41

C	-0.484889	2.692588	-0.190545
C	-1.421770	1.582404	-0.710897
C	-2.887762	1.972680	-0.484026
P	-0.795490	-0.051131	0.117128
C	-1.644953	-0.157317	1.860358
C	-3.135733	-0.511784	1.923342
AU	2.555779	1.076583	-2.565226
C	-1.489034	-1.517660	-0.937024
C	-2.938081	-1.425429	-1.432690
C	-1.159164	1.521068	-2.224242
C	-0.535026	-1.657240	-2.140998
C	-1.332095	-2.816906	-0.129538
C	-0.841277	-1.199145	2.666213
C	-1.419292	1.176797	2.592297
H	-0.735857	3.635912	-0.703271
H	-0.567627	2.865549	0.886923
H	0.558845	2.444218	-0.433313
H	-1.326086	2.529519	-2.637956
H	-0.109885	1.250763	-2.440927
H	-1.834305	0.836551	-2.750263
H	-3.097348	2.905447	-1.034886
H	-3.579759	1.206435	-0.854879
H	-3.121176	2.160697	0.570448
H	-1.706223	1.048960	3.649158
H	-0.361240	1.471476	2.567037
H	-2.017760	1.998040	2.184824
H	-1.169453	-1.167826	3.718712
H	-0.979019	-2.222559	2.306208
H	0.232559	-0.970008	2.634835
H	-3.470506	-0.498402	2.975354
H	-3.756059	0.200165	1.367183
H	-3.336781	-1.516284	1.532398
H	-3.184558	-2.338177	-2.002282
H	-3.656013	-1.344213	-0.608023
H	-3.091509	-0.573033	-2.103749
H	-0.745624	-2.610939	-2.653033
H	-0.643824	-0.851974	-2.871352
H	0.515454	-1.654559	-1.820217
H	-1.509316	-3.669949	-0.804457
H	-0.315812	-2.918715	0.275492
H	-2.050014	-2.895956	0.694914

[$^{t}Bu_3PAuCO_2$] radical C-coordination

44

AU	-1.058832	0.073615	-0.016406

P	1.230229	-0.338710	0.064408
C	1.544503	-1.762738	1.304337
C	0.645733	-1.546277	2.537357
C	-3.200607	0.475301	-0.115822
O	-3.736524	-0.323572	-0.882341
O	-3.468185	1.428976	0.614511
C	1.814890	-0.836156	-1.689990
C	0.768997	-1.785197	-2.307626
C	2.113774	1.260388	0.637947
C	1.457617	2.463995	-0.067448
C	1.831678	0.416254	-2.582237
C	3.199891	-1.497895	-1.721468
C	1.856806	1.458500	2.141071
C	3.626520	1.265745	0.376755
C	1.084025	-3.085732	0.670032
C	3.009259	-1.893311	1.745370
H	0.753057	-2.424183	3.191851
H	0.910133	-0.661586	3.120462
H	-0.410783	-1.464429	2.247003
H	1.113403	-3.859183	1.451637
H	0.050628	-3.020948	0.304737
H	1.733213	-3.418944	-0.145843
H	3.093345	-2.771693	2.403242
H	3.692163	-2.044132	0.902206
H	3.351591	-1.024807	2.318557
H	2.226575	2.457909	2.413950
H	0.785018	1.424823	2.376583
H	2.385773	0.731792	2.765816
H	1.900258	3.380816	0.349953
H	1.617661	2.478593	-1.147609
H	0.375706	2.491095	0.121981
H	4.044419	2.194105	0.794720
H	4.140227	0.425610	0.857069
H	3.865997	1.254291	-0.692109
H	3.466521	-1.688236	-2.772209
H	3.981260	-0.861686	-1.291483
H	3.213687	-2.463075	-1.203404
H	1.060227	-1.973161	-3.351901
H	0.702103	-2.750645	-1.801697
H	-0.229503	-1.327064	-2.308826
H	2.007756	0.086560	-3.616766
H	0.868282	0.942440	-2.560684
H	2.630586	1.117424	-2.321205

[$^{t}Bu_3PAuCO_2$] radical O-coordination

44

C	-1.133571	-0.770727	2.798207
C	-1.917538	0.144564	1.837146
C	-3.421836	-0.133575	1.969602
P	-1.231680	-0.096817	0.064360
C	-1.900764	-1.725893	-0.688778
C	-3.356961	-1.639924	-1.168485

AU	1.031111	-0.209721	0.155624
C	3.872741	0.521788	-0.285972
O	3.162162	-0.374589	0.259109
C	-1.707316	1.391533	-1.041779
C	-3.172595	1.828652	-0.902898
C	-1.629013	1.585035	2.292041
O	3.677662	1.546557	-0.904435
C	-0.775633	2.572927	-0.706827
C	-1.424186	1.033250	-2.510029
C	-0.991174	-2.139355	-1.862091
C	-1.777916	-2.845833	0.357481
H	-1.464167	-0.544763	3.823086
H	-1.300577	-1.835515	2.621182
H	-0.054427	-0.575596	2.735932
H	-1.879580	1.654220	3.360928
H	-0.566947	1.840056	2.181646
H	-2.233331	2.329977	1.765019
H	-3.723222	0.094822	3.003130
H	-4.027056	0.490656	1.303197
H	-3.671602	-1.183903	1.783965
H	-2.008304	-3.798110	-0.142506
H	-0.757114	-2.917126	0.755510
H	-2.480515	-2.734085	1.189099
H	-1.330199	-3.123297	-2.219810
H	-1.024482	-1.447789	-2.706623
H	0.053485	-2.235662	-1.536815
H	-3.662762	-2.637570	-1.518668
H	-4.047574	-1.342977	-0.371839
H	-3.479617	-0.949456	-2.009921
H	-3.357317	2.640515	-1.622470
H	-3.877978	1.021067	-1.126976
H	-3.399467	2.221260	0.094238
H	-0.998880	3.386636	-1.413059
H	-0.910620	2.963250	0.304249
H	0.279890	2.294188	-0.831335
H	-1.546658	1.949511	-3.106136
H	-0.393470	0.681972	-2.649669
H	-2.116963	0.286534	-2.910692

$[^tBu_3PAuCO_2]^+$

44

AU	0.948603	-0.128294	0.012217
P	-1.309732	-0.058627	0.003330
C	-1.875111	0.209570	1.807897
C	-1.030244	-0.675870	2.743173
C	4.074229	0.625389	-0.058635
O	4.924096	1.410634	-0.112211
O	3.224537	-0.193868	0.001836
C	-1.814801	1.398707	-1.122420
C	-0.896915	2.603162	-0.840929
C	-1.919684	-1.731698	-0.689588
C	-1.037919	-2.139355	-1.884605

C	-1.587064	1.013396	-2.592891
C	-3.284000	1.803207	-0.921744
C	-1.741978	-2.824941	0.376167
C	-3.393886	-1.675549	-1.121312
C	-1.602399	1.665124	2.219240
C	-3.367471	-0.104295	1.999130
H	-1.340858	-0.457817	3.775149
H	-1.163516	-1.746330	2.574986
H	0.039204	-0.438932	2.657168
H	-1.812700	1.746625	3.295158
H	-0.551770	1.944319	2.066146
H	-2.246298	2.385204	1.705551
H	-3.630290	0.139549	3.038503
H	-4.013932	0.492686	1.347304
H	-3.597051	-1.163862	1.846367
H	-1.975248	-3.787509	-0.100882
H	-0.707579	-2.879365	0.739762
H	-2.419203	-2.710755	1.227687
H	-1.384849	-3.123862	-2.230567
H	-1.094621	-1.451109	-2.730084
H	0.014544	-2.241185	-1.586073
H	-3.687989	-2.686492	-1.438634
H	-4.063072	-1.378459	-0.307025
H	-3.558329	-1.007543	-1.972976
H	-3.515960	2.592566	-1.651147
H	-3.979708	0.976339	-1.098517
H	-3.474408	2.215027	0.074702
H	-1.183884	3.406973	-1.534063
H	-0.980939	2.993995	0.174928
H	0.155464	2.353366	-1.033791
H	-1.760705	1.914407	-3.198235
H	-0.554976	0.686611	-2.775189
H	-2.277357	0.242768	-2.948542

$[^tBu_3PAuCO_2]^-$

44

AU	1.198177	0.001534	0.000715
P	-1.199357	-0.006254	-0.001771
C	-1.868186	0.235974	1.785018
C	-0.991320	-0.615349	2.727111
C	3.291780	0.033085	-0.016320
O	3.837412	1.169962	0.087888
O	3.875101	-1.083812	-0.133144
C	-1.852241	1.428094	-1.105767
C	-0.971211	2.665439	-0.834609
C	-1.871288	-1.671323	-0.694000
C	-0.981668	-2.067695	-1.890437
C	-1.594436	1.053374	-2.575115
C	-3.330057	1.799522	-0.933897
C	-1.641979	-2.760297	0.367723
C	-3.345492	-1.686750	-1.117048
C	-1.619971	1.696954	2.197005

C	-3.347104	-0.102226	2.007889
H	-1.237122	-0.346171	3.766546
H	-1.152036	-1.690491	2.613322
H	0.075230	-0.410510	2.555584
H	-1.810143	1.783848	3.278212
H	-0.578396	1.992173	2.013305
H	-2.286439	2.401380	1.688017
H	-3.609121	0.125112	3.054474
H	-4.012437	0.484081	1.363427
H	-3.559419	-1.164966	1.843738
H	-1.836512	-3.738471	-0.099003
H	-0.602402	-2.758407	0.721893
H	-2.313811	-2.666663	1.227496
H	-1.230439	-3.101196	-2.179222
H	-1.127379	-1.431666	-2.767277
H	0.081492	-2.028175	-1.613171
H	-3.609130	-2.705963	-1.445157
H	-4.020096	-1.416352	-0.296392
H	-3.542274	-1.014158	-1.959777
H	-3.581391	2.594741	-1.655175
H	-4.001003	0.953829	-1.123487
H	-3.544875	2.190650	0.067409
H	-1.201192	3.426931	-1.596604
H	-1.145538	3.113259	0.146999
H	0.095189	2.407567	-0.906030
H	-1.778237	1.946111	-3.192972
H	-0.551414	0.746375	-2.730561
H	-2.257821	0.260371	-2.936171

[(NON')Al] radical

53

C	1.733232	3.965297	-0.541228
C	1.794165	2.660485	-0.019953
C	3.005341	2.219606	0.541400
C	4.122121	3.053778	0.579406
C	4.048167	4.352200	0.073441
C	2.847177	4.799851	-0.484733
N	0.724360	1.748845	-0.112710
C	-0.598161	2.135041	0.092935
C	-1.564681	1.176356	-0.242325
C	-2.919561	1.223533	0.025868
C	-3.381061	2.406781	0.621188
C	-2.473511	3.427926	0.922945
C	-1.102762	3.311313	0.679949
C	-3.759038	-0.013043	-0.322461
C	-2.910491	-1.240081	0.037853
C	-1.556181	-1.186480	-0.232485
O	-1.011257	-0.005416	-0.789667
C	-3.363437	-2.420866	0.644494
C	-2.448958	-3.433566	0.953677
C	-1.079514	-3.310931	0.706281
C	-0.583236	-2.137157	0.107242

N	0.736447	-1.745731	-0.107225
AL	0.980784	0.001415	-0.832102
C	-5.096057	-0.014192	0.419905
C	-4.026552	-0.021661	-1.848949
C	1.811512	-2.651623	-0.025305
C	3.031801	-2.197984	0.506026
C	4.154329	-3.024740	0.528868
C	4.077670	-4.329385	0.039470
C	2.867930	-4.789970	-0.488493
C	1.747857	-3.962709	-0.530551
H	-0.424607	4.107118	0.981114
H	-4.434818	2.528424	0.863773
H	-4.416031	-2.546974	0.889889
H	-0.396037	-4.100011	1.013081
H	-5.681715	-0.901176	0.148187
H	-5.688688	0.865058	0.138286
H	-4.954442	-0.007562	1.508218
H	-4.598358	-0.918491	-2.122960
H	-3.089973	-0.020635	-2.420062
H	-4.605585	0.867781	-2.131726
H	-2.842672	4.341842	1.389804
H	-2.811473	-4.345470	1.429591
H	3.055072	1.217403	0.972584
H	5.050574	2.689296	1.020396
H	4.916776	5.009458	0.111292
H	2.780434	5.807645	-0.897031
H	0.815126	4.312921	-1.013815
H	0.822664	-4.321415	-0.980331
H	2.798518	-5.802546	-0.888501
H	4.950814	-4.981142	0.066496
H	5.089903	-2.649455	0.945016
H	3.085164	-1.190397	0.924132

[(NON')Al]$^{+}$

53

C	1.740411	-3.974753	0.652354
C	1.789543	-2.716088	0.040558
C	2.959399	-2.311488	-0.614245
C	4.075445	-3.146171	-0.648718
C	4.021975	-4.405321	-0.049481
C	2.852462	-4.813903	0.594836
N	0.706942	-1.800090	0.122674
C	-0.650927	-2.177097	-0.064252
C	-1.600573	-1.181442	0.197637
C	-2.952771	-1.196877	-0.070659
C	-3.427317	-2.410467	-0.590411
C	-2.541755	-3.470131	-0.813737
C	-1.166374	-3.375208	-0.574356
C	-3.775748	0.066790	0.233486
C	-2.908772	1.300378	-0.071700
C	-1.557901	1.236983	0.196469
O	-1.010169	0.017904	0.683637

C	-3.339445	2.529820	-0.592339
C	-2.416692	3.557522	-0.815370
C	-1.045471	3.413519	-0.576066
C	-0.573298	2.197524	-0.066740
N	0.770494	1.772405	0.118216
AL	0.808052	-0.014447	0.359208
C	-5.077236	0.088960	-0.570676
C	-4.105916	0.073175	1.748452
C	1.883548	2.650738	0.028961
C	3.029038	2.213171	-0.647014
C	4.173606	3.008369	-0.685766
C	4.172778	4.260829	-0.070570
C	3.026396	4.703360	0.593033
C	1.886149	3.903350	0.654814
H	-0.505749	-4.205644	-0.813259
H	-4.481331	-2.531678	-0.831145
H	-4.388287	2.688473	-0.833958
H	-0.355245	4.219875	-0.813998
H	-5.663964	0.980865	-0.320783
H	-5.695422	-0.780711	-0.318246
H	-4.886534	0.083913	-1.651134
H	-4.676000	0.976317	2.002160
H	-3.193942	0.057265	2.358981
H	-4.707730	-0.808962	2.002790
H	-2.931681	-4.405182	-1.215154
H	-2.773145	4.505809	-1.217051
H	2.983281	-1.345416	-1.129419
H	4.980007	-2.818487	-1.160779
H	4.887800	-5.065601	-0.084676
H	2.807532	-5.791781	1.074324
H	0.844377	-4.285325	1.188304
H	1.007720	4.238754	1.204957
H	3.022274	5.676387	1.084367
H	5.061436	4.889975	-0.108380
H	5.059400	2.655066	-1.213368
H	3.011851	1.253355	-1.173494

[(NON')Al]$^-$

53

C	1.634184	-4.023992	0.390725
C	1.727392	-2.670795	-0.008159
C	2.994822	-2.205665	-0.429419
C	4.103074	-3.049896	-0.463964
C	3.988003	-4.390389	-0.089091
C	2.743064	-4.864394	0.342637
N	0.693866	-1.746732	0.101911
C	-0.621245	-2.096364	-0.109028
C	-1.585583	-1.138151	0.264425
C	-2.940818	-1.174739	-0.015400
C	-3.423884	-2.323827	-0.660827
C	-2.523123	-3.337144	-1.007495
C	-1.153813	-3.239711	-0.753773

C	-3.760201	0.066587	0.362937
C	-2.897968	1.276936	-0.020559
C	-1.544717	1.194284	0.259706
O	-1.047031	0.020148	0.847504
C	-3.340809	2.439808	-0.670225
C	-2.405021	3.419183	-1.021826
C	-1.039842	3.274800	-0.767900
C	-0.547482	2.116894	-0.117224
N	0.754788	1.723233	0.096878
AL	1.101091	-0.017202	1.181506
C	-5.115859	0.088528	-0.343672
C	-3.993599	0.074090	1.894770
C	1.820215	2.610238	-0.011774
C	3.074235	2.096967	-0.417416
C	4.212549	2.900399	-0.445910
C	4.142488	4.246669	-0.081163
C	2.911017	4.768662	0.333310
C	1.771952	3.969206	0.375410
H	-0.484880	-4.025335	-1.099979
H	-4.479523	-2.421518	-0.907740
H	-4.392489	2.573662	-0.917384
H	-0.343986	4.034747	-1.118370
H	-5.681492	0.983847	-0.054745
H	-5.712035	-0.785543	-0.051014
H	-5.002356	0.084230	-1.435558
H	-4.547242	0.978151	2.184701
H	-3.043296	0.059177	2.441710
H	-4.578037	-0.808989	2.188884
H	-2.896795	-4.226742	-1.518409
H	-2.747249	4.319068	-1.536739
H	3.086309	-1.167916	-0.752354
H	5.065220	-2.655485	-0.796051
H	4.852393	-5.054166	-0.124894
H	2.637900	-5.903330	0.662095
H	0.687018	-4.403506	0.772526
H	0.835597	4.386018	0.744562
H	2.839875	5.813153	0.643941
H	5.030880	4.878305	-0.112015
H	5.163068	2.468989	-0.765180
H	3.131901	1.054132	-0.731605

[(NON')AlCO_2] radical C-O1-coordination

56

C	2.579899	-2.505467	0.899701
C	1.351147	-2.794601	0.284614
C	1.170305	-4.058800	-0.302226
C	2.195890	-5.000253	-0.270520
C	3.421500	-4.700668	0.331950
C	3.608856	-3.446220	0.912621
N	0.371629	-1.780232	0.201692
AL	0.743084	-0.068136	-0.439320
C	-0.983238	-2.043096	0.401672

C	-1.856901	-1.006127	0.046596
C	-3.211016	-0.922895	0.303582
C	-3.782034	-2.056725	0.900920
C	-2.973988	-3.154986	1.215684
C	-1.595497	-3.165004	0.989102
O	-1.191474	0.117065	-0.512981
C	-1.630616	1.353307	0.032502
C	-2.975741	1.532556	0.289162
C	-3.930727	0.383051	-0.063067
C	-3.318935	2.761132	0.873723
C	-2.315477	3.688676	1.176446
C	-0.960564	3.432477	0.952116
C	-0.574582	2.207708	0.378345
N	0.704937	1.688904	0.184415
C	1.860207	2.497086	0.270505
C	3.002518	1.984590	0.906181
C	4.193001	2.710394	0.926428
C	4.257527	3.970603	0.331983
C	3.120272	4.492973	-0.291307
C	1.933018	3.765589	-0.329873
C	-5.265867	0.514430	0.671292
C	-4.187799	0.398022	-1.591465
C	2.683645	-0.243537	-1.503949
O	3.826486	-0.343470	-1.838852
O	1.529613	-0.151671	-2.104605
H	-0.206116	4.157629	1.251389
H	-4.356826	2.990931	1.105744
H	-4.844501	-2.080956	1.133734
H	-0.993199	-4.017410	1.297553
H	-5.934636	-0.309909	0.394548
H	-5.766549	1.448242	0.387185
H	-5.130796	0.506162	1.760362
H	-4.841301	-0.439997	-1.868117
H	-3.252252	0.308145	-2.157250
H	-4.676658	1.338618	-1.878303
H	-2.596078	4.639347	1.631077
H	-3.430819	-4.029156	1.680760
H	2.944705	1.011716	1.397580
H	5.070257	2.289779	1.418766
H	5.185545	4.541944	0.350972
H	3.162684	5.471965	-0.770276
H	1.062654	4.164665	-0.850182
H	0.231802	-4.287745	-0.806408
H	2.042145	-5.973102	-0.739035
H	4.222888	-5.439333	0.345637
H	4.557807	-3.197575	1.388480
H	2.717557	-1.535838	1.381508

[(NON')AlCO_2] radical C-O2-coordination

56

C	2.121261	3.619897	-0.305566
C	1.909805	2.358404	0.273263

C	2.931055	1.787314	1.045820
C	4.140250	2.455723	1.231038
C	4.341692	3.713629	0.661102
C	3.325884	4.290438	-0.106024
N	0.722157	1.621066	0.047423
C	-0.531134	2.212936	0.207057
C	-1.621584	1.424790	-0.184775
C	-2.962830	1.681163	0.019299
C	-3.255144	2.919712	0.610424
C	-2.210890	3.781984	0.964902
C	-0.865179	3.451136	0.784456
C	-3.967441	0.596511	-0.396681
C	-3.342153	-0.759821	-0.038586
C	-1.984141	-0.913039	-0.235752
O	-1.232076	0.178302	-0.724442
C	-4.002184	-1.876791	0.495103
C	-3.271353	-3.028284	0.810068
C	-1.886042	-3.110917	0.645480
C	-1.186391	-2.008134	0.123260
N	0.189118	-1.813297	-0.007349
AL	0.702357	-0.123699	-0.616573
C	-5.320450	0.789950	0.288855
C	-4.159804	0.662713	-1.933729
C	1.102528	-2.875000	0.196694
C	2.245949	-2.647521	0.976567
C	3.204549	-3.646092	1.141456
C	3.027569	-4.894801	0.543734
C	1.887621	-5.129924	-0.229862
C	0.934167	-4.130005	-0.409957
H	-0.081908	4.127824	1.120570
H	-4.285634	3.207769	0.807940
H	-5.074043	-1.848262	0.680142
H	-1.348007	-4.005433	0.952910
H	-6.026015	0.015468	-0.036214
H	-5.752817	1.759567	0.012388
H	-5.229335	0.745344	1.381748
H	-4.849235	-0.128086	-2.258049
H	-3.208050	0.530055	-2.462959
H	-4.579799	1.637476	-2.215674
H	-2.452802	4.741075	1.423956
H	-3.797484	-3.888645	1.224923
H	2.764500	0.814502	1.512480
H	4.924055	1.994320	1.832500
H	5.284862	4.239497	0.809635
H	3.477996	5.266769	-0.567539
H	1.344090	4.060286	-0.929957
H	0.064240	-4.308581	-1.041607
H	1.745610	-6.097420	-0.712541
H	3.773809	-5.678259	0.675745
H	4.088607	-3.448573	1.748452
H	2.373793	-1.679404	1.465010
C	1.645600	-0.274389	-2.510809
O	2.374107	-0.365244	-1.438719
O	1.820633	-0.312360	-3.696318

$[(NON')AlCO_2]^+$

56

C	2.605094	-2.213305	0.914638
C	1.465589	-2.651069	0.220720
C	1.481962	-3.913942	-0.389124
C	2.621860	-4.711007	-0.305229
C	3.762611	-4.261695	0.365997
C	3.751794	-3.006768	0.974194
N	0.375078	-1.759801	0.087587
AL	0.543491	0.007526	-0.313174
C	-0.972834	-2.158908	0.200996
C	-1.913363	-1.187025	-0.160093
C	-3.280926	-1.224804	0.018182
C	-3.772135	-2.431533	0.538817
C	-2.889277	-3.469699	0.853507
C	-1.503058	-3.352473	0.708566
O	-1.311863	0.011804	-0.642420
C	-1.908080	1.213807	-0.161168
C	-3.275458	1.258029	0.016600
C	-4.102536	0.018211	-0.356733
C	-3.761261	2.467587	0.535758
C	-2.873699	3.501724	0.850468
C	-1.487917	3.377483	0.707029
C	-0.963236	2.180923	0.201011
N	0.382673	1.774543	0.090412
C	1.478213	2.658682	0.227116
C	2.611439	2.214260	0.927296
C	3.763870	2.999500	0.988835
C	3.786872	4.252008	0.376275
C	2.651959	4.708393	-0.300076
C	1.506303	3.919881	-0.385815
C	-5.460356	0.021622	0.348241
C	-4.320417	0.017724	-1.891588
C	3.331576	0.015007	-1.260989
O	4.477596	0.014619	-1.172075
O	2.149577	0.003803	-1.406548
H	-0.826250	4.183873	1.016851
H	-4.827031	2.602964	0.707372
H	-4.838405	-2.561563	0.711481
H	-0.845388	-4.162182	1.018241
H	-6.044721	-0.858425	0.054161
H	-6.040847	0.903904	0.053157
H	-5.349876	0.022006	1.439954
H	-4.888804	-0.873900	-2.186710
H	-3.366311	0.015299	-2.433767
H	-4.884846	0.911489	-2.187841
H	-3.272085	4.433750	1.250870
H	-3.291798	-4.399565	1.254768
H	2.564691	1.266868	1.474093
H	4.634170	2.639052	1.537491
H	4.679762	4.873737	0.431458

H	2.663314	5.685702	-0.783076
H	0.640068	4.268374	-0.947273
H	0.611066	-4.257461	-0.946382
H	2.623891	-5.689666	-0.785614
H	4.650597	-4.890463	0.420366
H	4.626972	-2.650789	1.517947
H	2.568819	-1.262810	1.456823

$[(NON')AlCO_2]^-$

56

C	2.696004	-2.310910	1.039495
C	1.512654	-2.688456	0.379422
C	1.449835	-3.977760	-0.182497
C	2.526631	-4.855245	-0.073621
C	3.696265	-4.470311	0.587243
C	3.772846	-3.188708	1.137863
N	0.480313	-1.744629	0.230505
AL	0.873045	-0.003654	-0.516006
C	-0.847626	-2.124946	0.302603
C	-1.778990	-1.165854	-0.130985
C	-3.153955	-1.218518	0.001274
C	-3.679230	-2.400220	0.547552
C	-2.805255	-3.419985	0.941127
C	-1.417374	-3.301229	0.837436
O	-1.179773	0.004548	-0.627846
C	-1.769675	1.179503	-0.130533
C	-3.144253	1.243161	0.001344
C	-3.951454	0.015492	-0.440411
C	-3.660143	2.429049	0.547499
C	-2.778003	3.441520	0.941730
C	-1.391091	3.311406	0.838904
C	-0.830714	2.130782	0.303740
N	0.494247	1.739994	0.231666
C	1.533801	2.675899	0.379906
C	2.714955	2.289518	1.038907
C	3.797952	3.159718	1.137145
C	3.730089	4.442254	0.587566
C	2.562860	4.835795	-0.072521
C	1.480070	3.965805	-0.181534
C	-5.359464	0.021059	0.156482
C	-4.055257	0.015965	-1.986322
C	2.527910	-0.010611	-1.534352
O	3.636110	-0.014937	-2.053517
O	1.344865	-0.004040	-2.282955
H	-0.740912	4.103465	1.205411
H	-4.733039	2.559950	0.676141
H	-4.753080	-2.522072	0.676893
H	-0.773366	-4.098542	1.203445
H	-5.920035	-0.860393	-0.180167
H	-5.912395	0.907733	-0.178996
H	-5.331689	0.020346	1.253881
H	-4.600780	-0.875648	-2.325214

H	-3.061963	0.011915	-2.451363
H	-4.593424	0.912048	-2.325240
H	-3.184245	4.360425	1.367689
H	-3.218879	-4.335503	1.367274
H	2.775806	1.285956	1.460937
H	4.705059	2.830219	1.645943
H	4.578918	5.122499	0.662534
H	2.500053	5.825826	-0.527673
H	0.592488	4.270842	-0.735261
H	0.560112	-4.276466	-0.736271
H	2.457237	-5.844465	-0.529579
H	4.540295	-5.156527	0.662005
H	4.681904	-2.865932	1.647480
H	2.763606	-1.308049	1.462147

[(NON')AlCO_2] radical TSII-like coordination

56

C	1.377940	3.006725	-1.744058
C	1.468151	2.432232	-0.464453
C	2.689064	2.506308	0.226166
C	3.793334	3.132498	-0.351533
C	3.695297	3.713102	-1.616310
C	2.481175	3.646442	-2.305036
N	0.406439	1.694604	0.115121
AL	0.621351	-0.027850	0.771154
N	0.591925	-1.663377	-0.105833
C	1.770986	-2.386557	-0.361156
C	2.921733	-1.655306	-0.707308
C	4.148257	-2.289928	-0.886065
C	4.247256	-3.675547	-0.745225
C	3.108691	-4.409568	-0.404605
C	1.881552	-3.778757	-0.206057
C	-0.936808	2.045510	-0.051782
C	-1.871892	1.053194	0.273809
C	-3.230669	1.060385	0.035231
C	-3.747630	2.268650	-0.455013
C	-2.882234	3.346508	-0.678024
C	-1.498857	3.255302	-0.500188
O	-1.284023	-0.143476	0.774125
C	-1.737240	-1.299503	0.074207
C	-3.084202	-1.391476	-0.212283
C	-3.996317	-0.251444	0.274075
C	-3.461273	-2.513372	-0.965437
C	-2.481165	-3.414227	-1.396347
C	-1.119229	-3.236723	-1.134413
C	-0.696895	-2.123720	-0.383242
C	-5.338132	-0.255506	-0.458922
C	-4.248132	-0.420472	1.794276
O	1.357947	-0.225366	2.355337
C	1.798264	0.453114	3.373439
O	2.663207	1.281337	3.473332
H	-0.390975	-3.929418	-1.549715

H	-4.503354	-2.675654	-1.233064
H	-4.809329	2.370231	-0.670485
H	-0.858328	4.104274	-0.730230
H	-5.975210	0.557184	-0.088852
H	-5.869497	-1.197090	-0.273138
H	-5.208579	-0.133467	-1.541940
H	-4.875323	0.403008	2.160410
H	-3.310031	-0.419366	2.362184
H	-4.765560	-1.370525	1.982514
H	-2.785225	-4.278882	-1.987211
H	-3.297426	4.290162	-1.033252
H	2.845301	-0.573189	-0.862009
H	5.025534	-1.699642	-1.151988
H	5.202646	-4.177701	-0.895515
H	3.177504	-5.490306	-0.273832
H	1.016229	-4.360536	0.106881
H	2.765156	2.089946	1.232707
H	4.731189	3.177938	0.203206
H	4.555125	4.212362	-2.063002
H	2.394168	4.083906	-3.300416
H	0.449647	2.928455	-2.308351

[(NON')AlCO_2] radical PC-like coordination

56

C	2.452358	3.452430	-0.354116
C	2.139487	2.228560	0.257323
C	3.086346	1.629066	1.099042
C	4.322809	2.234552	1.320361
C	4.625810	3.456289	0.716974
C	3.683853	4.061272	-0.119521
N	0.909542	1.567124	0.006158
AL	0.740043	-0.155433	-0.696867
O	2.604586	-0.559026	-1.254784
C	2.154959	-0.445332	-2.424822
O	-1.169844	0.268901	-0.720243
C	-1.452894	1.534152	-0.157161
C	-2.764900	1.875125	0.103951
C	-3.853263	0.857475	-0.268756
C	-3.300464	-0.536495	0.063175
C	-1.965429	-0.774887	-0.195432
C	-2.953439	3.126943	0.709363
C	-1.841568	3.915835	1.025111
C	-0.528023	3.496816	0.792668
C	-0.297738	2.241772	0.200358
C	-1.220039	-1.913979	0.136739
C	-1.964984	-2.970656	0.691137
C	-3.334583	-2.802330	0.916175
C	-4.006118	-1.608360	0.630485
C	-5.161101	1.134903	0.474011
C	-4.106482	0.940193	-1.795742
N	0.161213	-1.805766	-0.039605
C	0.998282	-2.925881	0.199257

C	0.772440	-4.159158	-0.431146
C	1.633207	-5.231878	-0.206600
C	2.737168	-5.090187	0.638114
C	2.971307	-3.862871	1.260388
C	2.104062	-2.791622	1.049930
O	0.944999	-0.176796	-2.666404
H	0.310377	4.118155	1.101677
H	-3.954592	3.481267	0.946242
H	-5.064966	-1.512227	0.862034
H	-1.470851	-3.896694	0.978589
H	-5.927617	0.406835	0.181038
H	-5.542554	2.130450	0.215176
H	-5.026592	1.083592	1.562134
H	-4.859476	0.196962	-2.090330
H	-3.188742	0.747440	-2.364858
H	-4.473765	1.941149	-2.059362
H	-2.001277	4.886912	1.495199
H	-3.893532	-3.628774	1.356670
H	-0.074339	-4.264626	-1.109613
H	1.446233	-6.183306	-0.706008
H	3.411325	-5.929861	0.807343
H	3.828231	-3.739328	1.923440
H	2.275673	-1.837902	1.551861
H	2.841613	0.683582	1.585798
H	5.048501	1.752551	1.976318
H	5.589920	3.932983	0.894389
H	3.914545	5.010174	-0.605262
H	1.728206	3.914420	-1.025568

[$^{t}Bu_{3}$CuAl(NON')]

94

C	-0.51899	2.06947	4.23329
C	-1.49119	1.11544	4.57379
C	-2.34116	1.14214	5.66352
C	-2.29555	2.30116	6.45237
C	-1.39743	3.32213	6.12091
C	-0.51131	3.22280	5.04603
C	-3.20011	-0.10479	5.91475
C	-2.29788	-1.31409	5.63025
C	-1.45181	-1.23083	4.53961
O	-1.45257	-0.04625	3.76790
C	-0.45536	-2.14754	4.16629
C	-0.40614	-3.31930	4.95076
C	-1.28184	-3.47349	6.02791
C	-2.21092	-2.49088	6.38870
N	0.38947	-1.72743	3.15069
C	1.23540	-2.60522	2.46280
Al	-0.00000	-0.00000	2.34444
C	-3.75351	-0.13365	7.33957
C	-4.38173	-0.11177	4.91245
N	0.34690	1.69969	3.21736
C	1.19779	2.61728	2.58709

H	0.21342	4.01363	4.86220
H	-2.93728	2.40280	7.32543
H	-2.84509	-2.63564	7.26121
H	0.34197	-4.08227	4.74444
H	-4.36645	-1.03091	7.49203
H	-4.39870	0.73641	7.51494
H	-2.94904	-0.12846	8.08625
H	-4.98541	-1.01875	5.05270
H	-4.02642	-0.08867	3.87514
H	-5.01900	0.76711	5.08031
H	-1.36495	4.21891	6.74137
H	-1.21643	-4.38373	6.62573
C	1.75124	-4.73206	1.39416
C	2.96037	-4.22476	0.90819
C	3.29933	-2.90090	1.19004
C	2.45324	-2.10392	1.95899
H	-0.05818	-4.34036	2.49217
H	1.46155	-5.75934	1.16689
H	3.62481	-4.85280	0.31466
H	4.23609	-2.48414	0.81751
H	2.74118	-1.08011	2.20319
C	2.46057	2.17046	2.15150
C	3.31497	3.00874	1.43736
C	2.93636	4.31973	1.14527
C	1.68273	4.77393	1.56780
C	0.82073	3.93836	2.27376
H	2.77445	1.15650	2.40472
H	4.28718	2.63409	1.11421
H	3.60645	4.97982	0.59435
H	1.36488	5.79120	1.33319
H	-0.16567	4.29717	2.56600
C	0.89591	-3.93818	2.15394
P	0.20407	-0.05571	-2.23163
C	-1.50696	-0.29263	-3.06607
C	-2.51317	0.58606	-2.29562
Cu	0.00000	0.00000	0.00000
H	0.43422	3.66639	-2.90634
H	-0.85728	2.60922	-3.49743
H	-0.53555	2.74082	-1.74493
H	2.42164	2.99577	-2.03061
H	1.66811	1.98950	-0.77746
H	2.94045	1.31583	-1.81345
H	1.95904	2.57764	-4.47835
H	2.42747	0.87855	-4.32448
H	0.84105	1.30380	-5.00163
H	-3.52691	0.35207	-2.65591
H	-2.47575	0.37101	-1.21737
H	-2.34972	1.65751	-2.43371
H	-3.02071	-1.81593	-3.16702
H	-1.39727	-2.46602	-3.44028
H	-1.91938	-2.02897	-1.79062
H	-2.57863	-0.17805	-4.93431
H	-1.35328	1.08773	-4.77023
H	-0.86380	-0.57773	-5.14756

H	1.69600	-3.51349	-1.93590
H	0.94456	-2.44208	-0.74073
H	-0.02999	-3.13441	-2.05677
H	2.06268	-2.74958	-4.31473
H	0.35869	-2.30863	-4.48898
H	1.62470	-1.11150	-4.83553
H	3.40300	-2.07498	-2.37388
H	3.23864	-0.39600	-2.91497
H	2.83728	-0.84306	-1.23091
C	0.98729	1.59194	-2.82621
C	2.07146	1.97794	-1.80013
C	1.35135	-1.52194	-2.69216
C	0.94766	-2.71728	-1.80531
C	1.58203	1.57064	-4.23930
C	-0.07223	2.70354	-2.74015
C	1.33756	-1.93322	-4.16952
C	2.78940	-1.16625	-2.27955
C	-1.56281	0.03223	-4.56386
C	-1.96926	-1.74313	-2.84996

[$^{t}Bu_3$AuAl(NON)]

154

AU	1.923298	0.001862	-0.477909
P	4.351415	-0.008106	-0.391239
AL	-0.475406	0.012266	-0.107464
O	-2.374816	-0.024529	-0.932813
N	-1.052268	-1.757807	0.375982
N	-1.102337	1.784487	0.308616
C	-3.101064	-1.191962	-0.636922
C	-2.347135	-2.138514	0.056452
C	-3.005962	-3.346170	0.353942
H	-2.446620	-4.124944	0.866225
C	-4.353415	-3.527873	0.016224
C	-5.068563	-2.486883	-0.606583
H	-6.125023	-2.619770	-0.830876
C	-4.443744	-1.279874	-0.946716
C	-5.096705	-0.070043	-1.632746
C	-4.462760	1.191150	-1.027299
C	-5.115583	2.406941	-0.757600
H	-6.173194	2.500063	-0.986061
C	-4.421830	3.489916	-0.196870
C	-3.066472	3.351439	0.150841
H	-2.529094	4.171820	0.625056
C	-2.388359	2.140915	-0.059406
C	-3.122875	1.145312	-0.712340
C	-5.114350	4.829484	0.093799
C	-6.582028	4.842609	-0.354514
H	-7.176302	4.084984	0.174872
H	-7.022132	5.825083	-0.134480
H	-6.677151	4.665746	-1.434961

C	-4.375767	5.960037	-0.649683
H	-4.862162	6.926266	-0.451389
H	-3.328411	6.037028	-0.329855
H	-4.386175	5.783172	-1.734261
C	-5.072367	5.103042	1.610818
H	-5.594316	4.308093	2.161343
H	-4.040928	5.149642	1.983690
H	-5.561385	6.061535	1.838831
C	-6.617978	-0.076594	-1.468221
H	-7.062540	0.786263	-1.980023
H	-7.048315	-0.977974	-1.922508
H	-6.908589	-0.044322	-0.410296
C	-4.749746	-0.117467	-3.142197
H	-5.175094	0.756865	-3.653803
H	-3.663329	-0.113758	-3.296613
H	-5.160867	-1.030183	-3.594851
C	-5.077503	-4.848877	0.314549
C	-6.297646	-4.579825	1.217066
H	-6.824269	-5.520752	1.433737
H	-5.982430	-4.132534	2.170035
H	-7.011068	-3.894320	0.741739
C	-5.549649	-5.471085	-1.015259
H	-4.692669	-5.669761	-1.674087
H	-6.070540	-6.421715	-0.828440
H	-6.237745	-4.802922	-1.549220
C	-4.173545	-5.866139	1.024159
H	-3.297210	-6.127001	0.414600
H	-3.819213	-5.488058	1.993103
H	-4.738650	-6.789896	1.209780
C	-0.141535	-2.798743	0.720069
C	0.375352	-2.884016	2.035008
C	1.222745	-3.949314	2.357313
H	1.626098	-4.029903	3.366005
C	1.560029	-4.915344	1.412218
H	2.212401	-5.745453	1.686930
C	1.088771	-4.795379	0.109495
H	1.391836	-5.526817	-0.641886
C	0.253083	-3.737386	-0.263295
C	-0.150837	-3.590170	-1.720993
H	-0.865591	-2.761196	-1.789947
C	1.071196	-3.205582	-2.570163
H	1.842082	-3.989425	-2.535331
H	0.780938	-3.055281	-3.620294
H	1.515100	-2.270677	-2.194695
C	-0.847331	-4.842067	-2.268452
H	-1.738319	-5.083858	-1.673563
H	-1.164466	-4.677864	-3.308116
H	-0.178185	-5.714543	-2.256908
C	-0.002786	-1.841636	3.073422
H	0.039700	-0.863652	2.563589
C	-1.444564	-2.036533	3.568457
H	-1.535862	-2.995802	4.099055
H	-1.722958	-1.232426	4.265292
H	-2.157858	-2.035725	2.736655

C	0.961836	-1.776913	4.258491
H	2.001162	-1.641103	3.930672
H	0.697282	-0.934946	4.909778
H	0.913623	-2.690072	4.869266
C	-0.207744	2.826980	0.690239
C	0.352979	3.665814	-0.301630
C	1.239650	4.674361	0.091077
H	1.679009	5.324262	-0.668031
C	1.578271	4.854415	1.427830
H	2.274655	5.643133	1.716612
C	1.031839	4.016344	2.396771
H	1.312097	4.156975	3.440363
C	0.138447	2.996720	2.051946
C	-0.482187	2.111039	3.118135
H	-0.629310	1.119040	2.664156
C	-1.875628	2.624806	3.517555
H	-2.546208	2.676552	2.652006
H	-2.329903	1.959296	4.266002
H	-1.800508	3.631792	3.954584
C	0.399029	1.933971	4.355619
H	0.509525	2.873867	4.915063
H	-0.059809	1.206004	5.037918
H	1.402020	1.573541	4.089755
C	0.033124	3.491848	-1.776546
H	-0.667317	2.651556	-1.866420
C	-0.653603	4.734026	-2.360487
H	0.002024	5.615269	-2.302409
H	-0.904482	4.570129	-3.418310
H	-1.582209	4.959972	-1.820712
C	1.291023	3.120794	-2.572115
H	1.737392	2.202612	-2.162685
H	1.042980	2.943197	-3.628629
H	2.044751	3.920856	-2.530485
C	4.904889	-1.565842	0.581084
C	4.069991	-2.756131	0.071282
H	2.994821	-2.543032	0.136662
H	4.300911	-3.043475	-0.956968
H	4.275616	-3.622826	0.716857
C	4.508453	-1.387802	2.056302
H	4.644650	-2.355298	2.562620
H	5.124156	-0.649503	2.580473
H	3.450420	-1.109700	2.151250
C	6.399242	-1.901346	0.497341
H	6.712147	-2.158435	-0.520939
H	7.035498	-1.084861	0.856923
H	6.595773	-2.781235	1.130133
C	5.168389	-0.024314	-2.128343
C	5.016778	-1.425893	-2.742465
H	5.653802	-2.175491	-2.262254
H	3.974644	-1.770472	-2.710192
H	5.317010	-1.365844	-3.799724
C	4.349060	0.916100	-3.033260
H	3.292722	0.615426	-3.050809
H	4.397185	1.964859	-2.730728

H	4.744662	0.841828	-4.058083
C	6.648741	0.377473	-2.161602
H	7.015521	0.285113	-3.196246
H	6.806338	1.416766	-1.852688
H	7.270434	-0.267490	-1.530208
C	4.901334	1.567342	0.561269
C	3.903238	1.793682	1.714793
H	2.869827	1.836259	1.343870
H	3.957561	1.029179	2.493404
H	4.130634	2.765161	2.179332
C	4.743330	2.785114	-0.364482
H	5.479383	2.807383	-1.174909
H	3.732695	2.837831	-0.788807
H	4.890211	3.691238	0.242567
C	6.331959	1.535052	1.114569
H	6.543886	2.504536	1.592618
H	6.464024	0.760927	1.879046
H	7.083640	1.379155	0.332549

INT (with full NON)

157

C	-1.442515	-3.233833	2.641694
C	-0.272365	-2.485915	2.333223
C	0.799238	-2.427396	3.249628
C	0.655388	-3.040039	4.500650
C	-0.504668	-3.725613	4.835561
C	-1.531542	-3.835275	3.900328
N	-0.207151	-1.756183	1.099443
AL	-0.518350	0.110501	0.970663
AU	1.838913	1.167555	0.440268
P	3.570548	1.013632	-1.222743
C	4.458371	-0.671189	-1.036291
C	5.220918	-0.669298	0.298665
C	-0.199683	-2.484822	-0.083722
C	-0.673078	-1.814708	-1.220613
C	-1.107530	-2.415234	-2.382706
C	-0.798407	-3.783511	-2.510489
C	-0.145588	-4.480527	-1.482095
C	0.123683	-3.835209	-0.263355
O	-0.900918	-0.457410	-0.977933
C	-2.138574	0.015193	-1.443664
C	-2.727302	-0.520559	-2.571238
C	-1.963676	-1.594882	-3.366735
C	-3.971581	0.026483	-2.916561
C	-4.561186	1.051500	-2.152887
C	-3.916646	1.525619	-1.003030
C	-2.683689	0.985913	-0.601671
N	-1.956715	1.262012	0.548498
C	-2.267856	2.476691	1.240938
C	-3.032027	2.451103	2.427444
C	-3.352078	3.661113	3.052540

C	-2.927067	4.877289	2.528748
C	-2.145752	4.891869	1.376968
C	-1.791989	3.706512	0.724354
C	-2.929040	-2.513955	-4.124631
C	-1.061246	-0.863163	-4.386752
O	-0.132175	0.763361	2.643736
C	0.884338	1.513653	2.284145
O	1.434316	2.357112	2.978321
C	2.923375	1.210874	-3.009003
C	2.301243	-0.116199	-3.466275
C	4.773013	2.448751	-0.795795
C	4.131343	3.774020	-1.240826
C	1.776538	2.239072	-2.986324
C	3.989970	1.629156	-4.029808
C	4.906321	2.545686	0.738286
C	6.168393	2.321588	-1.421571
C	3.389427	-1.777080	-0.927259
C	5.442504	-1.004459	-2.166060
H	-4.364147	2.300478	-0.385502
H	-4.491832	-0.350713	-3.794579
H	-1.090202	-4.308488	-3.415335
H	0.511827	-4.392651	0.588512
H	-2.373838	-3.242114	-4.729002
H	-3.551308	-1.932116	-4.815477
H	-3.584934	-3.059491	-3.434475
H	-0.419300	-1.579862	-4.917554
H	-0.424935	-0.127437	-3.884490
H	-1.683908	-0.331417	-5.119201
C	-5.921117	1.614582	-2.590077
C	0.238201	-5.959983	-1.617516
H	5.510476	3.436310	0.967714
H	3.928831	2.675330	1.225528
H	5.401901	1.684238	1.191060
H	4.753317	4.594798	-0.854066
H	4.078383	3.887117	-2.327951
H	3.125914	3.896820	-0.818357
H	6.744530	3.224478	-1.167547
H	6.723394	1.462536	-1.028386
H	6.137565	2.244356	-2.513747
H	1.797231	0.066288	-4.425751
H	3.044343	-0.901036	-3.634931
H	1.549127	-0.484688	-2.757839
H	1.342967	2.296387	-3.996158
H	0.983929	1.928984	-2.292061
H	2.101223	3.244251	-2.706272
H	3.521882	1.656848	-5.025697
H	4.396265	2.626807	-3.834107
H	4.823318	0.918716	-4.075712
H	5.923907	-1.965945	-1.930559
H	4.948483	-1.119297	-3.136611
H	6.234230	-0.254488	-2.268671
H	5.584412	-1.691135	0.480802
H	6.092697	-0.007839	0.288575
H	4.570668	-0.392800	1.139187

H	3.893708	-2.711982	-0.641689
H	2.648507	-1.547616	-0.151123
H	2.853081	-1.963930	-1.859639
C	-3.527284	1.149716	3.021710
H	-3.948007	3.647240	3.966519
H	-3.191269	5.812535	3.024512
H	-1.792445	5.845649	0.982995
C	-0.900560	3.770055	-0.503846
C	2.138282	-1.817366	2.880381
H	1.475595	-2.989201	5.217824
H	-0.605178	-4.192135	5.816658
H	-2.424168	-4.403481	4.159026
C	-2.588434	-3.398701	1.647724
C	-3.365939	-4.707113	1.831494
C	-3.549044	-2.203982	1.665969
H	-2.151675	-3.442112	0.643319
H	-4.057929	-4.841663	0.988823
H	-2.693243	-5.574846	1.863752
H	-3.969155	-4.706812	2.750669
H	-4.376218	-2.363228	0.959793
H	-3.973592	-2.053979	2.668690
H	-3.042869	-1.277771	1.368475
C	2.712882	-0.899638	3.963777
C	3.125922	-2.946097	2.539856
H	1.988651	-1.213747	1.972487
H	4.105986	-2.538164	2.257993
H	3.268384	-3.604088	3.409281
H	2.756235	-3.559407	1.707039
H	3.624167	-0.405651	3.596900
H	1.995676	-0.120709	4.248066
H	2.987600	-1.462561	4.867103
C	-0.106831	-6.532091	-2.998173
C	1.758676	-6.104660	-1.400898
C	-0.511618	-6.784088	-0.550915
H	2.057425	-7.159325	-1.488759
H	2.313447	-5.523407	-2.150951
H	2.059061	-5.746673	-0.407512
H	-0.243514	-7.847355	-0.634327
H	-0.265698	-6.451327	0.465693
H	-1.598414	-6.689094	-0.682548
H	0.208942	-7.583247	-3.048361
H	-1.187803	-6.499791	-3.192936
H	0.407357	-5.987198	-3.802165
C	-6.416490	2.731929	-1.661955
C	-5.802406	2.191673	-4.014538
C	-6.966684	0.481463	-2.580820
H	-7.383987	3.105431	-2.024521
H	-6.561451	2.373323	-0.633521
H	-5.717339	3.579304	-1.636705
H	-6.770052	2.600376	-4.340472
H	-5.058720	3.000380	-4.043534
H	-5.496586	1.424532	-4.737762
H	-7.948730	0.867494	-2.891232
H	-6.688346	-0.330585	-3.265175

H	-7.065196	0.055654	-1.572620
C	-3.132619	0.991756	4.494282
C	-5.040683	0.991208	2.817094
H	-3.037765	0.342782	2.467243
H	-5.387513	0.028580	3.218953
H	-5.592069	1.793215	3.329646
H	-5.297962	1.029078	1.749539
H	-3.432202	-0.001142	4.859530
H	-2.045674	1.087868	4.610512
H	-3.621602	1.743449	5.130650
C	0.342694	4.634791	-0.261433
H	-0.553729	2.745049	-0.701457
C	-1.666943	4.254593	-1.744304
H	0.997990	4.602568	-1.142572
H	0.080050	5.687911	-0.090760
H	0.908258	4.279226	0.610678
H	-1.008261	4.259429	-2.625296
H	-2.524061	3.604555	-1.959313
H	-2.039984	5.278586	-1.593507

PC (with full NON)

157

C	-0.621046	3.188351	-0.147846
C	0.155120	2.417585	-1.047914
C	-0.202283	2.343264	-2.416213
C	-1.316560	3.061411	-2.861047
C	-2.063578	3.851114	-1.989609
C	-1.713747	3.908146	-0.646224
N	1.271018	1.670127	-0.564126
AL	1.175062	-0.166869	-0.081258
N	2.220476	-1.724724	-0.305774
C	1.820940	-2.907582	-1.001398
C	1.193855	-3.956668	-0.293281
C	0.850371	-5.124133	-0.984859
C	1.103806	-5.253459	-2.346277
C	1.694144	-4.200731	-3.042000
C	2.061817	-3.018074	-2.391260
C	3.451427	-1.754005	0.334575
C	3.796402	-0.594246	1.037012
C	5.031287	-0.290849	1.562931
C	5.990696	-1.317095	1.503130
C	5.684169	-2.555796	0.919809
C	4.423864	-2.764728	0.331336
O	2.767138	0.362323	1.035314
C	3.219593	1.668039	0.769587
C	4.432194	2.097573	1.267911
C	5.232629	1.117929	2.142122
C	4.789502	3.411553	0.938851
C	3.949794	4.217741	0.146660
C	2.752759	3.696395	-0.362763
C	2.365665	2.373871	-0.079447

C	6.713680	1.495509	2.201281
C	4.640109	1.150515	3.573665
O	-0.192189	-0.327339	1.214730
C	-1.032914	-0.546965	0.238757
AU	-3.064240	-0.497153	0.416289
P	-5.386520	-0.259916	0.425595
C	-5.810693	1.113568	-0.844582
C	-5.444290	2.480033	-0.239943
O	-0.461326	-0.650286	-0.922682
C	-6.170926	-1.922890	-0.108113
C	-5.897033	-2.137023	-1.606280
C	-6.015148	0.236242	2.164441
C	-5.943749	-0.991716	3.087537
C	-5.429065	-3.060071	0.623716
C	-7.678823	-2.033884	0.150765
C	-5.032175	1.264678	2.757845
C	-7.440794	0.803741	2.188843
C	-4.895337	0.945023	-2.074746
C	-7.279350	1.132067	-1.288359
H	2.107627	4.294344	-1.001941
H	5.731034	3.814982	1.305992
H	6.981038	-1.139856	1.911855
H	4.199833	-3.699347	-0.180922
H	7.262149	0.797660	2.846477
H	6.837375	2.497637	2.630869
H	7.171143	1.483177	1.203702
H	5.172560	0.436743	4.217241
H	3.576003	0.882794	3.566236
H	4.741218	2.158249	3.999595
C	4.372264	5.666250	-0.139464
C	6.708522	-3.698108	0.865028
H	-6.138852	-0.651192	4.115352
H	-6.692595	-1.753153	2.847020
H	-4.946531	-1.450927	3.072690
H	-5.334554	1.462672	3.797303
H	-4.007674	0.869845	2.768883
H	-5.022571	2.218190	2.225609
H	-7.719777	0.997459	3.235871
H	-7.520263	1.754454	1.650204
H	-8.177783	0.107899	1.772687
H	-5.787881	-4.017519	0.216390
H	-4.345351	-2.999850	0.450459
H	-5.602662	-3.071149	1.702021
H	-6.199030	-3.163952	-1.860379
H	-6.468775	-1.458315	-2.247051
H	-4.828766	-2.035476	-1.839547
H	-8.029365	-3.000268	-0.243045
H	-7.922146	-2.011216	1.218824
H	-8.250099	-1.245050	-0.351086
H	-5.087648	1.788876	-2.754789
H	-3.834829	0.979826	-1.790229
H	-5.074361	0.021484	-2.629875
H	-7.427163	1.990345	-1.961609
H	-7.557707	0.230918	-1.846107

H	-7.973038	1.246661	-0.448036
H	-5.534751	3.232400	-1.037593
H	-6.110592	2.781984	0.574425
H	-4.404158	2.500775	0.111250
C	0.883018	-3.848222	1.189153
H	0.372262	-5.944238	-0.446048
H	0.832043	-6.170795	-2.870823
H	1.872474	-4.304133	-4.112355
C	2.724777	-1.889935	-3.163444
C	0.625593	1.511322	-3.380965
H	-1.608631	3.006475	-3.908988
H	-2.925664	4.409329	-2.358256
H	-2.310983	4.511159	0.040325
C	-0.333707	3.241798	1.344587
C	0.120873	4.641630	1.786601
C	-1.548873	2.784889	2.163511
H	0.482716	2.540114	1.555426
H	0.353627	4.646771	2.861151
H	1.019467	4.955910	1.241292
H	-0.670263	5.385018	1.608308
H	-1.298017	2.755754	3.233538
H	-2.399104	3.470902	2.037799
H	-1.870870	1.780750	1.858229
C	-0.117001	1.143017	-4.666476
C	1.955937	2.209193	-3.708919
H	0.865971	0.569279	-2.864789
H	0.495744	0.460039	-5.267729
H	-1.072302	0.646013	-4.449143
H	-0.321767	2.028068	-5.286312
H	2.570924	1.575557	-4.364474
H	1.768958	3.159579	-4.230524
H	2.530849	2.424110	-2.800706
C	3.374859	6.398698	-1.047162
C	4.463110	6.434222	1.195226
C	5.749289	5.679133	-0.831897
H	3.724897	7.425732	-1.220046
H	3.279089	5.906758	-2.025011
H	2.376841	6.458316	-0.591327
H	6.061951	6.713914	-1.034940
H	6.521340	5.208483	-0.209640
H	5.707024	5.136713	-1.786708
H	4.762522	7.477446	1.016220
H	3.490354	6.436597	1.706772
H	5.198642	5.978207	1.870643
C	8.024891	-3.339706	1.567545
C	6.118501	-4.943929	1.555775
C	7.022476	-4.032216	-0.607363
H	7.751938	-4.853413	-0.663508
H	6.120663	-4.339689	-1.152510
H	7.444785	-3.157385	-1.121159
H	8.711666	-4.195752	1.515266
H	8.521372	-2.484005	1.089274
H	7.865278	-3.100332	2.628068
H	6.840844	-5.772852	1.527364

H	5.879993	-4.728421	2.606634
H	5.197441	-5.280507	1.062512
C	1.721469	-4.838414	2.012169
H	1.154402	-2.833503	1.508330
C	-0.616028	-4.034393	1.465162
H	-0.828150	-3.878820	2.532566
H	-1.217607	-3.317364	0.890758
H	-0.949720	-5.048035	1.199568
H	1.508485	-4.723473	3.084763
H	1.493184	-5.877173	1.731121
H	2.795050	-4.669117	1.856766
C	2.229019	-1.777411	-4.608060
H	2.470037	-0.952109	-2.645970
C	4.257213	-2.014840	-3.136782
H	2.624071	-0.861582	-5.068428
H	2.572367	-2.622164	-5.222245
H	1.132906	-1.743238	-4.656180
H	4.718440	-1.175779	-3.677721
H	4.644384	-2.018711	-2.111607
H	4.570813	-2.950403	-3.62407
